# Supplementary figures and images for: Xanthomonas citri subsp. citri type III effector PthA4 directs the dynamical expression of a putative citrus carbohydrate-binding protein gene for canker formation
Source: eLife. 2024 Aug 13;13:RP91684. doi: 10.7554/eLife.91684 (PMC11321762; doi:10.7554/eLife.91684)

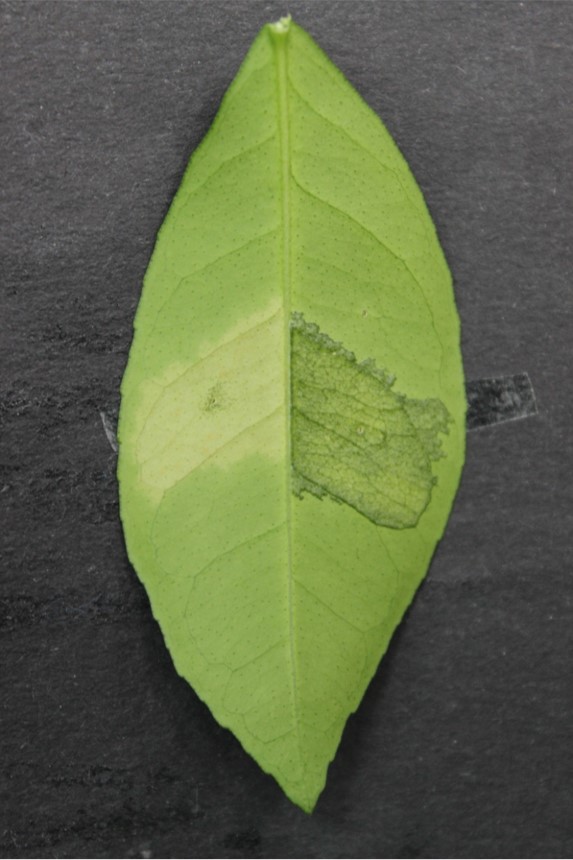

Supplement: Figure 1—source data 2. [file elife-91684-fig1-data2.zip › Figure 1-source data 2/Figure 1-source data 2.jpg]

**B**

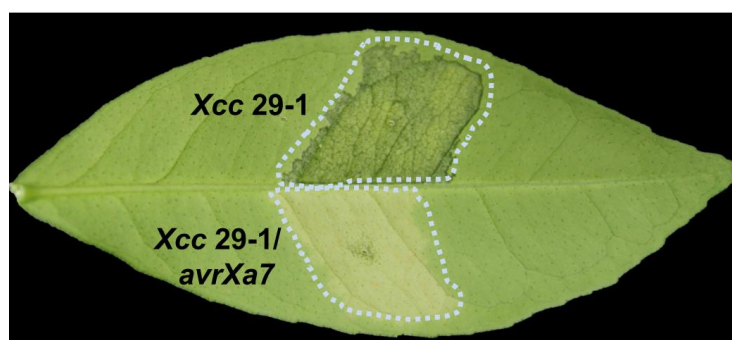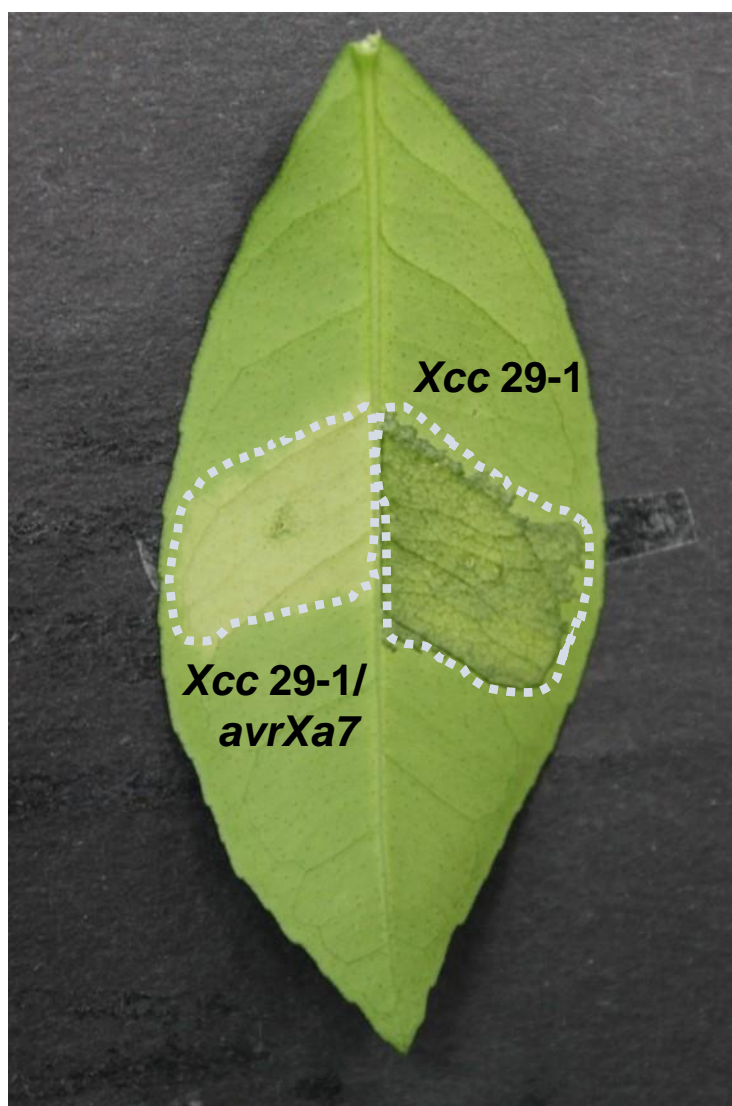

**Figure 1**

Supplement: Figure 1—source data 3. [file elife-91684-fig1-data3.pdf]

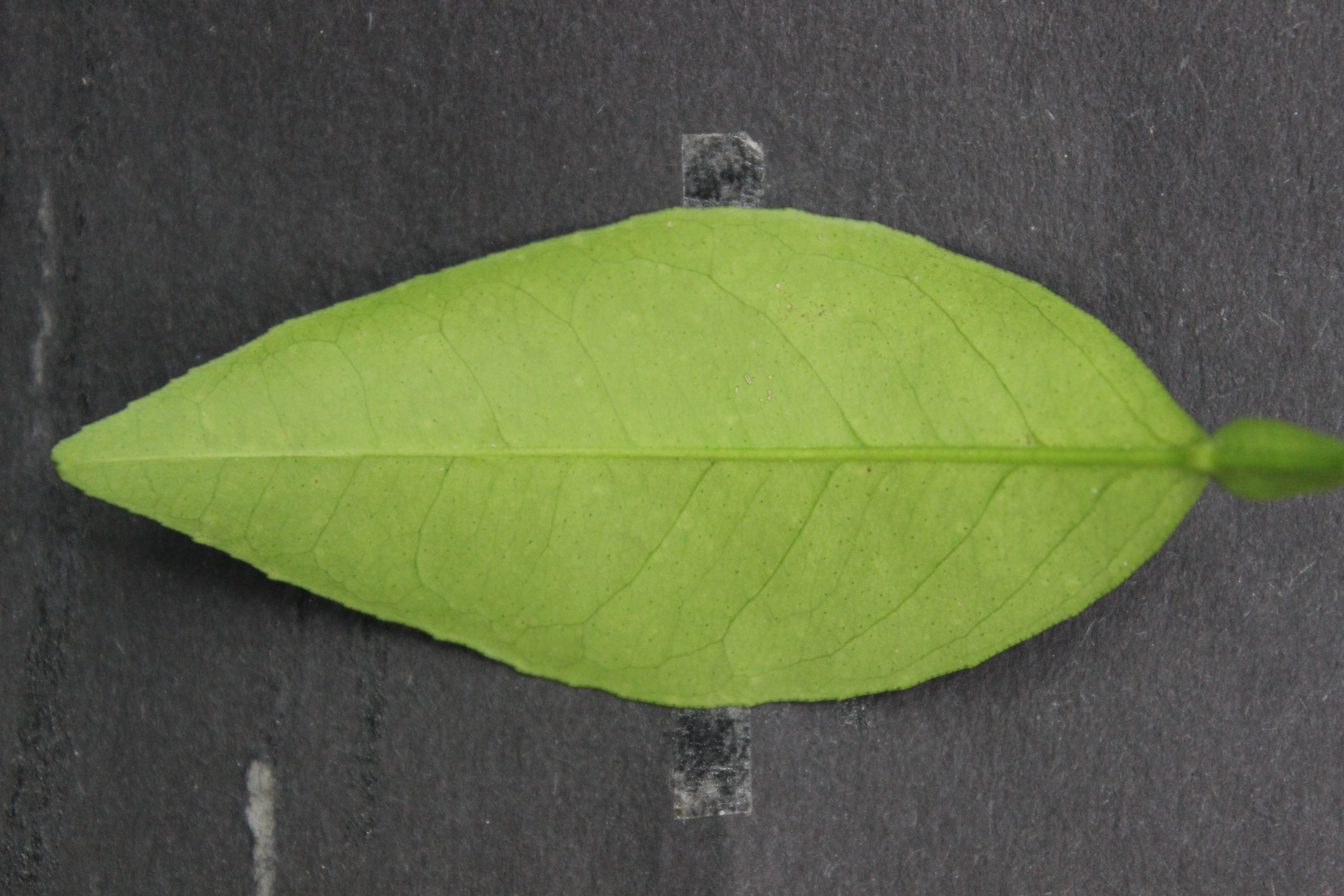

Supplement: Figure 1—source data 4. [file elife-91684-fig1-data4.zip › Figure 1-source data 4/Figure 1-source data 4.JPG]

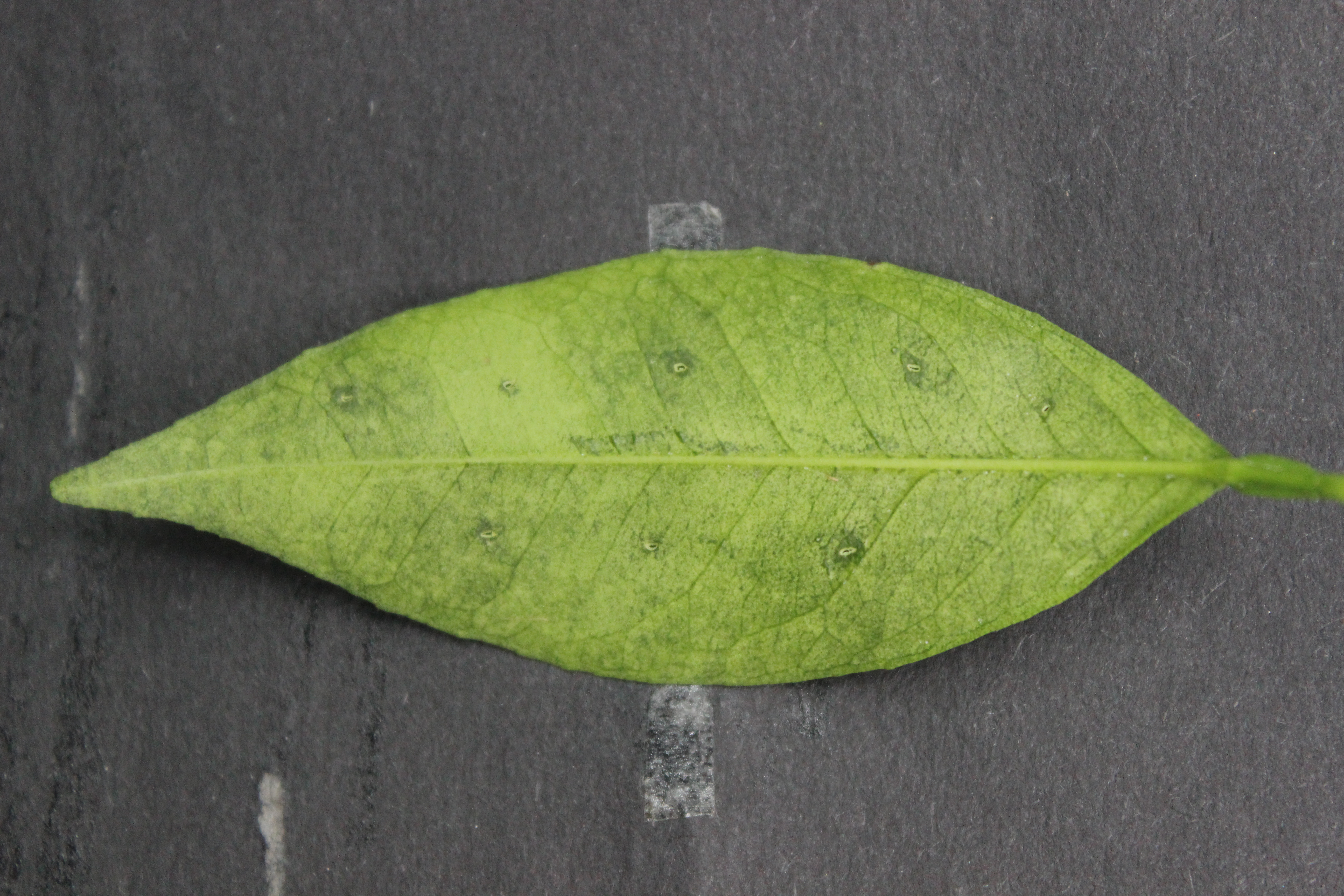

Supplement: Figure 1—source data 5. [file elife-91684-fig1-data5.zip › Figure 1-source data 5/Figure 1-source data 5.JPG]

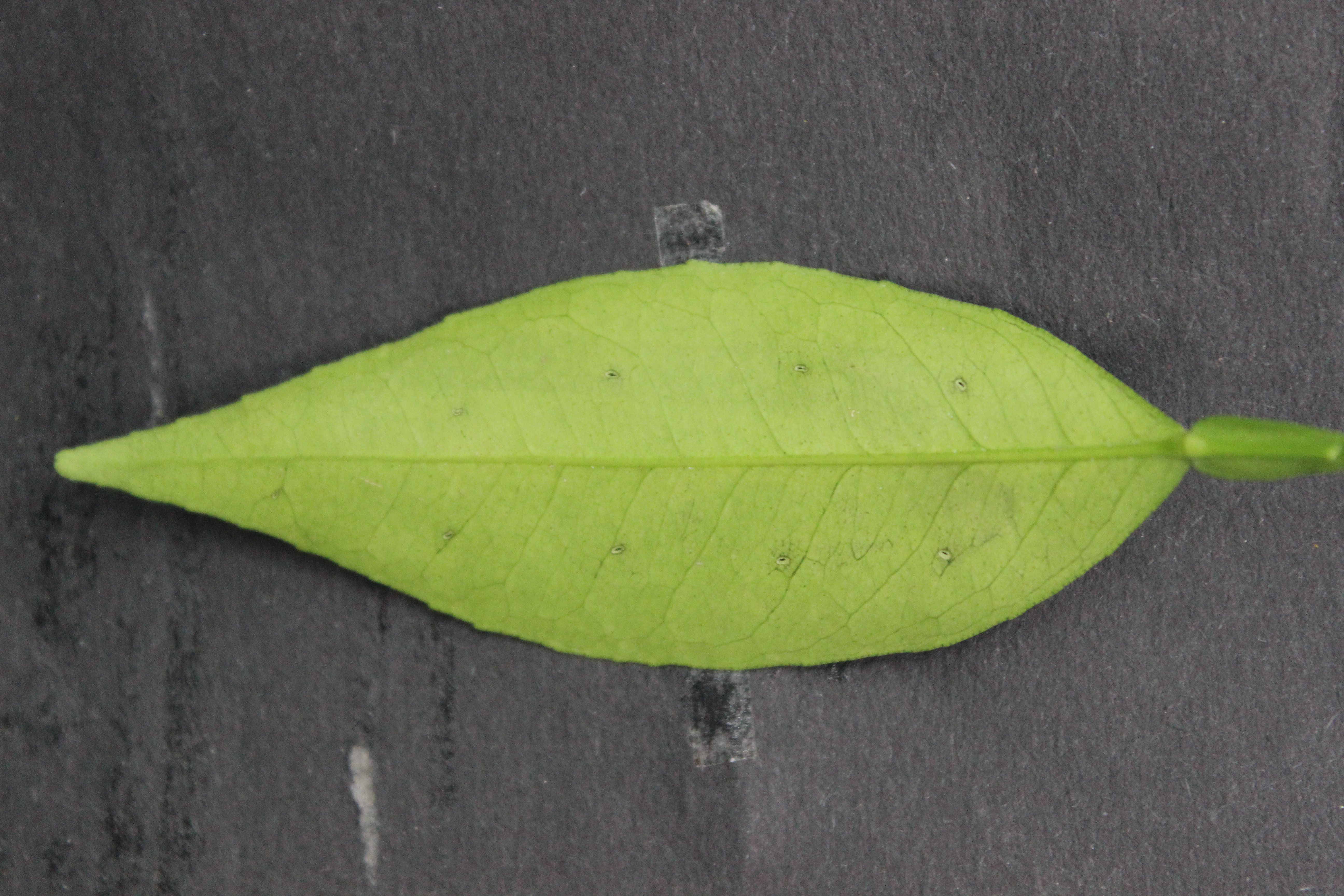

Supplement: Figure 1—source data 6. [file elife-91684-fig1-data6.zip › Figure 1-source data 6/Figure 1-source data 6.JPG]

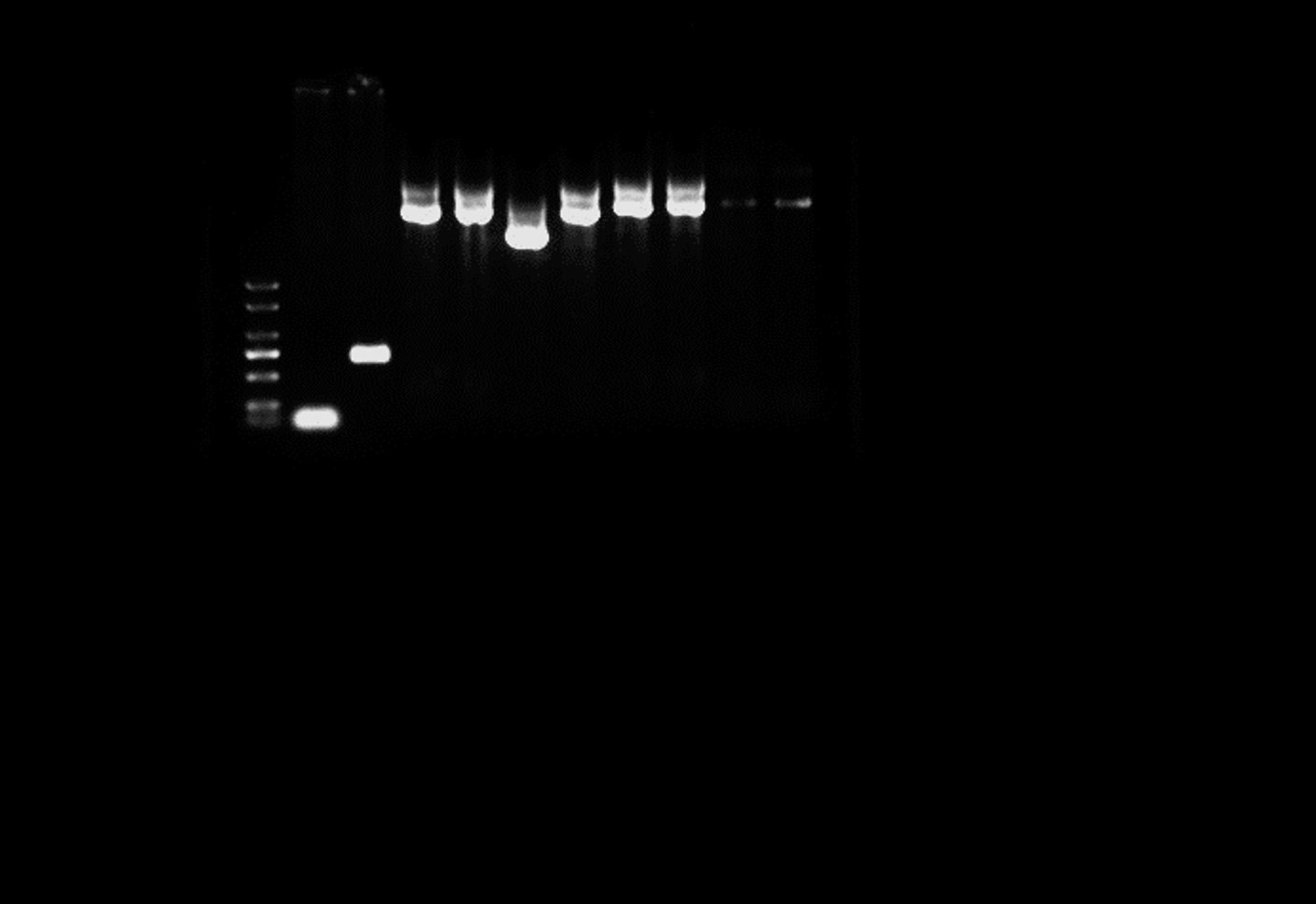

Supplement: Figure 2—source data 2. [file elife-91684-fig2-data2.zip › Figure 2-source data 2/Figure 2-source data 2.jpg]

**B**

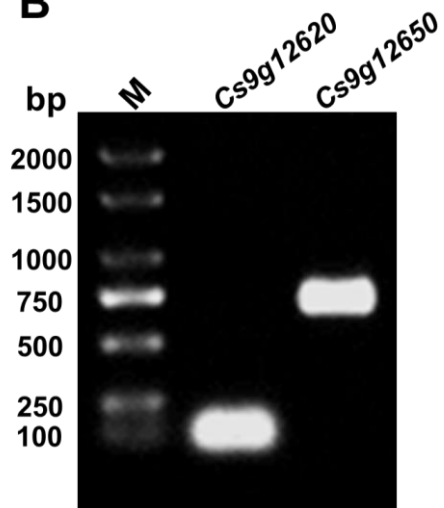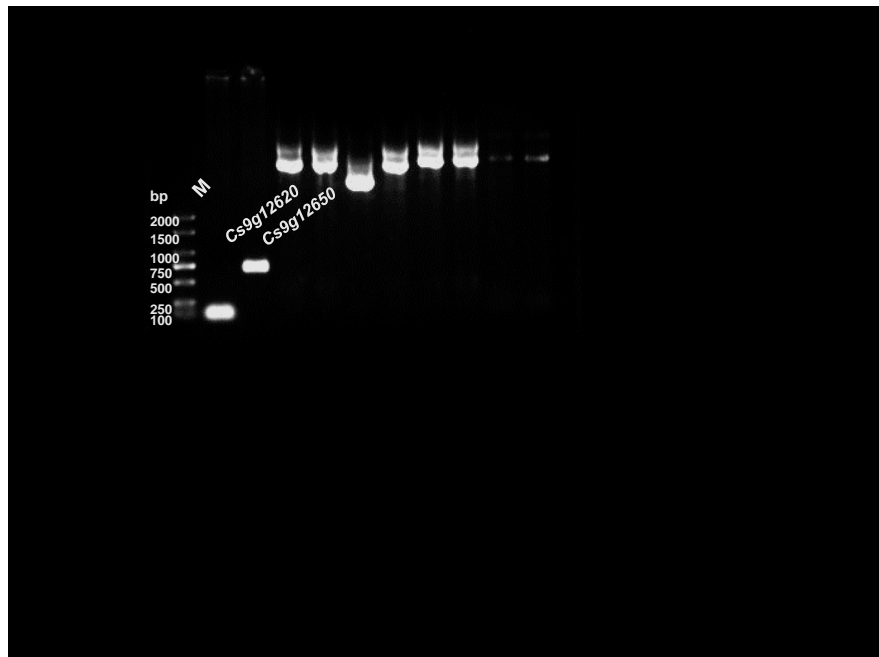

**Figure 2**

Supplement: Figure 2—source data 3. [file elife-91684-fig2-data3.pdf]

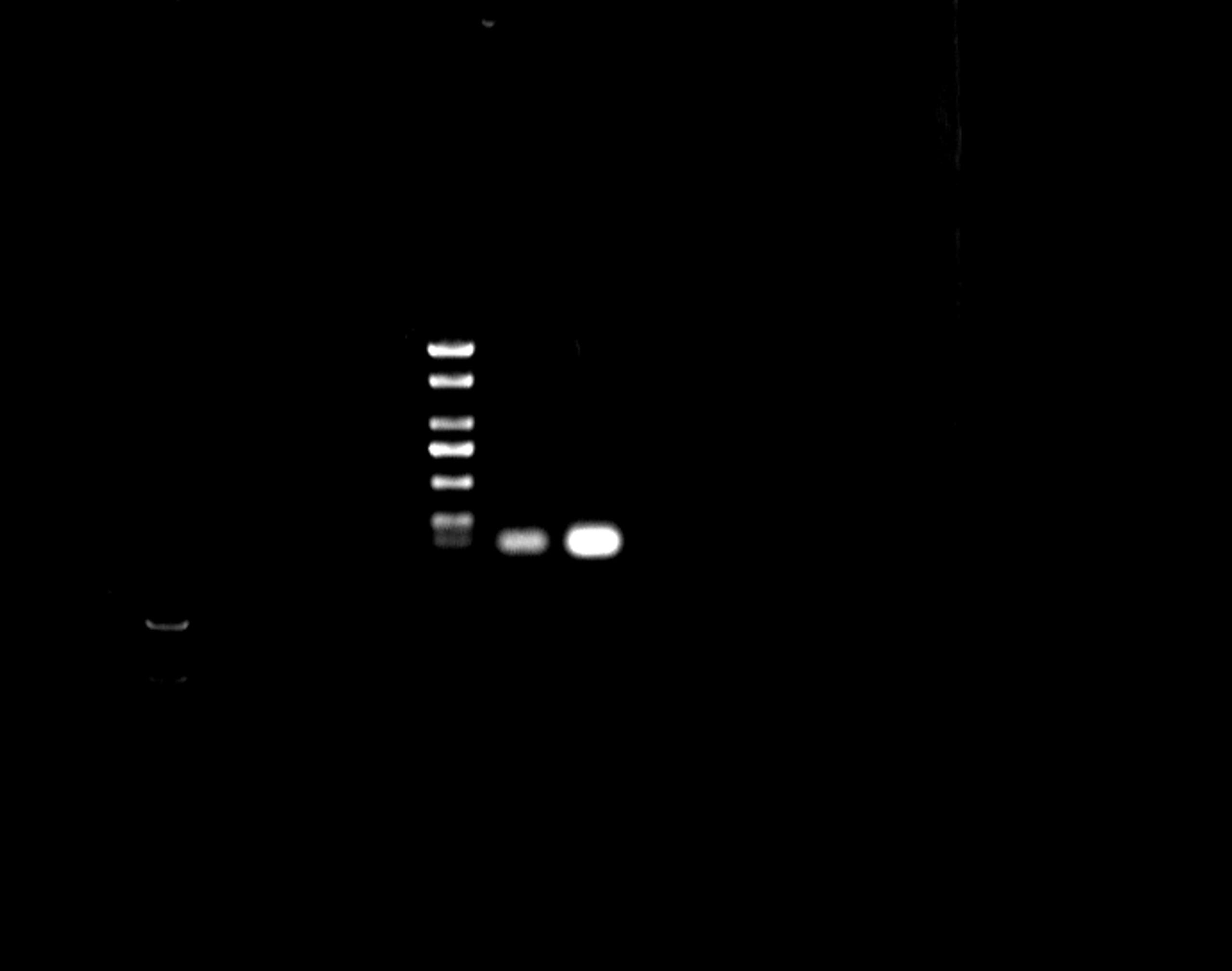

Supplement: Figure 2—source data 4. [file elife-91684-fig2-data4.zip › Figure 2-source data 4/Figure 2-source data 4.jpg]

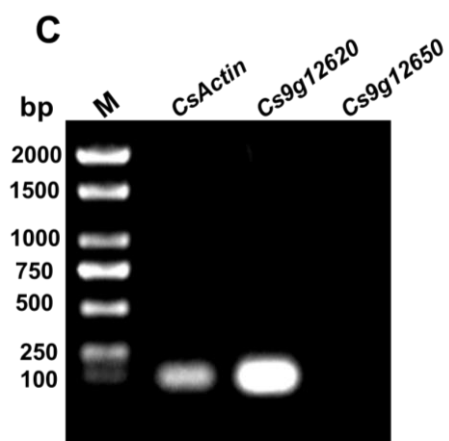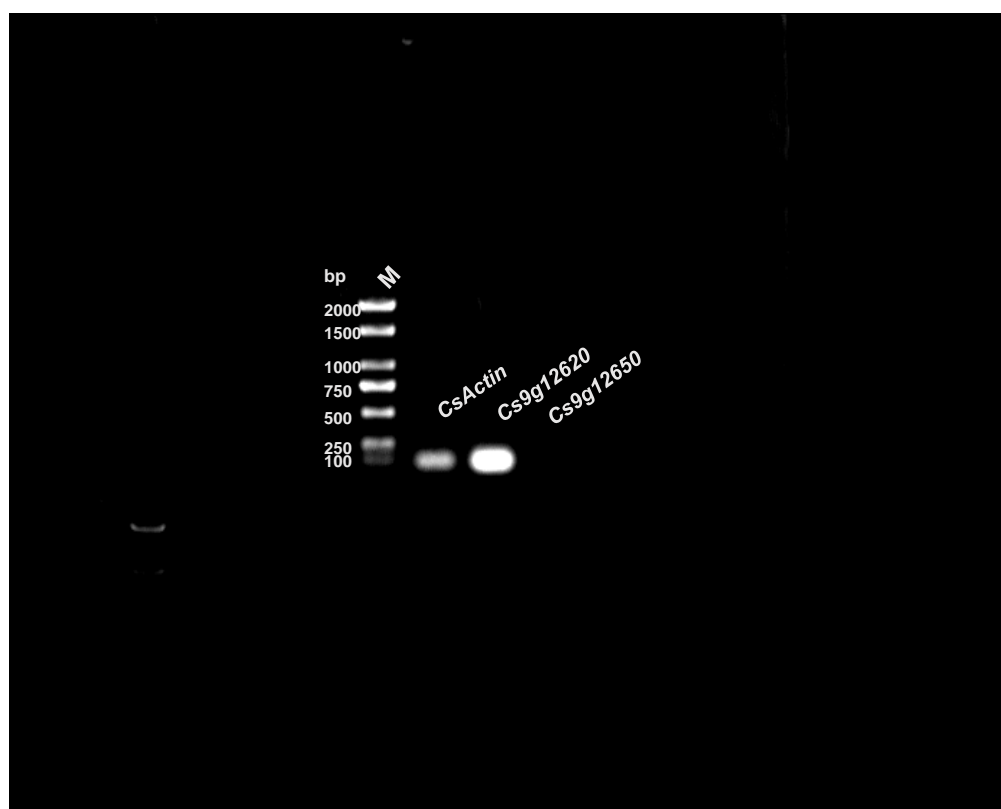

**Figure 2**

Supplement: Figure 2—source data 5. [file elife-91684-fig2-data5.pdf]

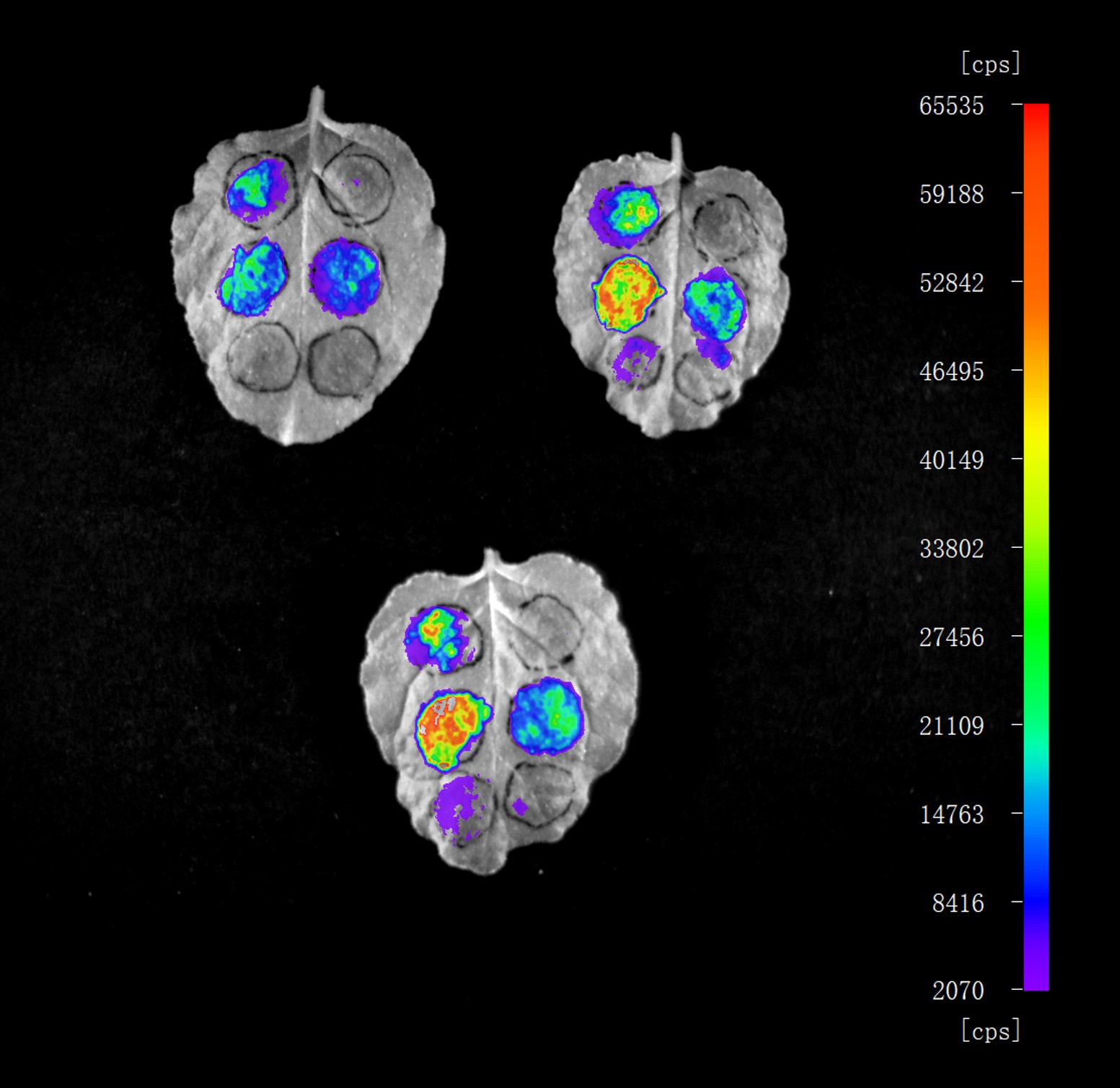

Supplement: Figure 2—source data 6. [file elife-91684-fig2-data6.zip › Figure 2-source data 6/Figure 2-source data 6.jpg]

D

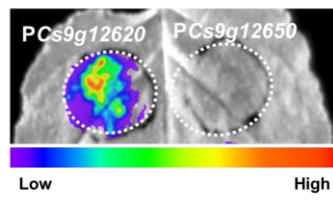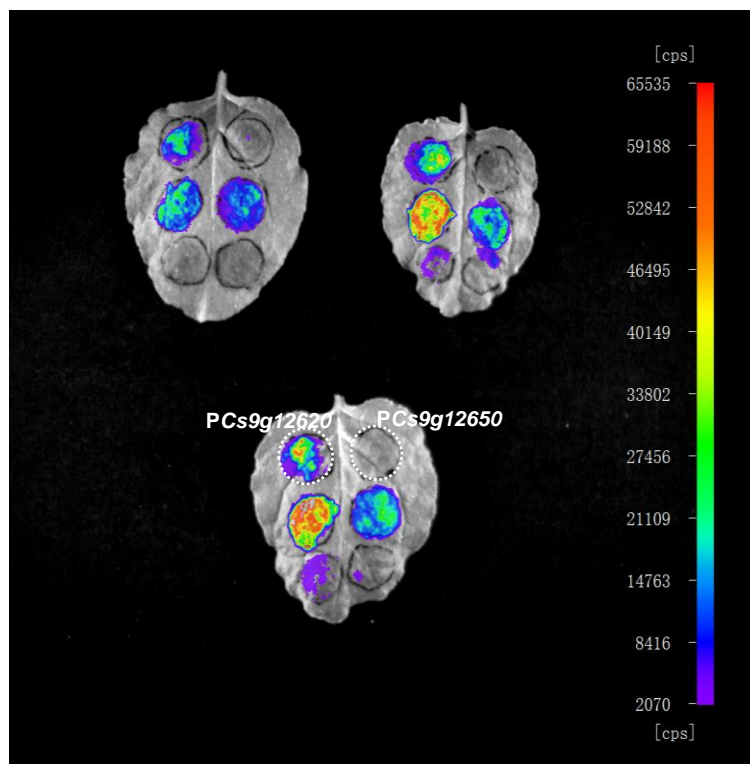

Figure 2

Supplement: Figure 2—source data 7. [file elife-91684-fig2-data7.pdf]

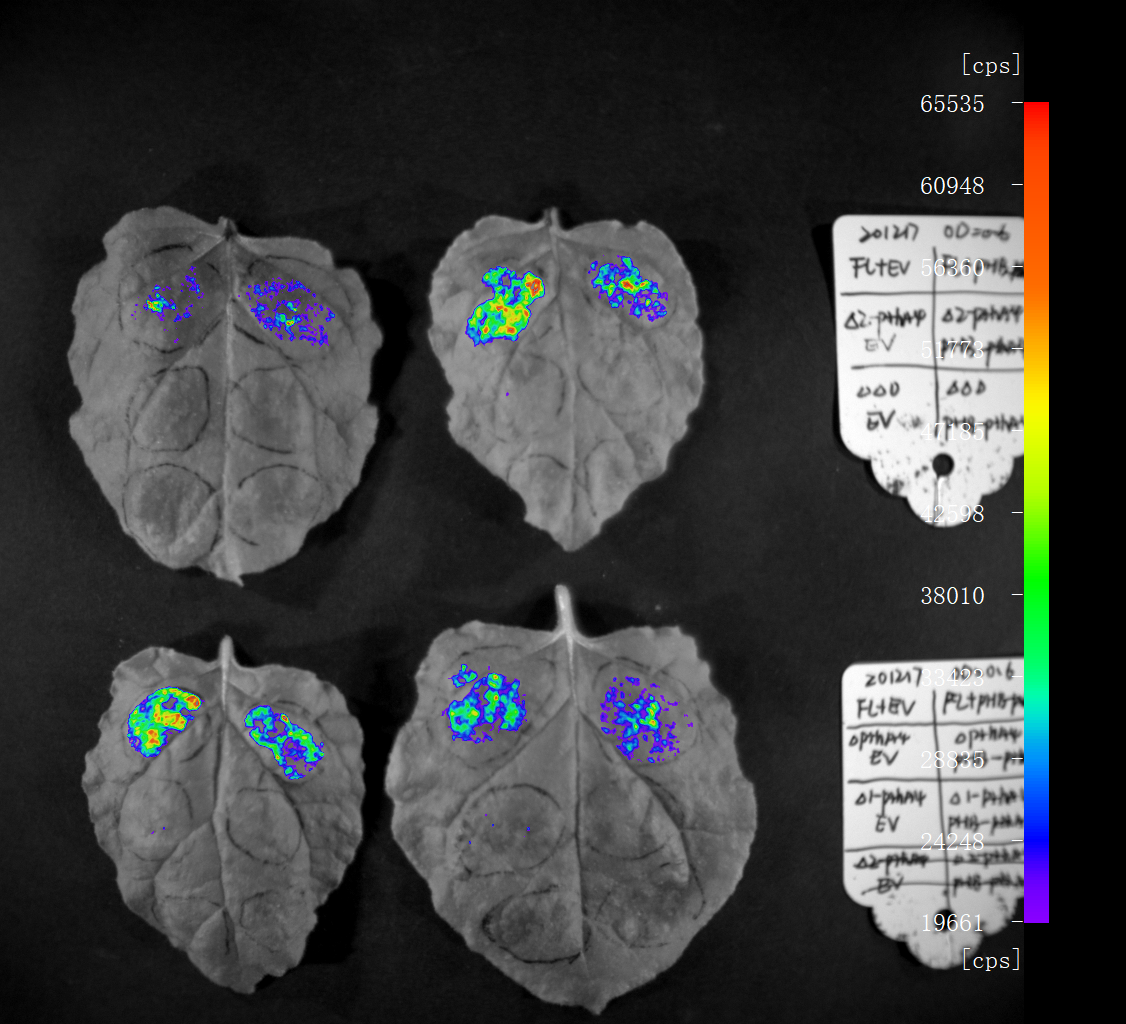

Supplement: Figure 2—source data 8. [file elife-91684-fig2-data8.zip › Figure 2-source data 8/Figure 2-source data 8.jpg]

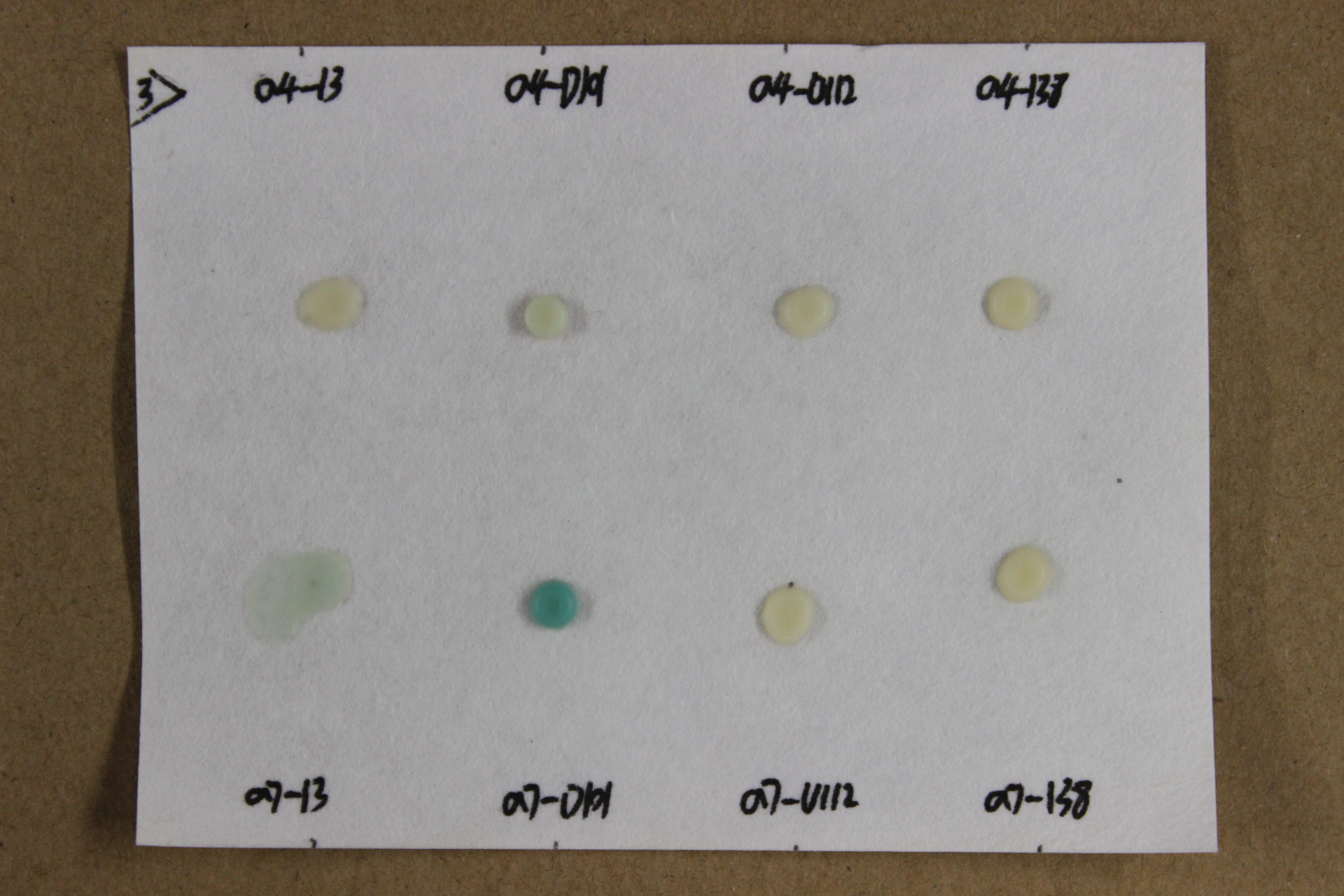

Supplement: Figure 3—source data 2. [file elife-91684-fig3-data2.zip › Figure 3- source data 2/Figure 3- source data 2.JPG]

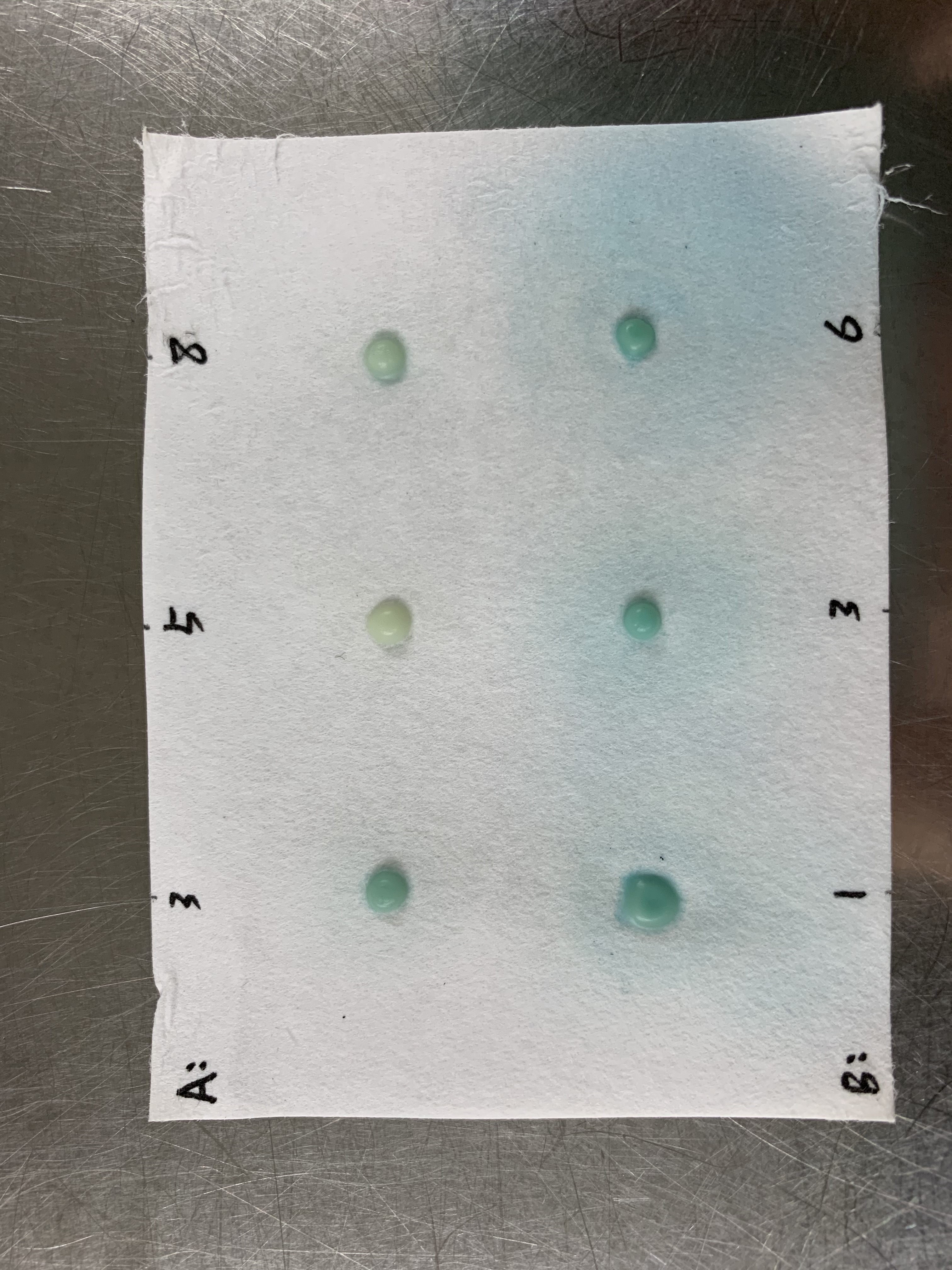

Supplement: Figure 3—source data 3. [file elife-91684-fig3-data3.zip › Figure 3- source data 3/Figure 3- source data 3.JPG]

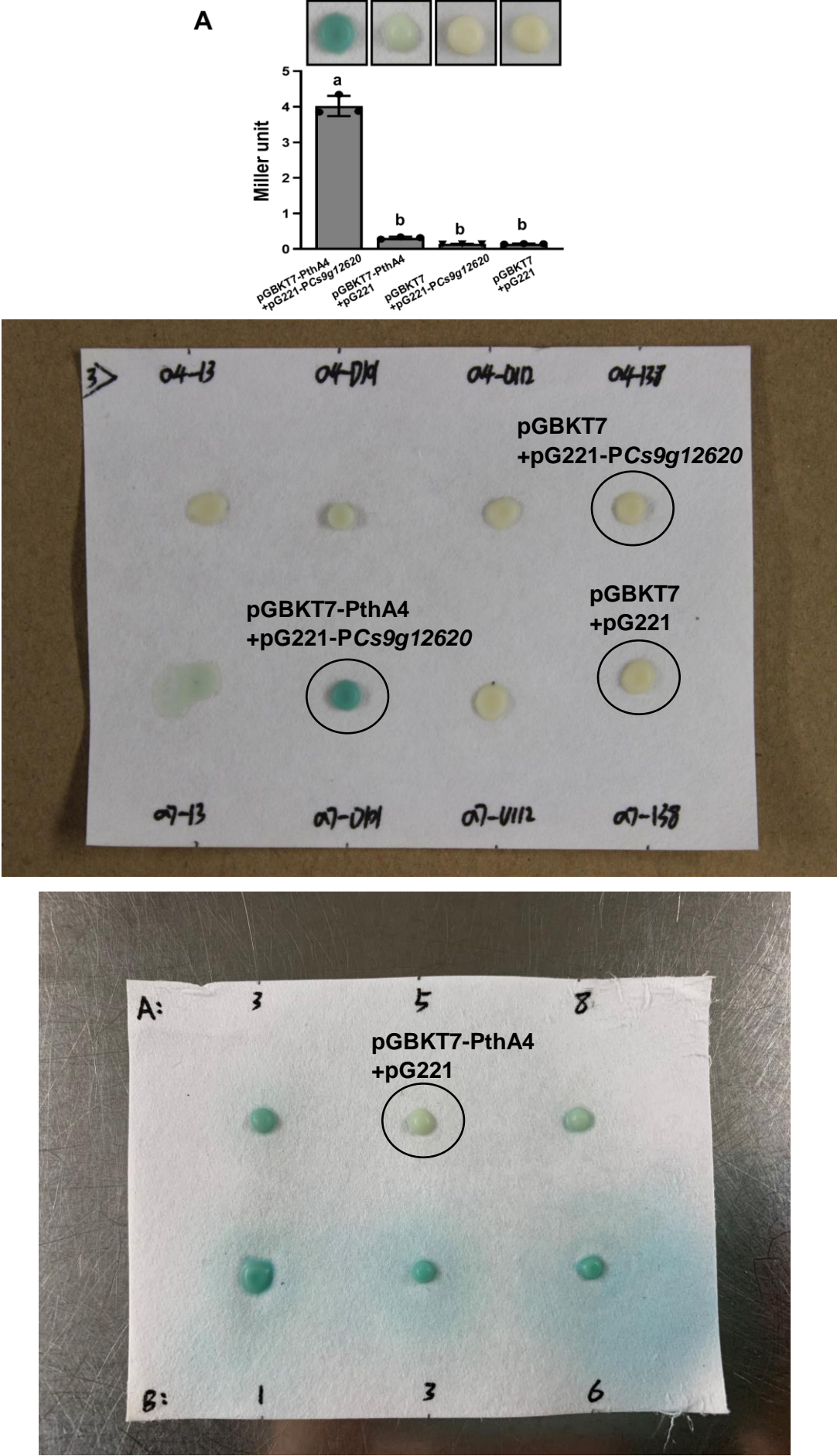

Figure 3

Supplement: Figure 3—source data 4. [file elife-91684-fig3-data4.pdf]

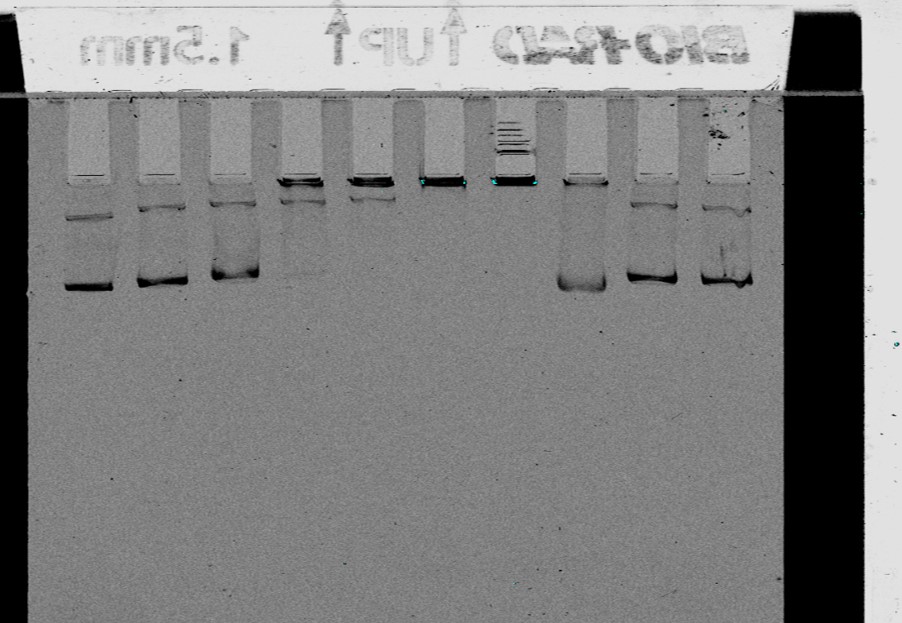

Supplement: Figure 3—source data 5. [file elife-91684-fig3-data5.zip › Figure 3- source data 5/Figure 3- source data 5.jpg]

**B**

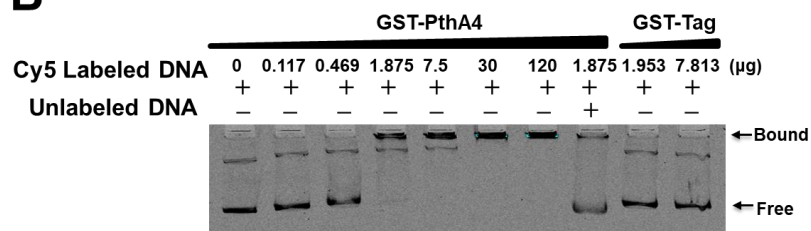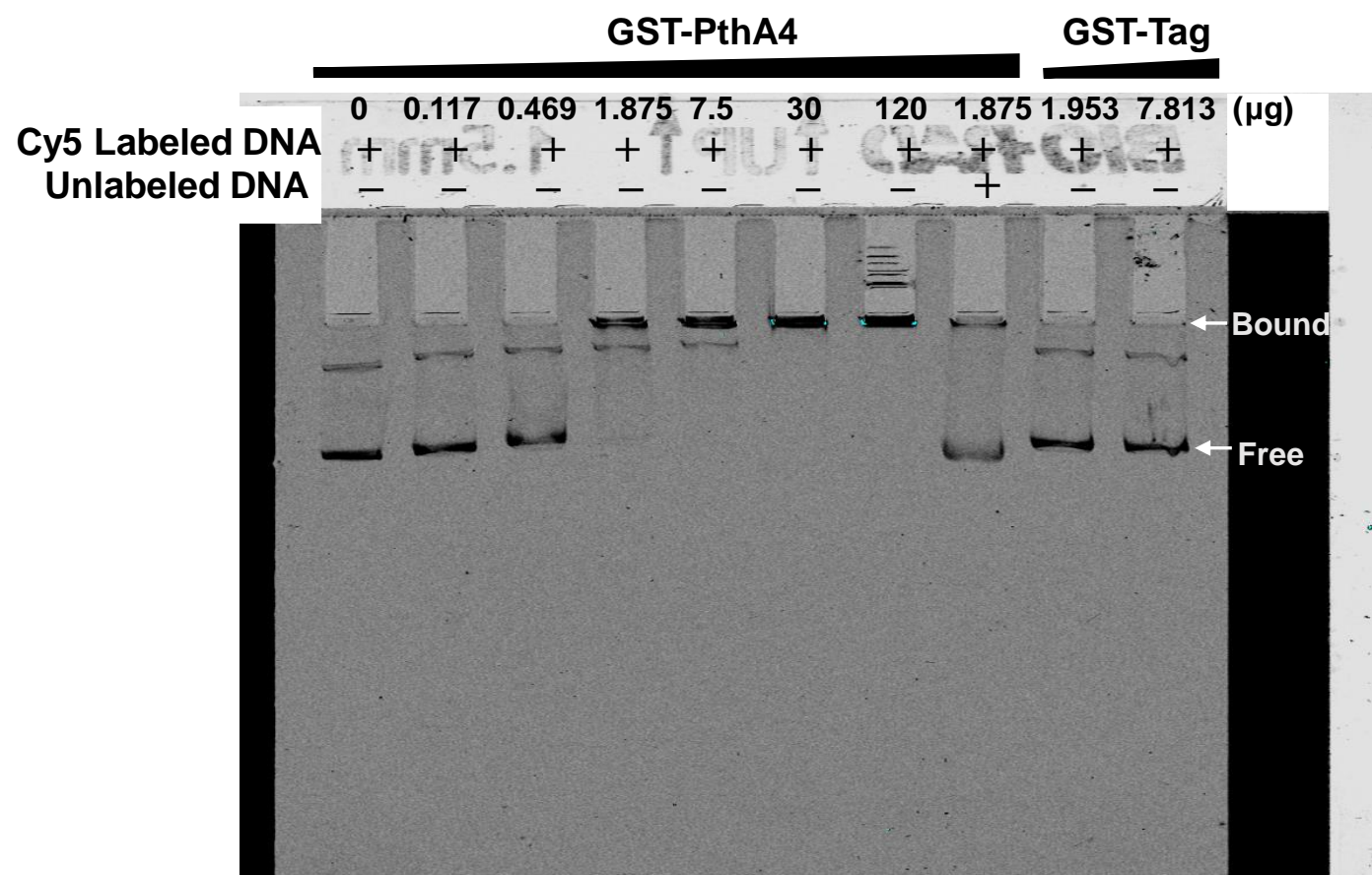

**Figure 3**

Supplement: Figure 3—source data 6. [file elife-91684-fig3-data6.pdf]

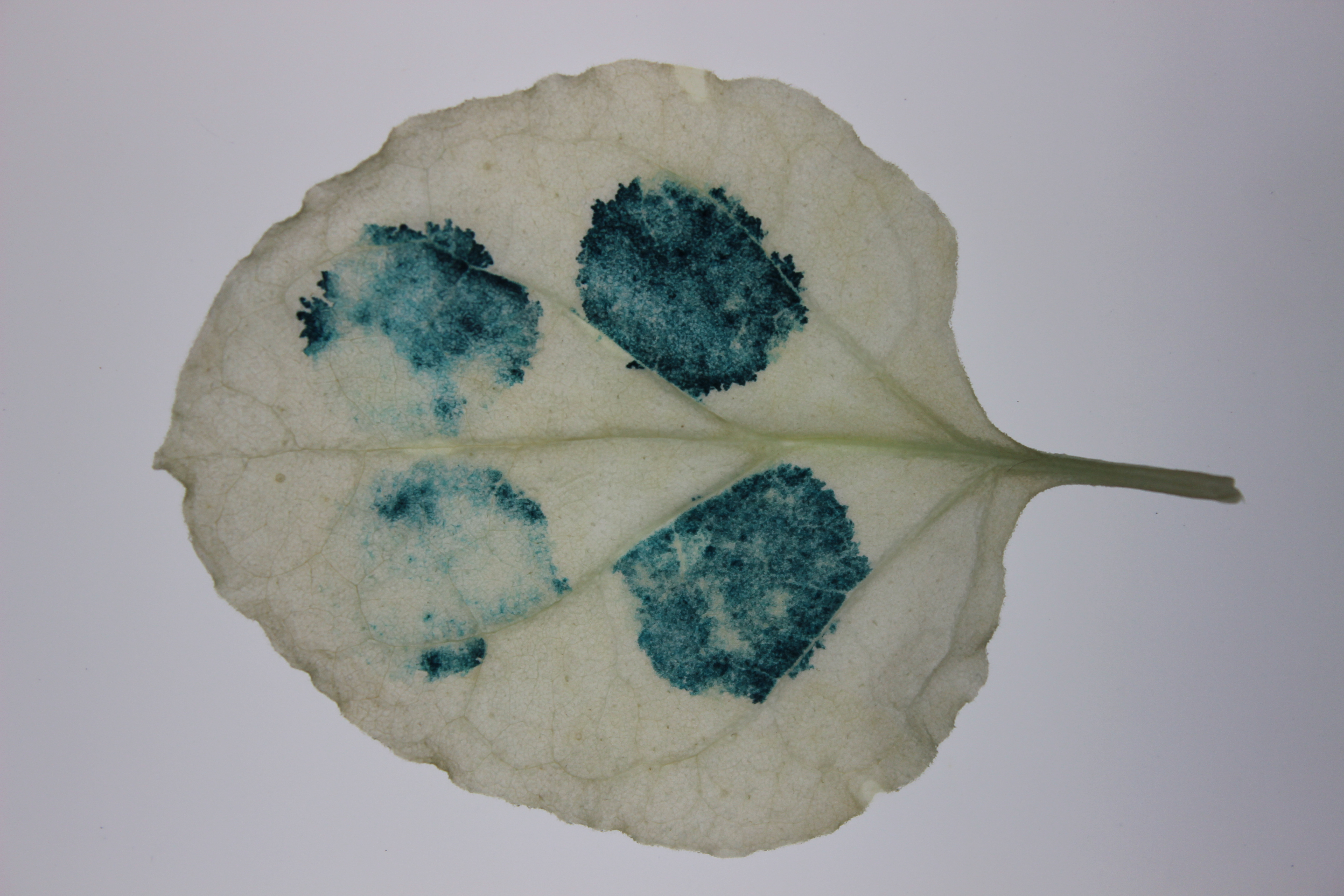

Supplement: Figure 3—source data 7. [file elife-91684-fig3-data7.zip › Figure 3- source data 7/Figure 3- source data 7.JPG]

**C**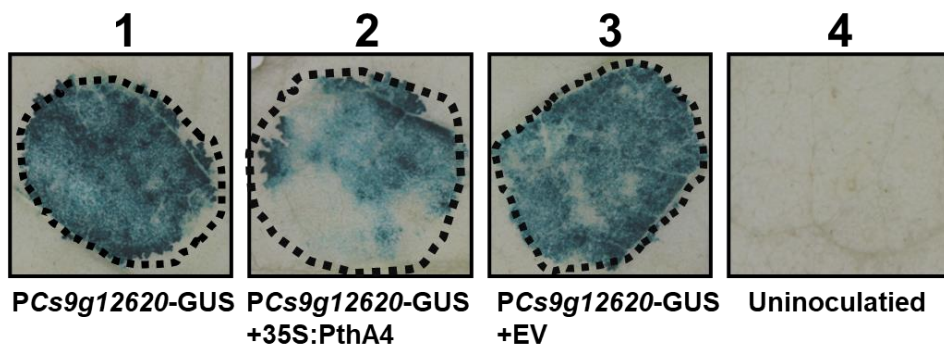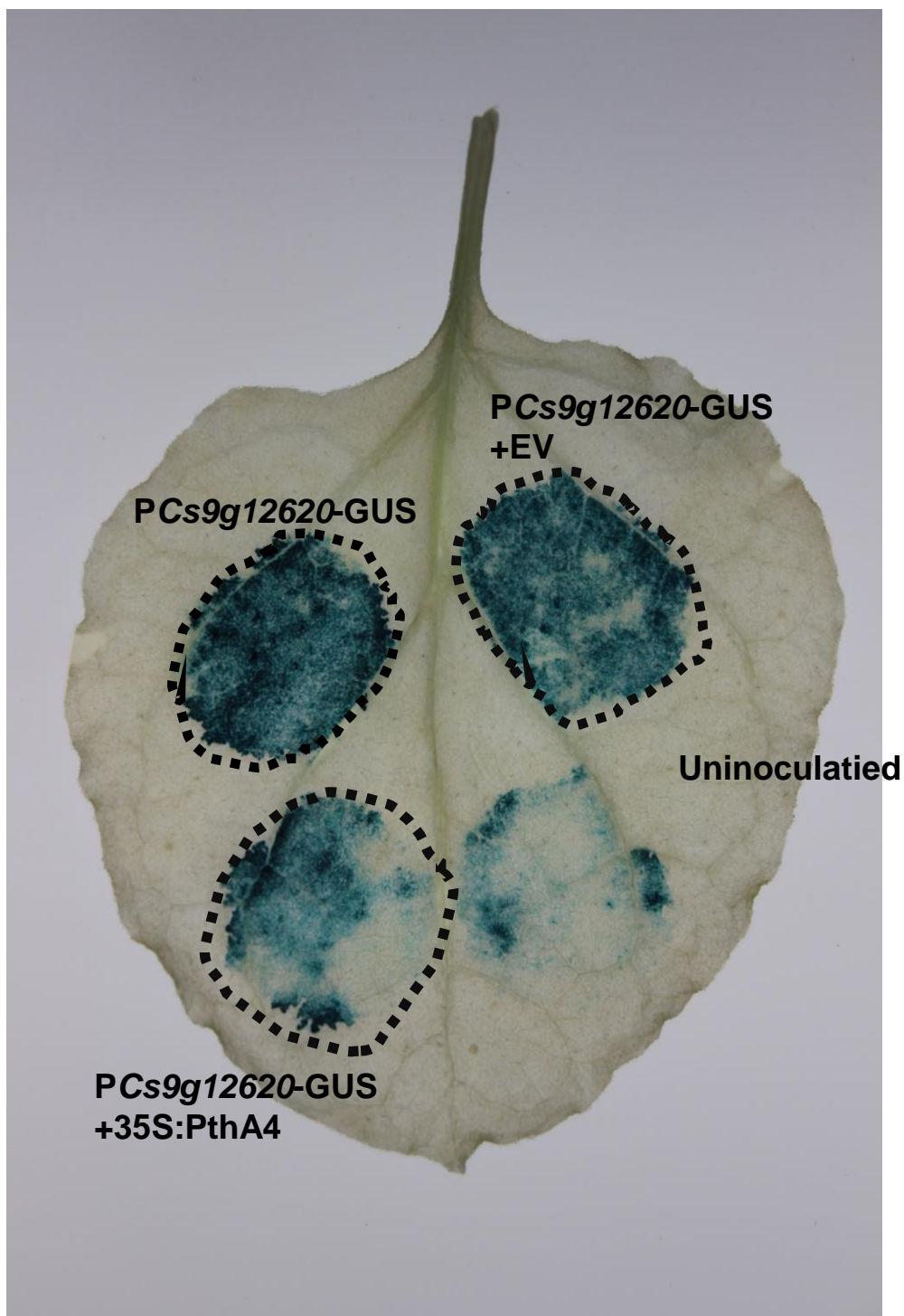**Figure 3**

Supplement: Figure 3—source data 8. [file elife-91684-fig3-data8.pdf]

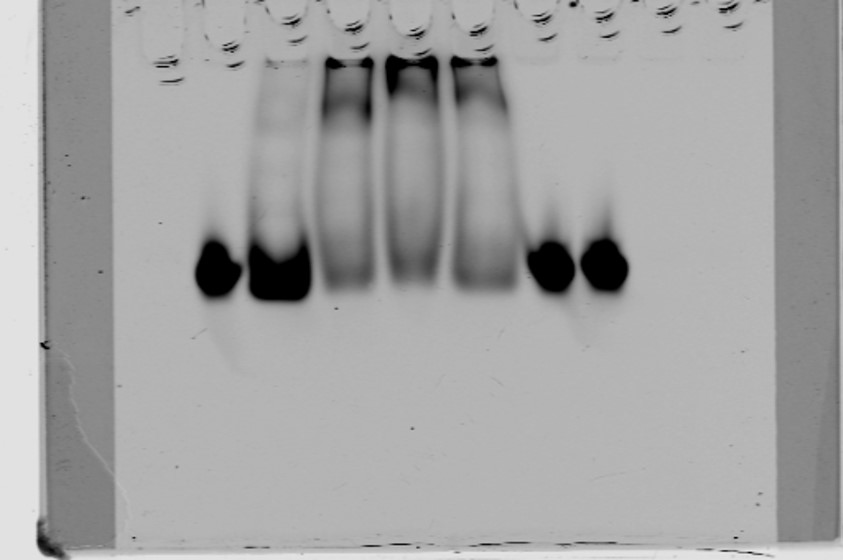

Supplement: Figure 3—source data 9. [file elife-91684-fig3-data9.zip › Figure 3- source data 9/Figure 3- source data 9.jpg]

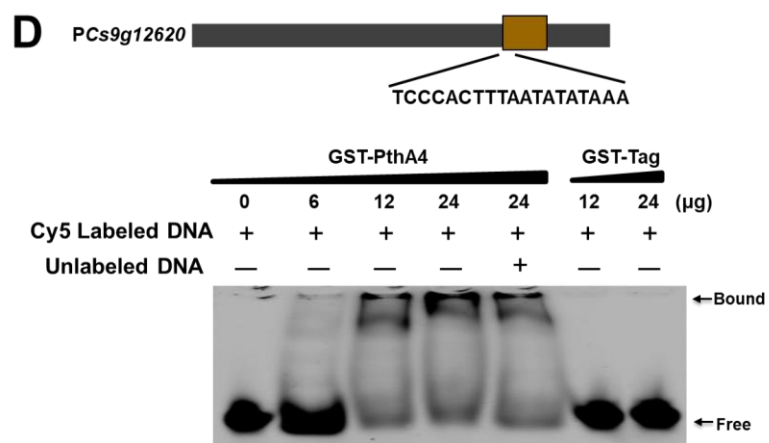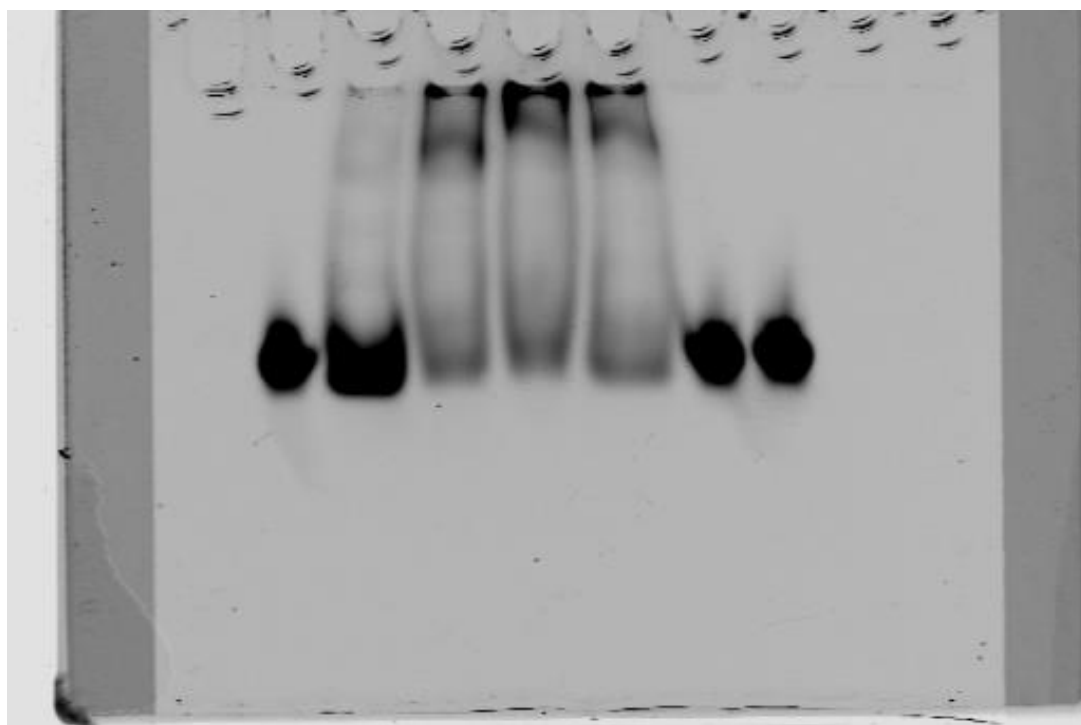

**Figure 3**

Supplement: Figure 3—source data 10. [file elife-91684-fig3-data10.pdf]

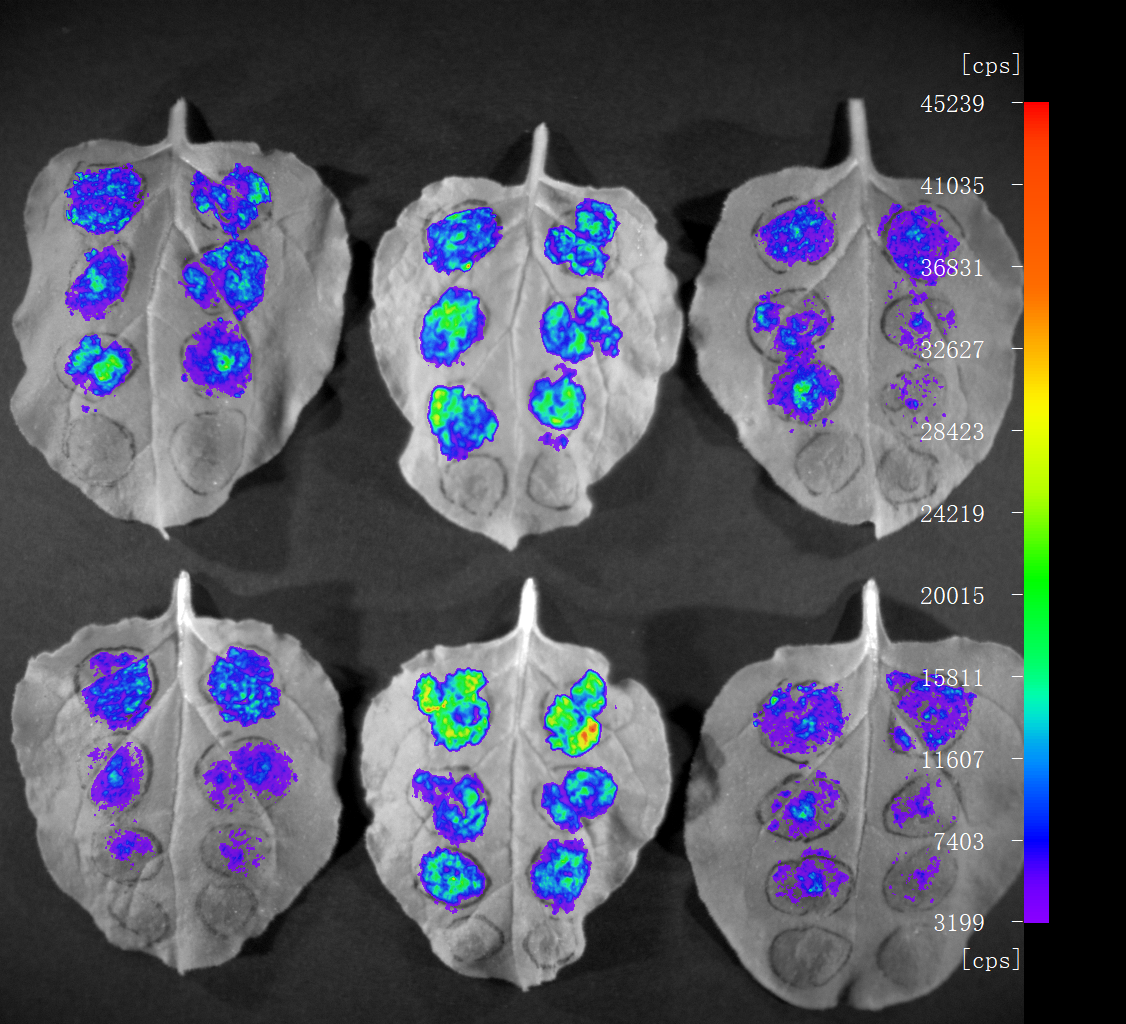

Supplement: Figure 3—source data 11. [file elife-91684-fig3-data11.zip › Figure 3- source data 11/Figure 3- source data 11.JPG]

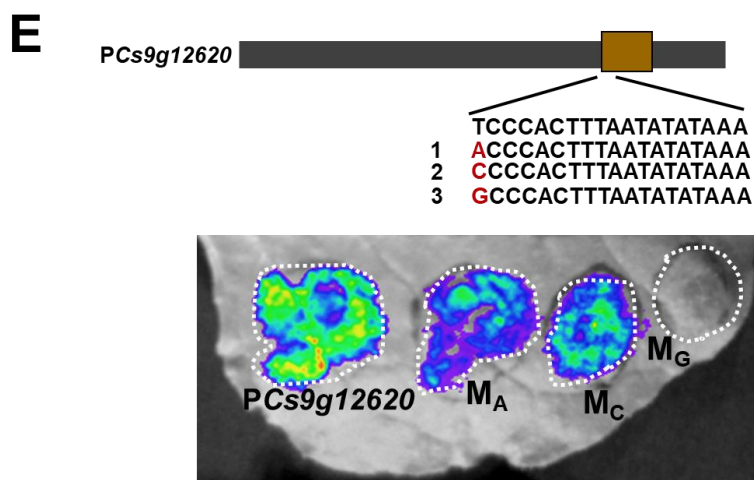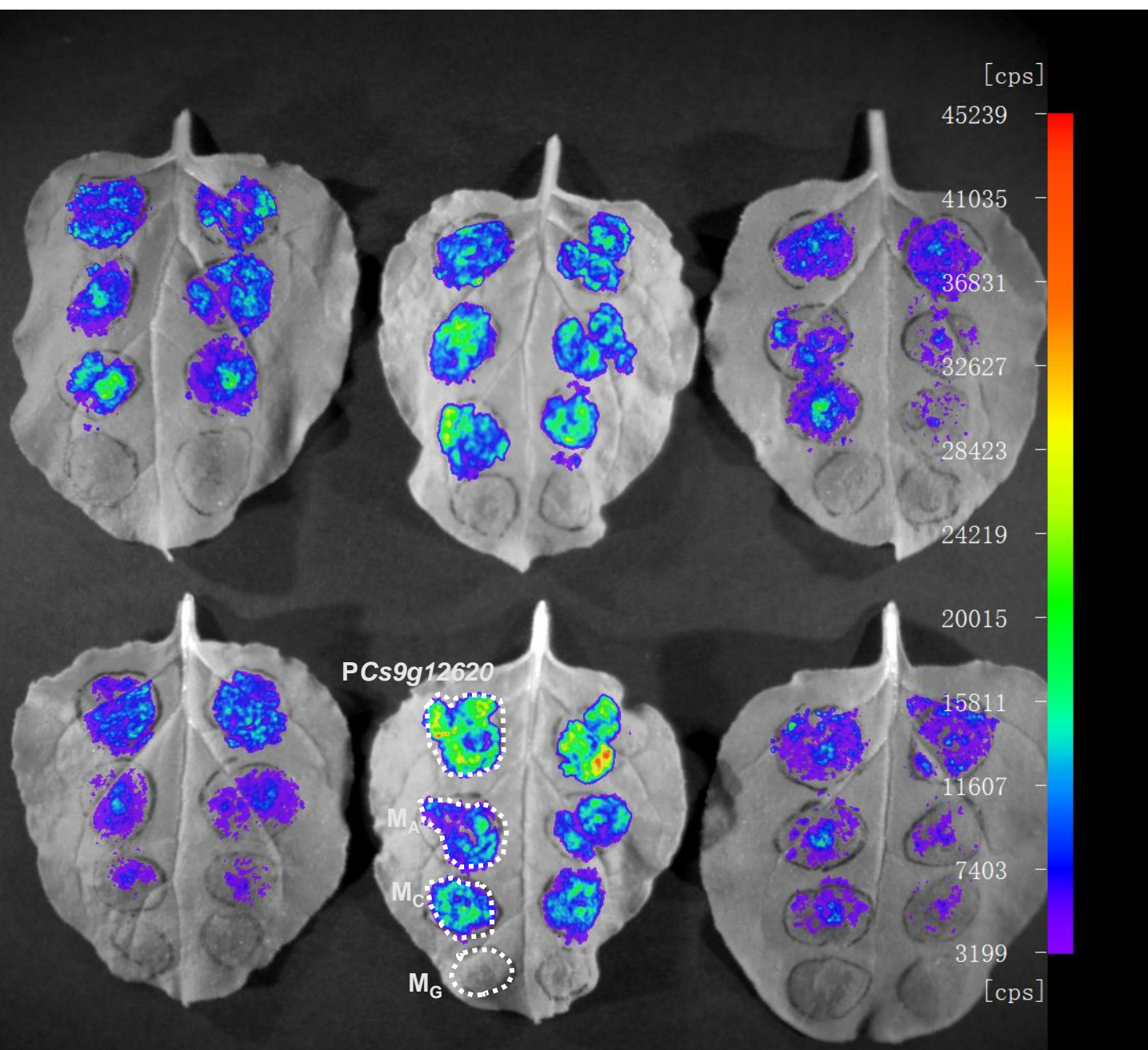

**Figure 3**

Supplement: Figure 3—source data 12. [file elife-91684-fig3-data12.pdf]

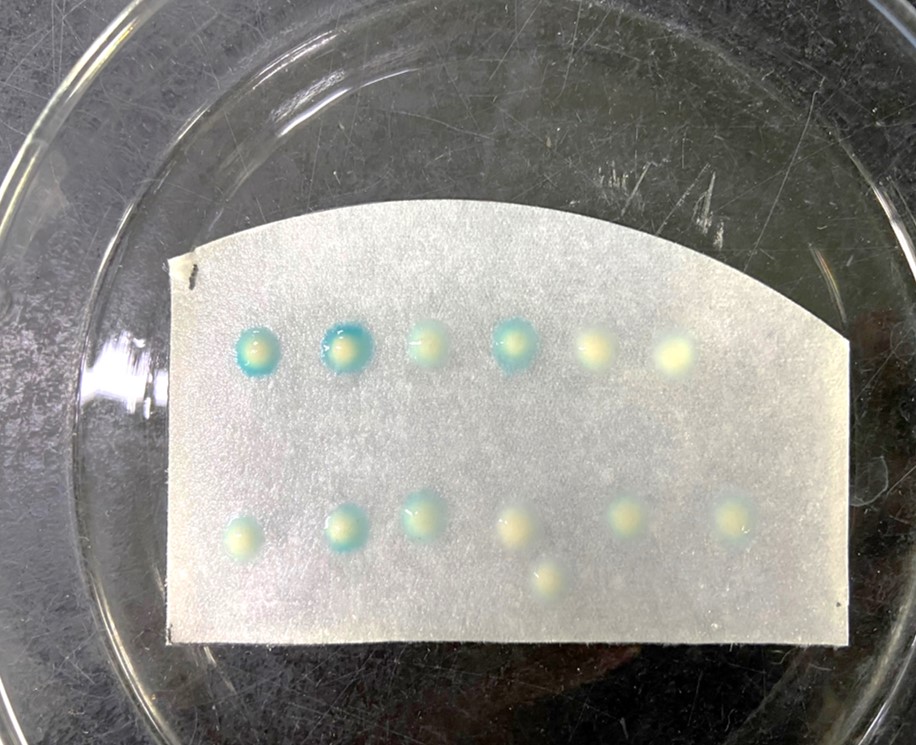

Supplement: Figure 3—source data 13. [file elife-91684-fig3-data13.zip › Figure 3- source data 13/Figure 3- source data 13.jpg]

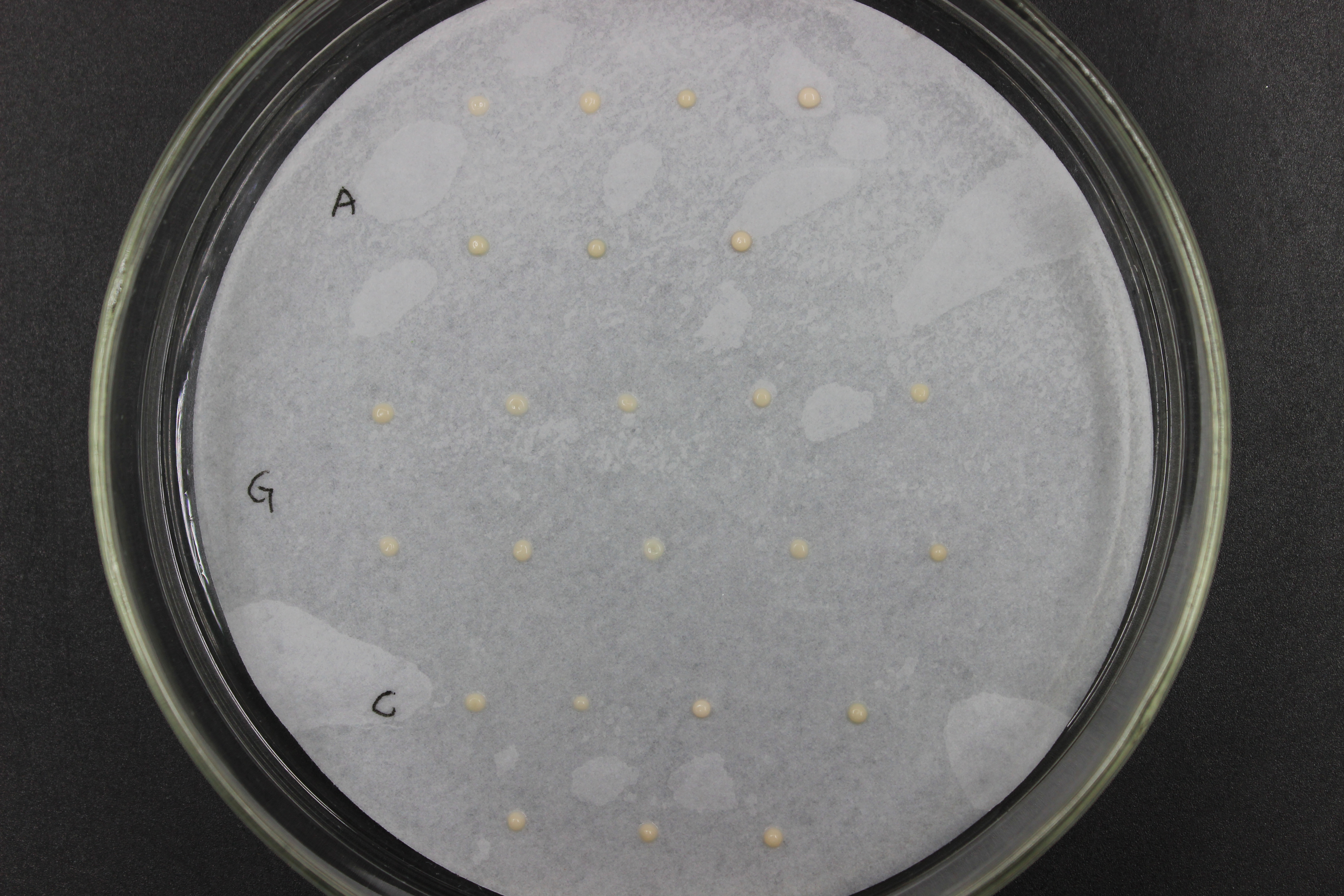

Supplement: Figure 3—source data 14. [file elife-91684-fig3-data14.zip › Figure 3- source data 14/Figure 3- source data 14.JPG]

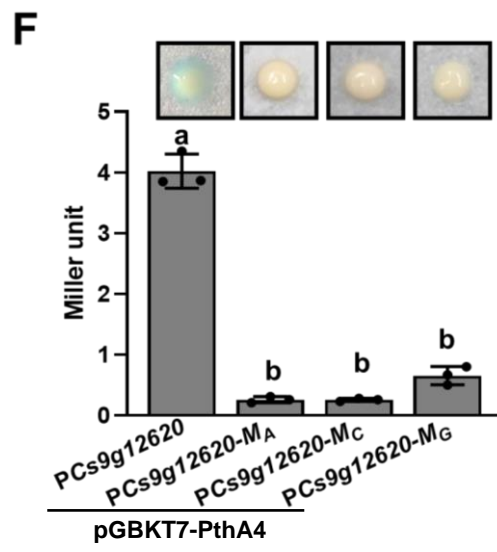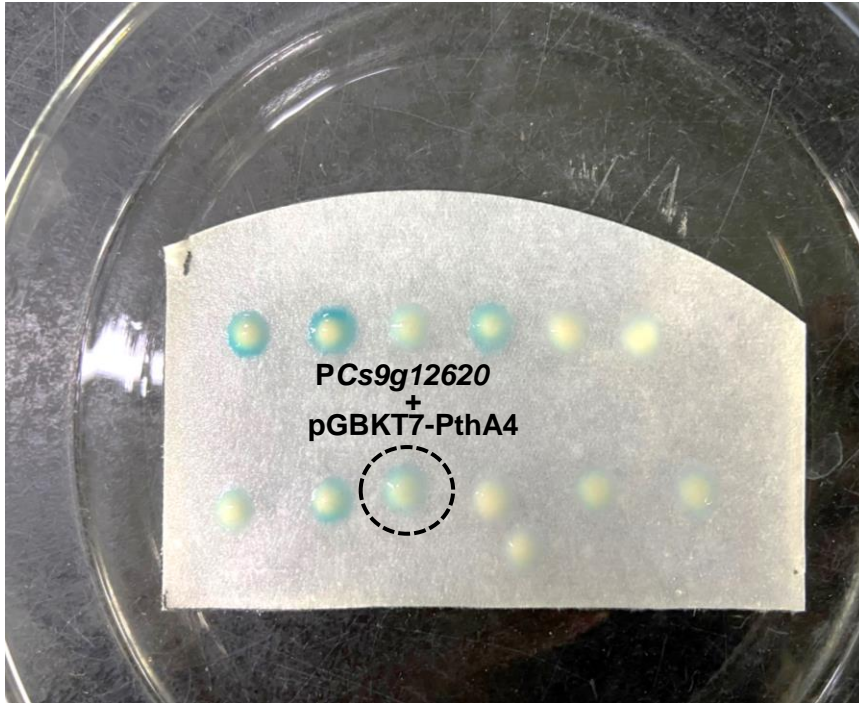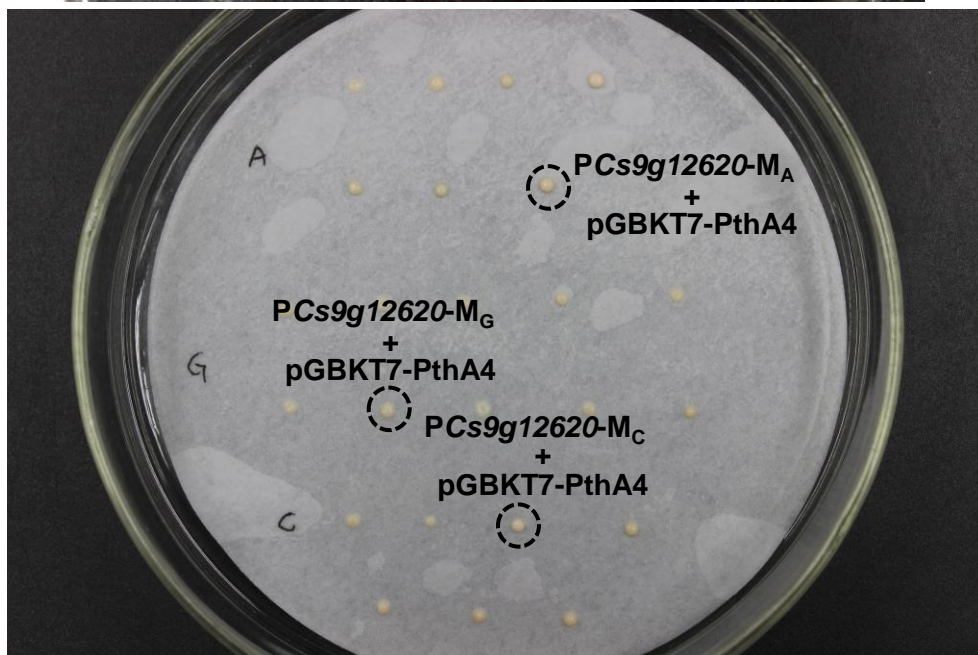

**Figure 3**

Supplement: Figure 3—source data 15. [file elife-91684-fig3-data15.pdf]

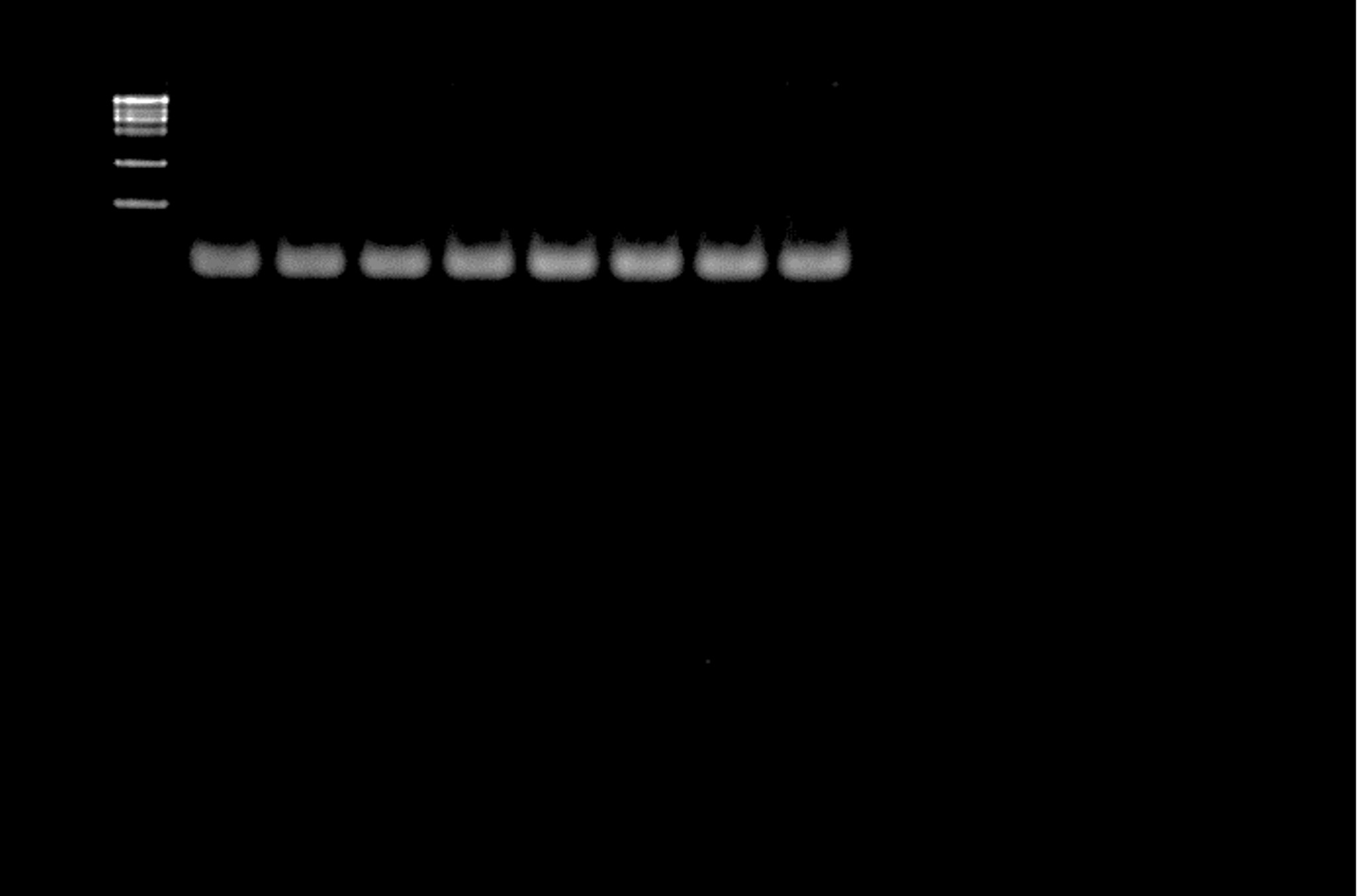

Supplement: Figure 3—source data 16. [file elife-91684-fig3-data16.zip › Figure 3- source data 16/Figure 3- source data 16.JPG]

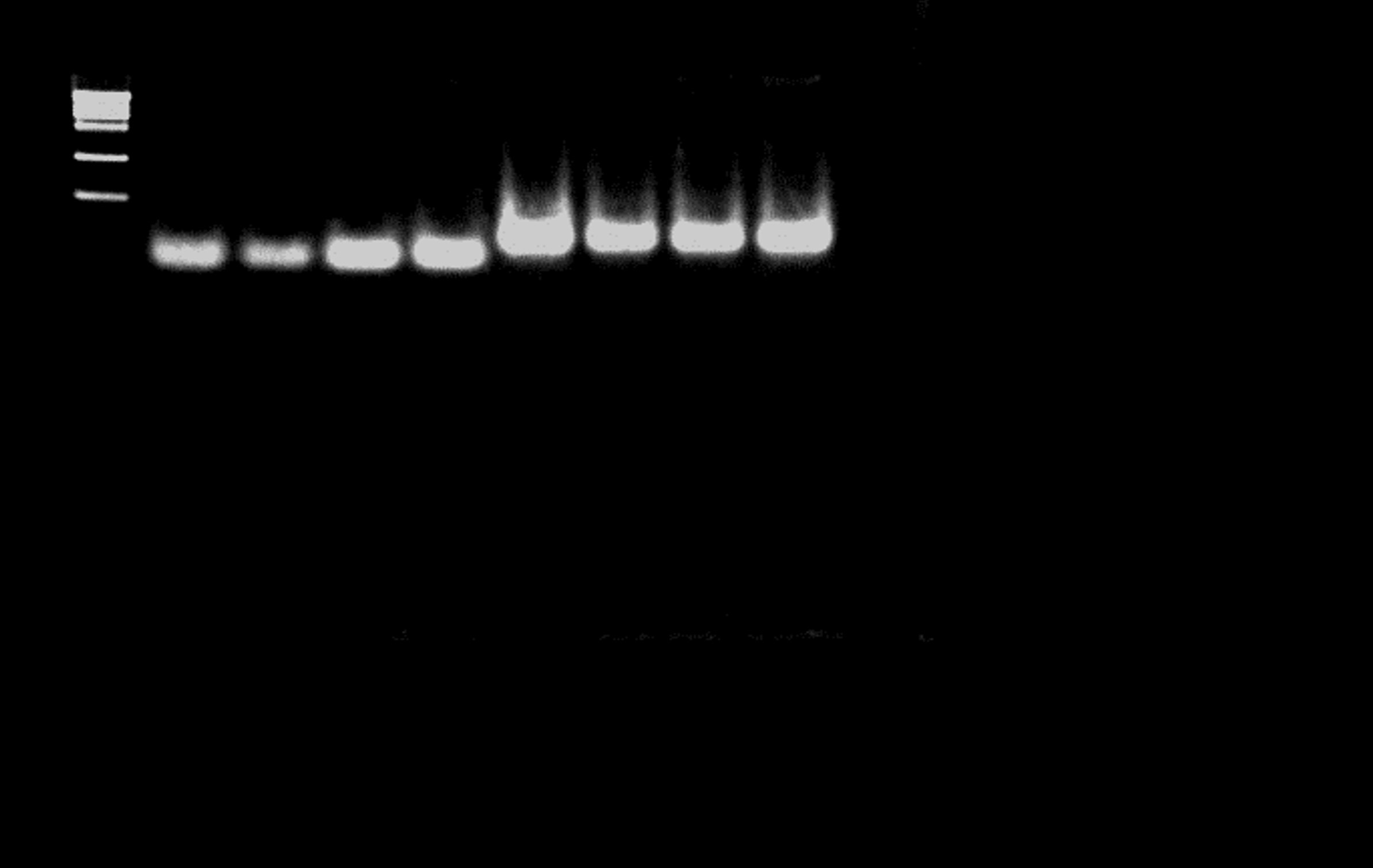

Supplement: Figure 3—source data 17. [file elife-91684-fig3-data17.zip › Figure 3- source data 17/Figure 3- source data 17.JPG]

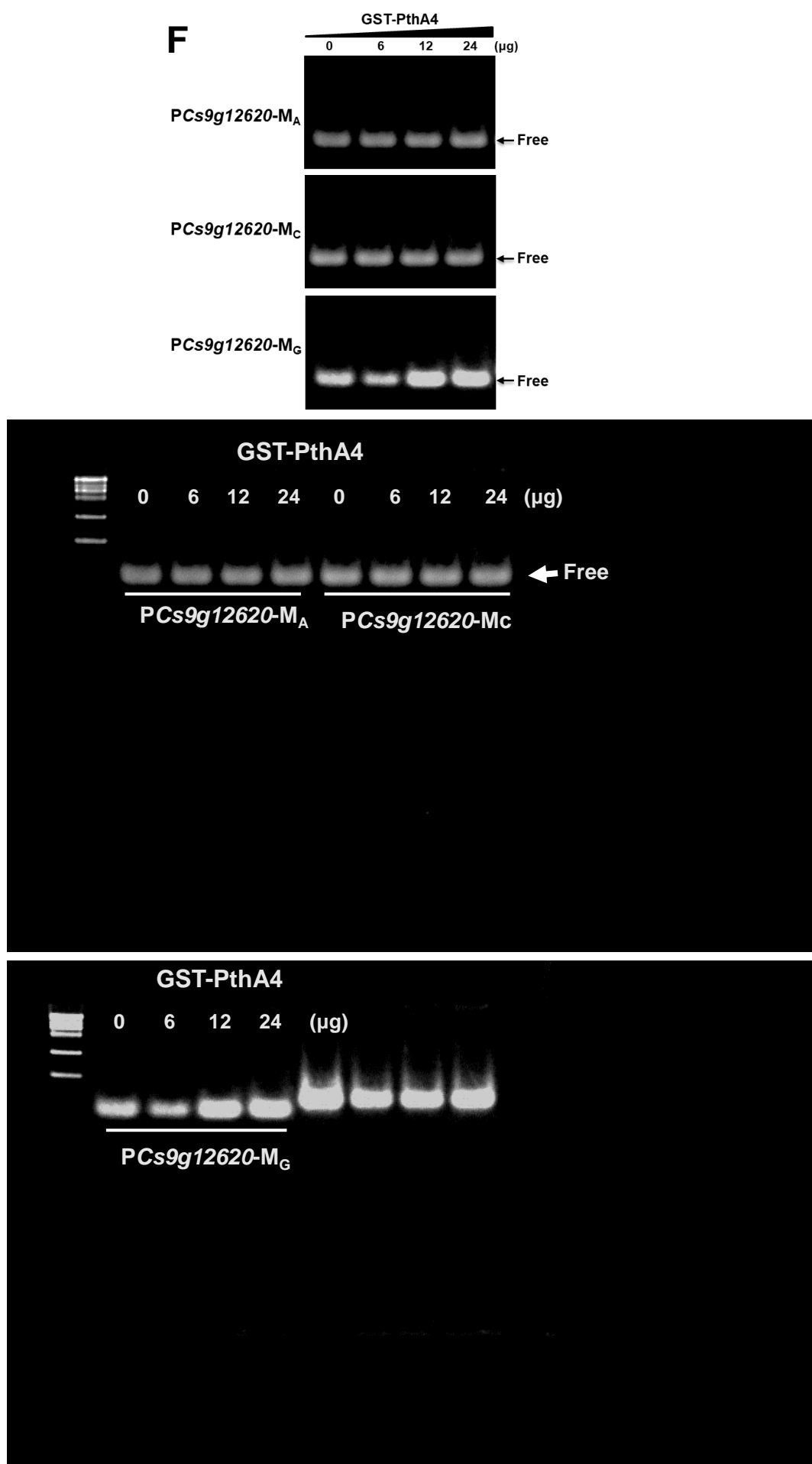

**Figure 3**

Supplement: Figure 3—source data 18. [file elife-91684-fig3-data18.pdf]

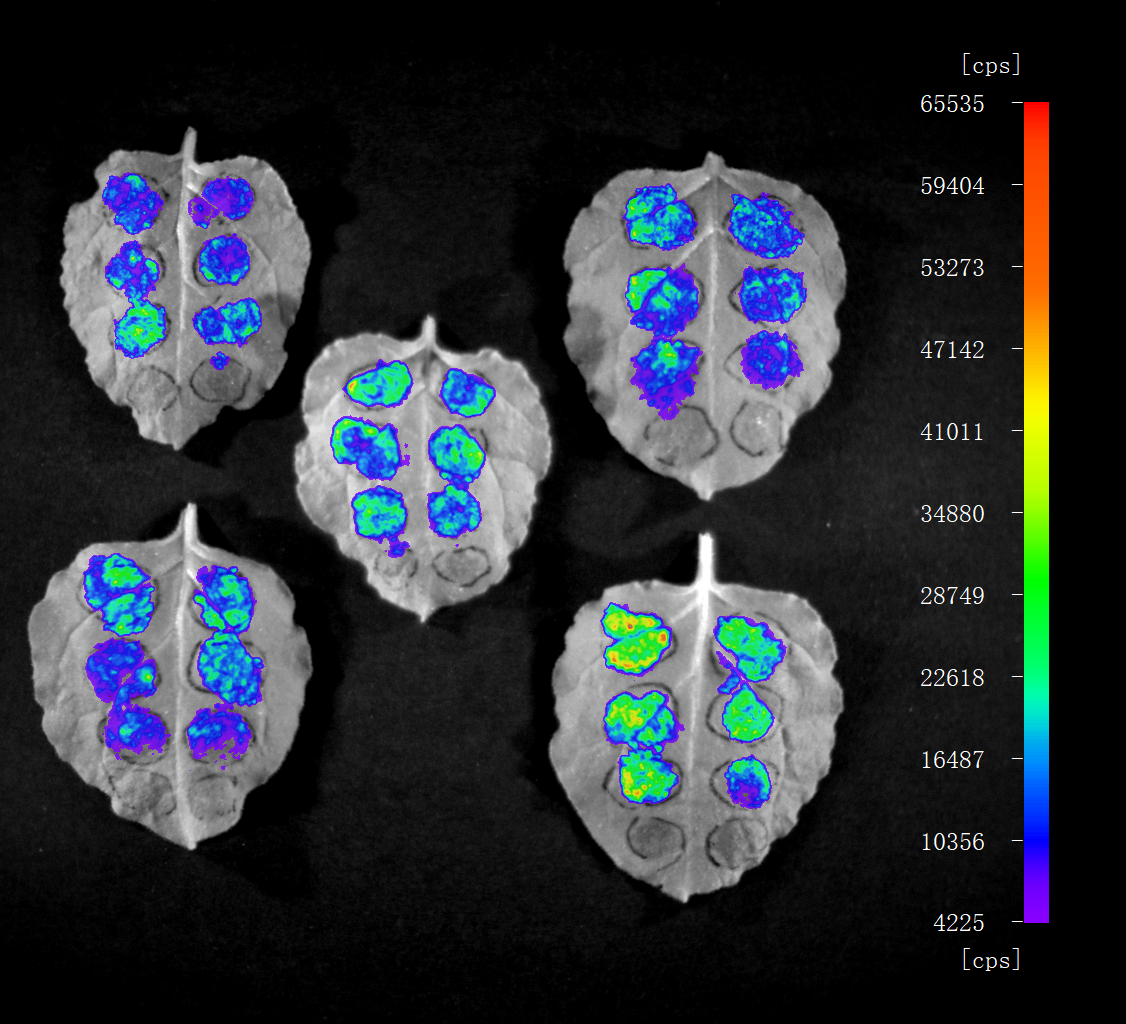

Supplement: Figure 3—source data 19. [file elife-91684-fig3-data19.zip › Figure 3- source data 19/Figure 3- source data 19.JPG]

**G**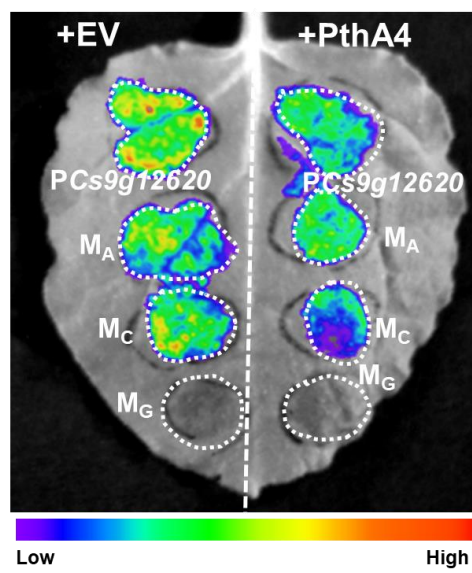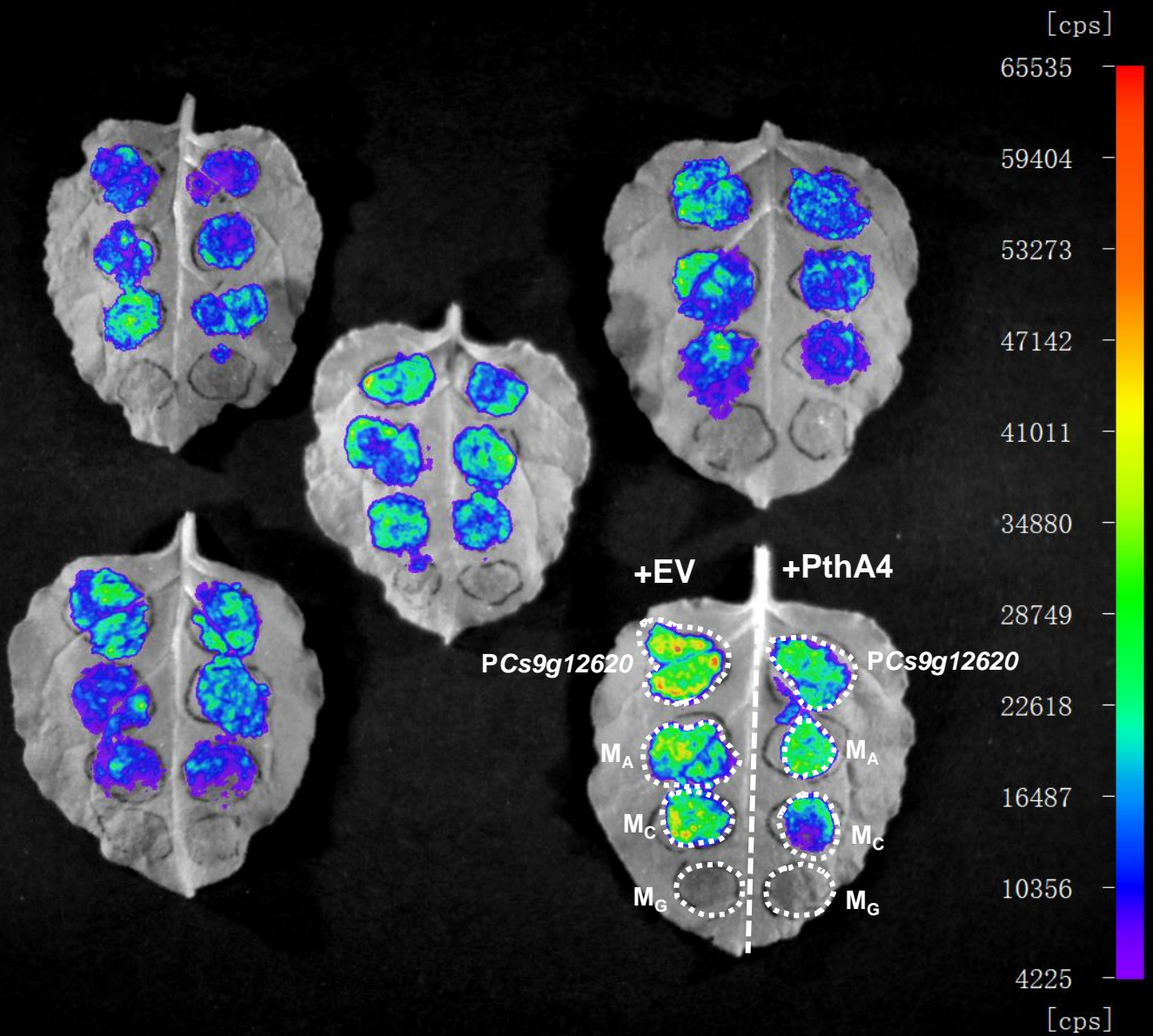

**Figure 3**

Supplement: Figure 3—source data 20. [file elife-91684-fig3-data20.pdf]

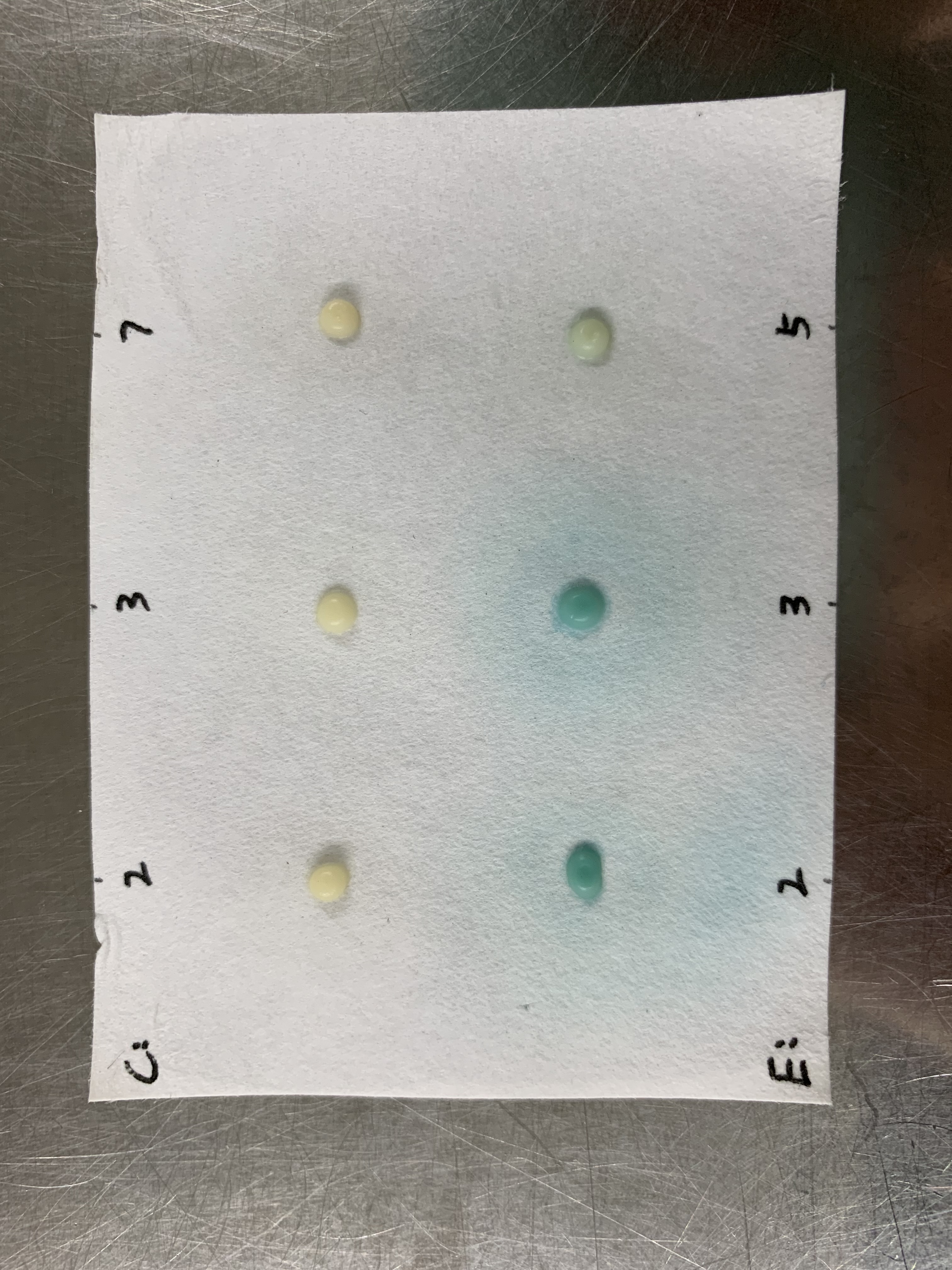

Supplement: Figure 4—source data 2. [file elife-91684-fig4-data2.zip › Figure 4- source data 2/Figure 4- source data 2.JPG]

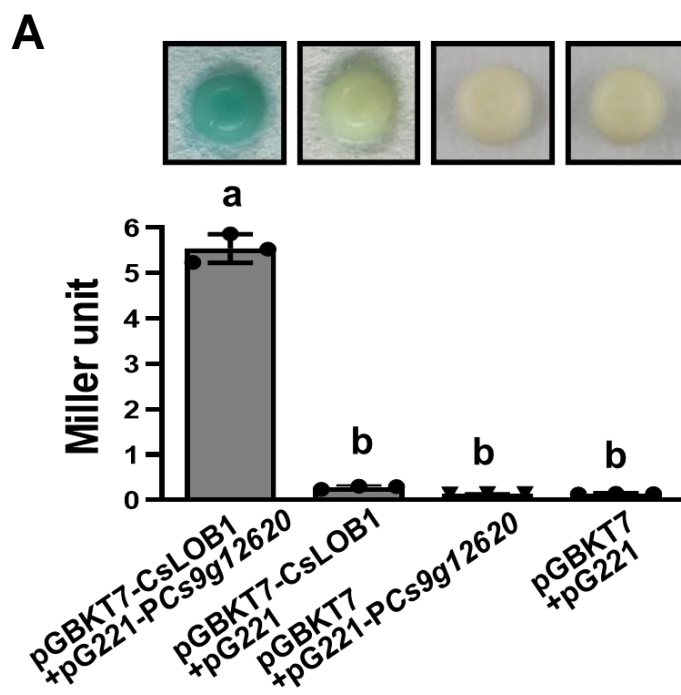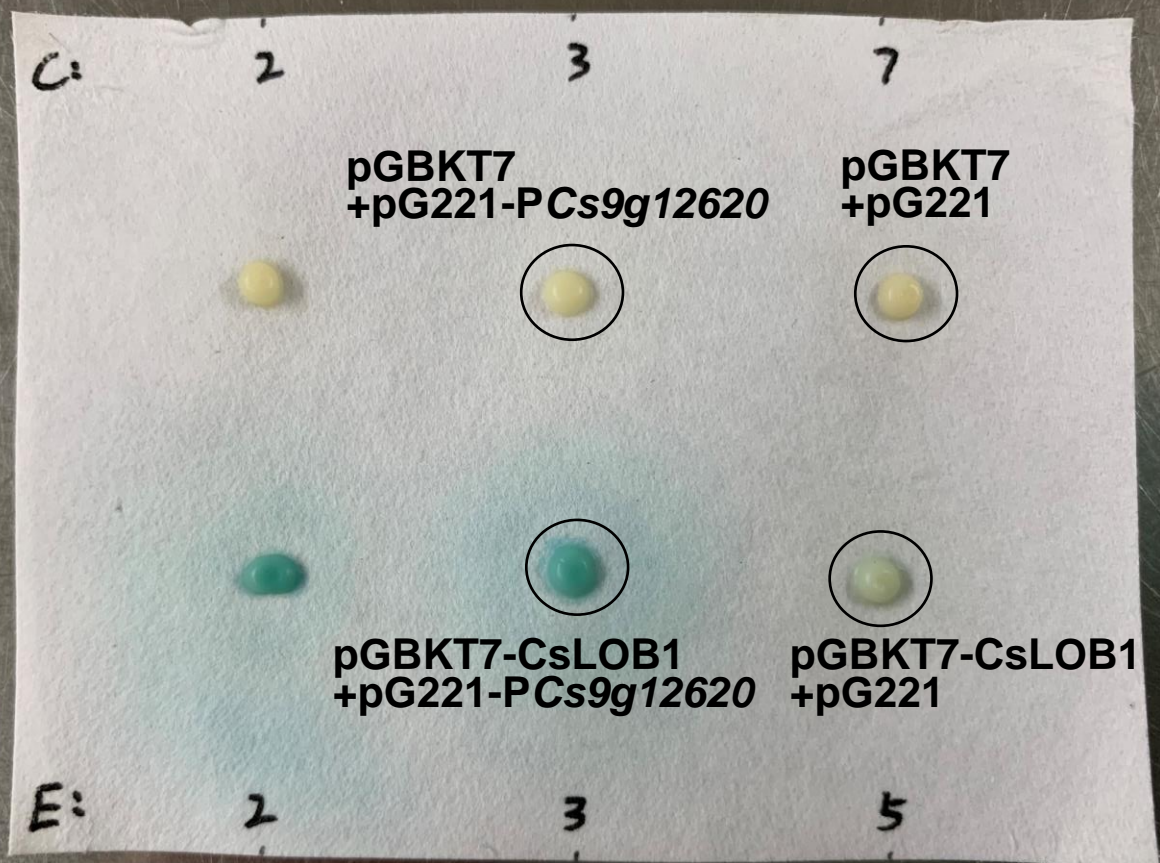

**Figure 4**

Supplement: Figure 4—source data 3. [file elife-91684-fig4-data3.pdf]

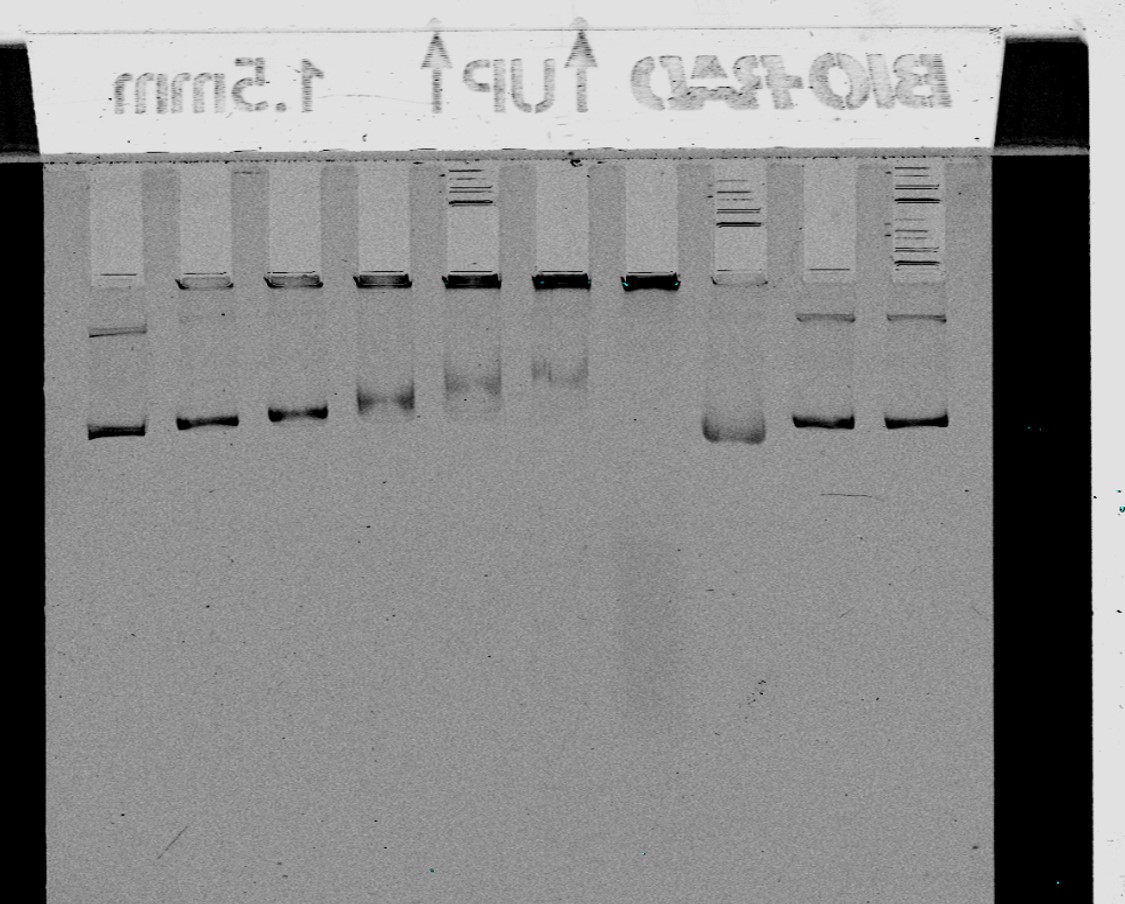

Supplement: Figure 4—source data 4. [file elife-91684-fig4-data4.zip › Figure 4- source data 4/Figure 4- source data 4.jpg]

**B**

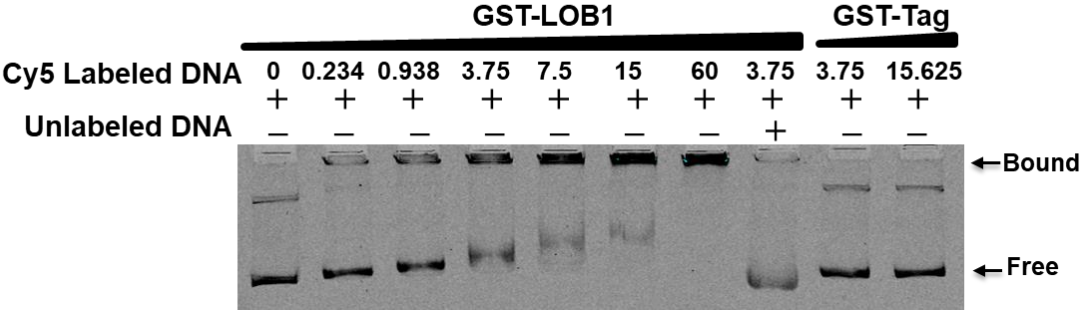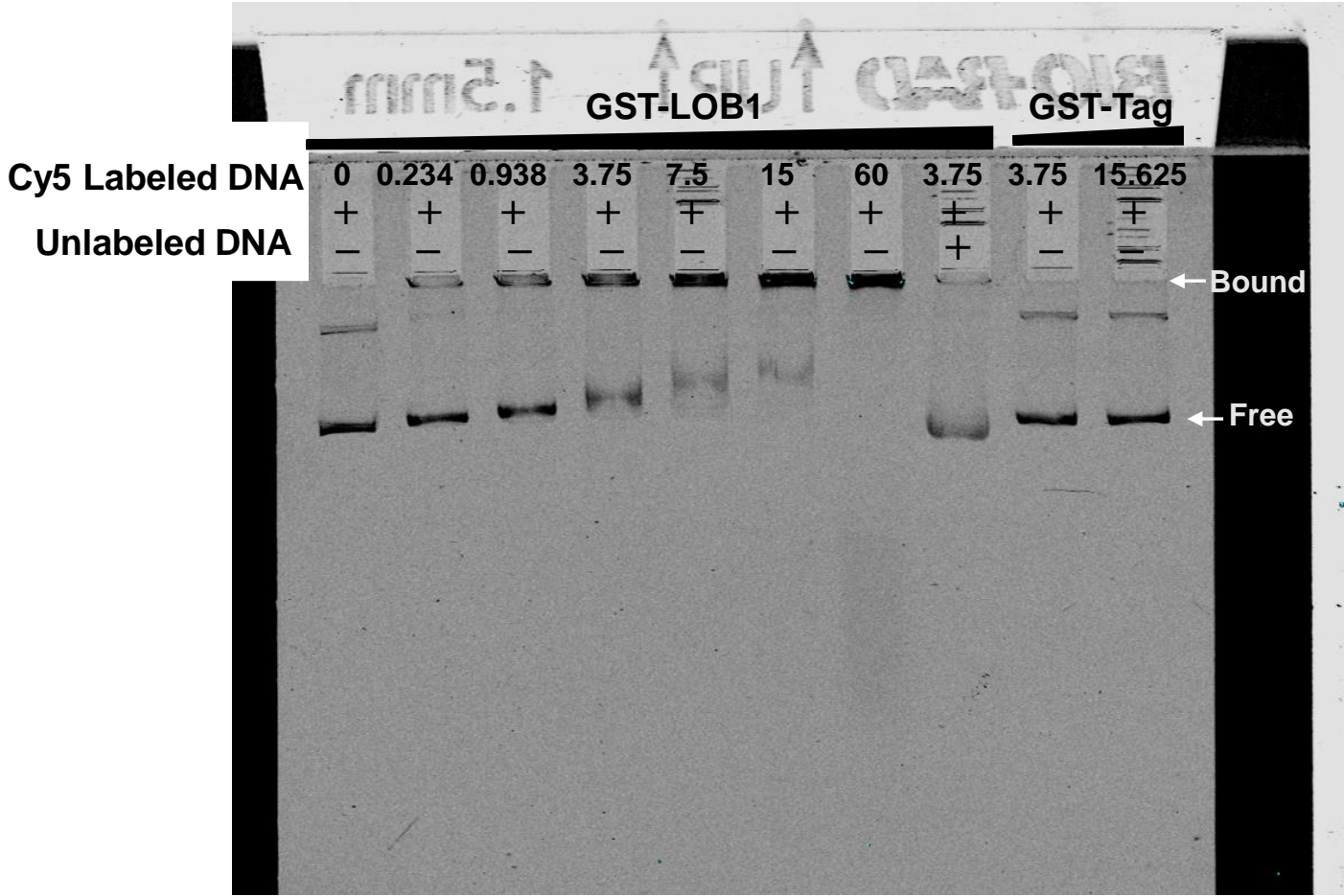

**Figure 4**

Supplement: Figure 4—source data 5. [file elife-91684-fig4-data5.pdf]

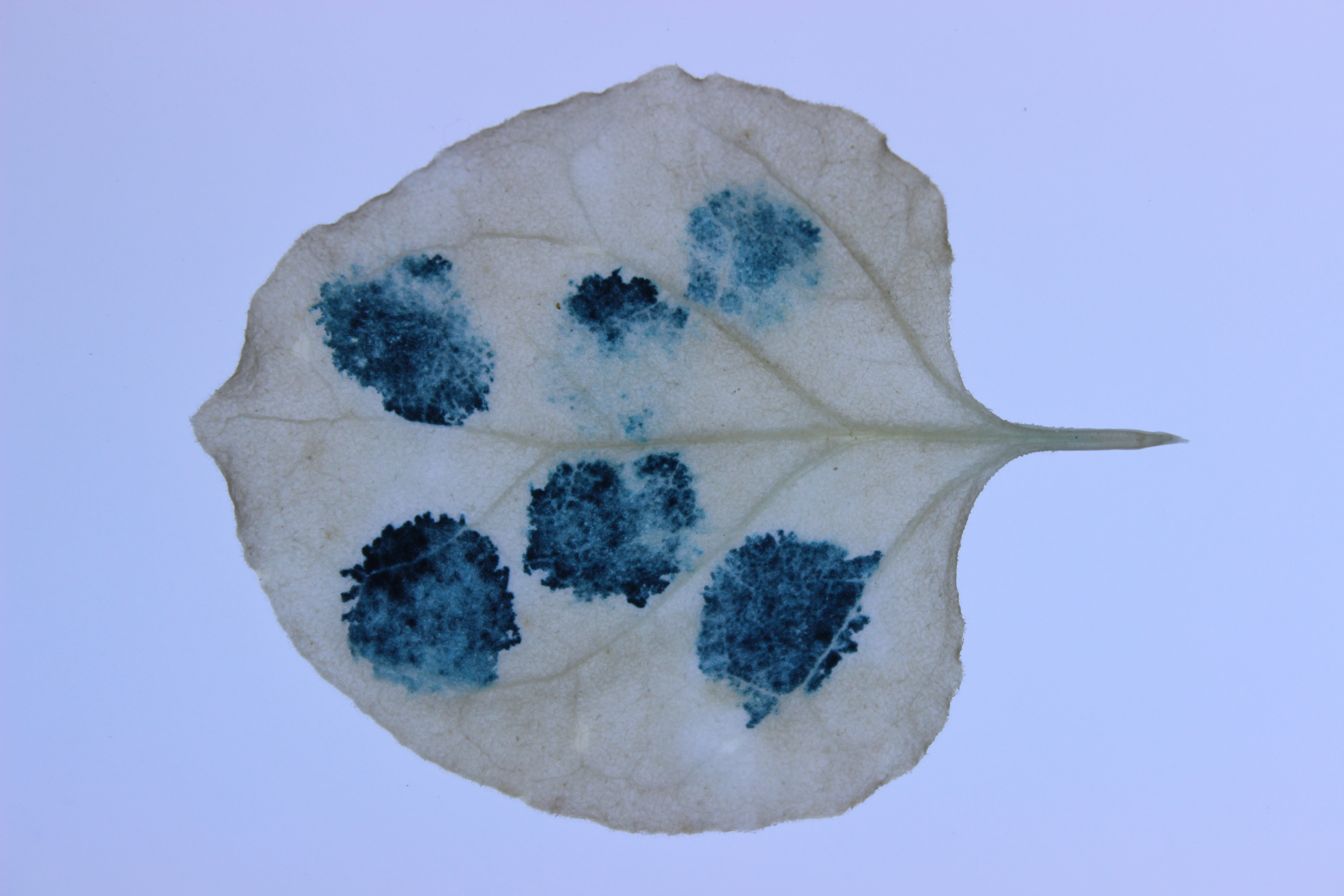

Supplement: Figure 4—source data 6. [file elife-91684-fig4-data6.zip › Figure 4- source data 6/Figure 4- source data 6.JPG]

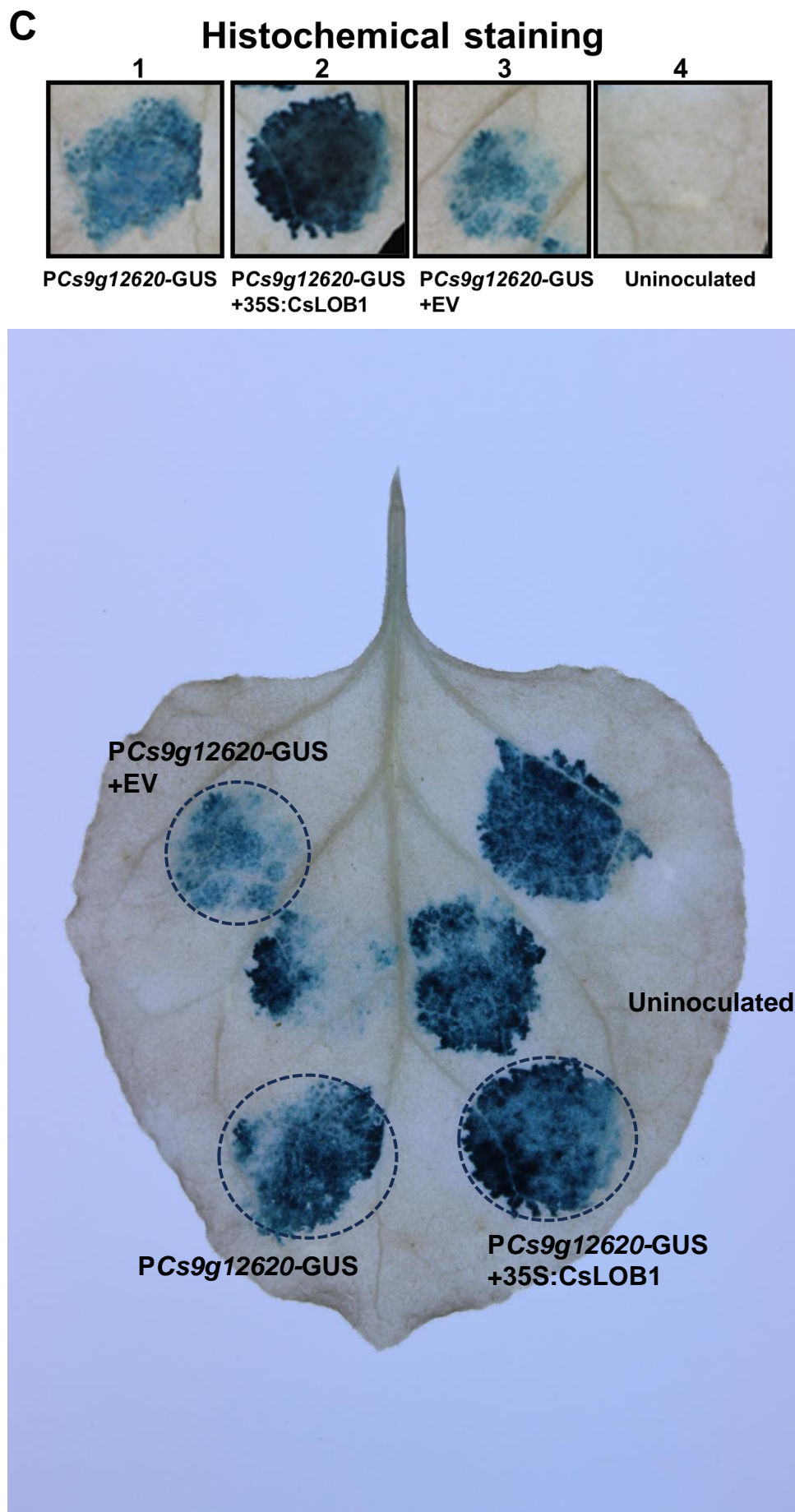

**Figure 4**

Supplement: Figure 4—source data 7. [file elife-91684-fig4-data7.pdf]

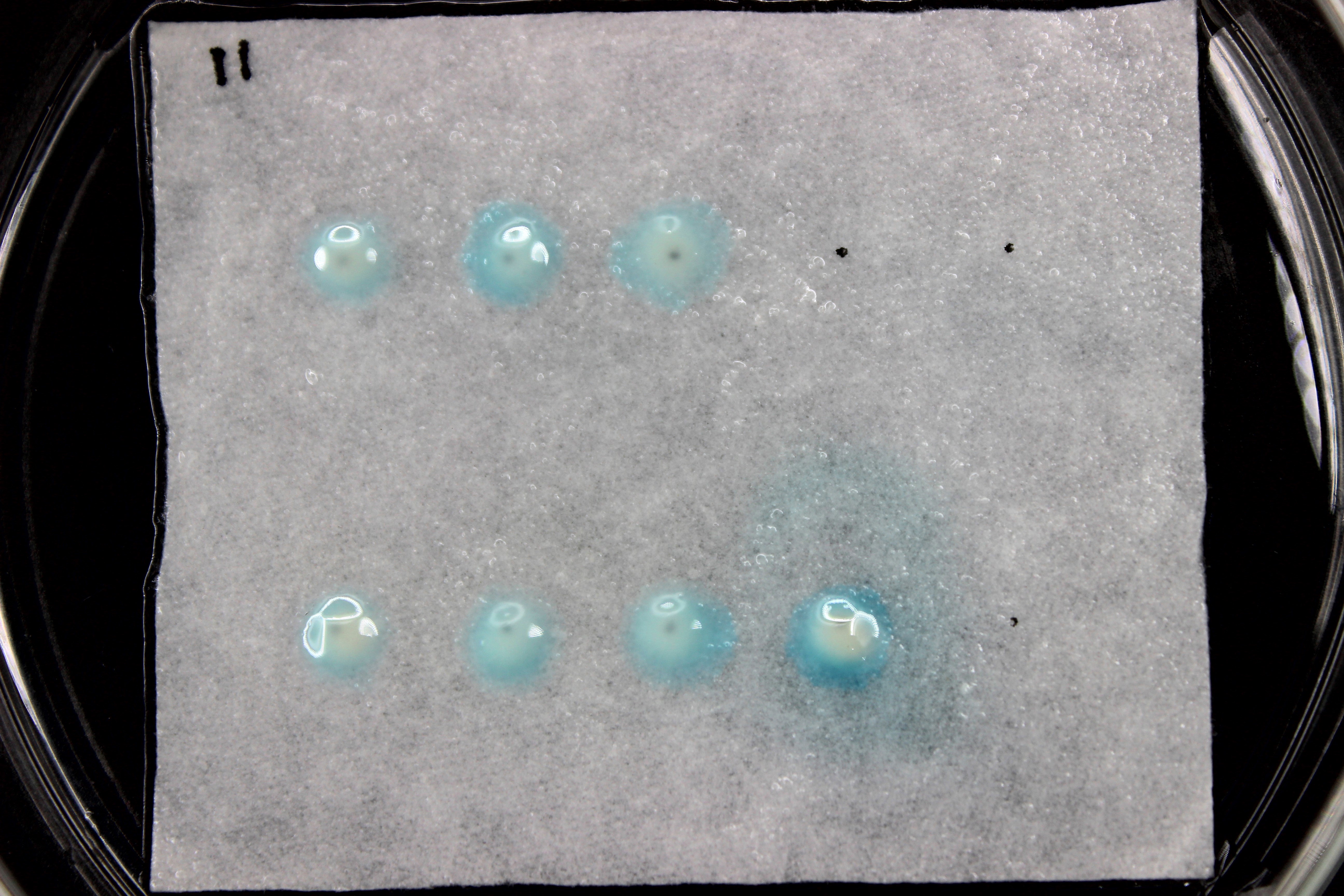

Supplement: Figure 4—source data 8. [file elife-91684-fig4-data8.zip › Figure 4- source data 8/Figure 4- source data 8.jpg]

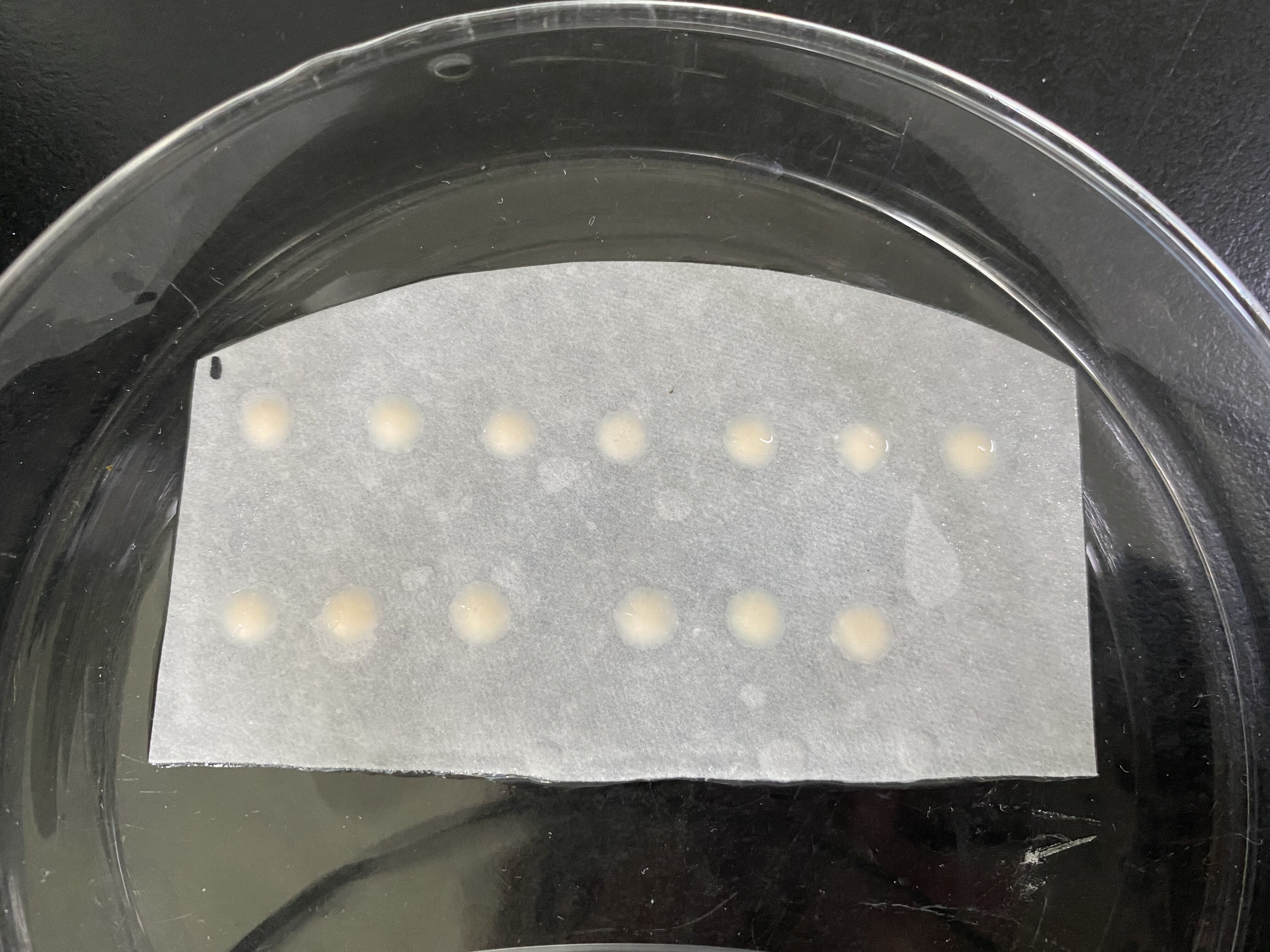

Supplement: Figure 4—source data 9. [file elife-91684-fig4-data9.zip › Figure 4- source data 9/Figure 4- source data 9.jpg]

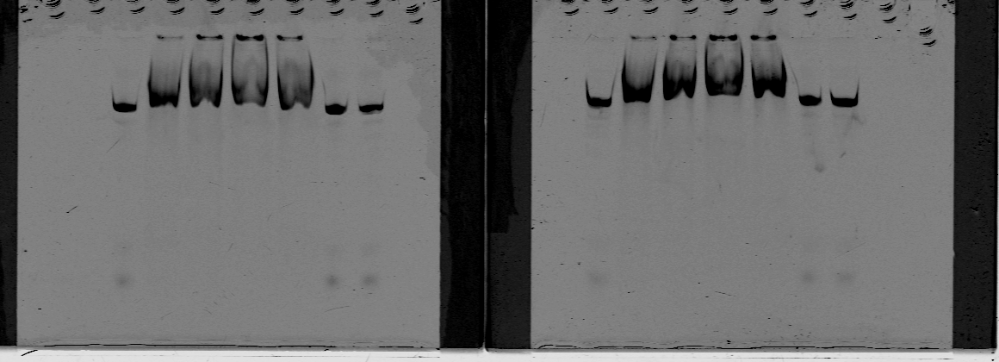

Supplement: Figure 4—source data 11. [file elife-91684-fig4-data11.zip › Figure 4- source data 11/Figure 4- source data 11.jpg]

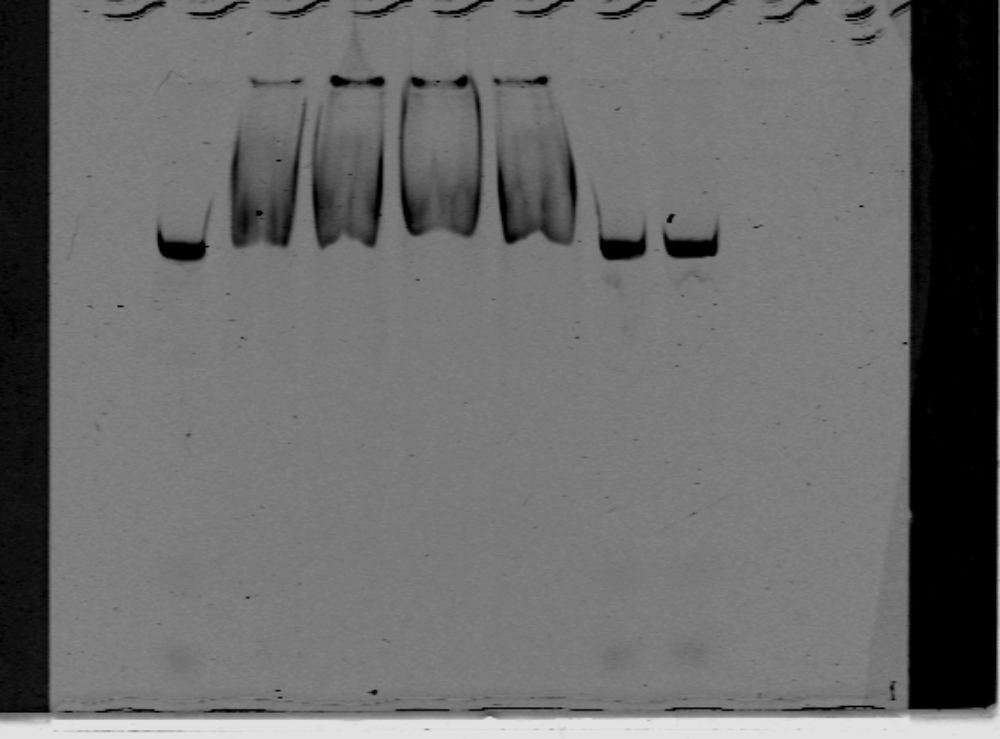

Supplement: Figure 4—source data 12. [file elife-91684-fig4-data12.zip › Figure 4- source data 12/Figure 4- source data 12.jpg]

**E**

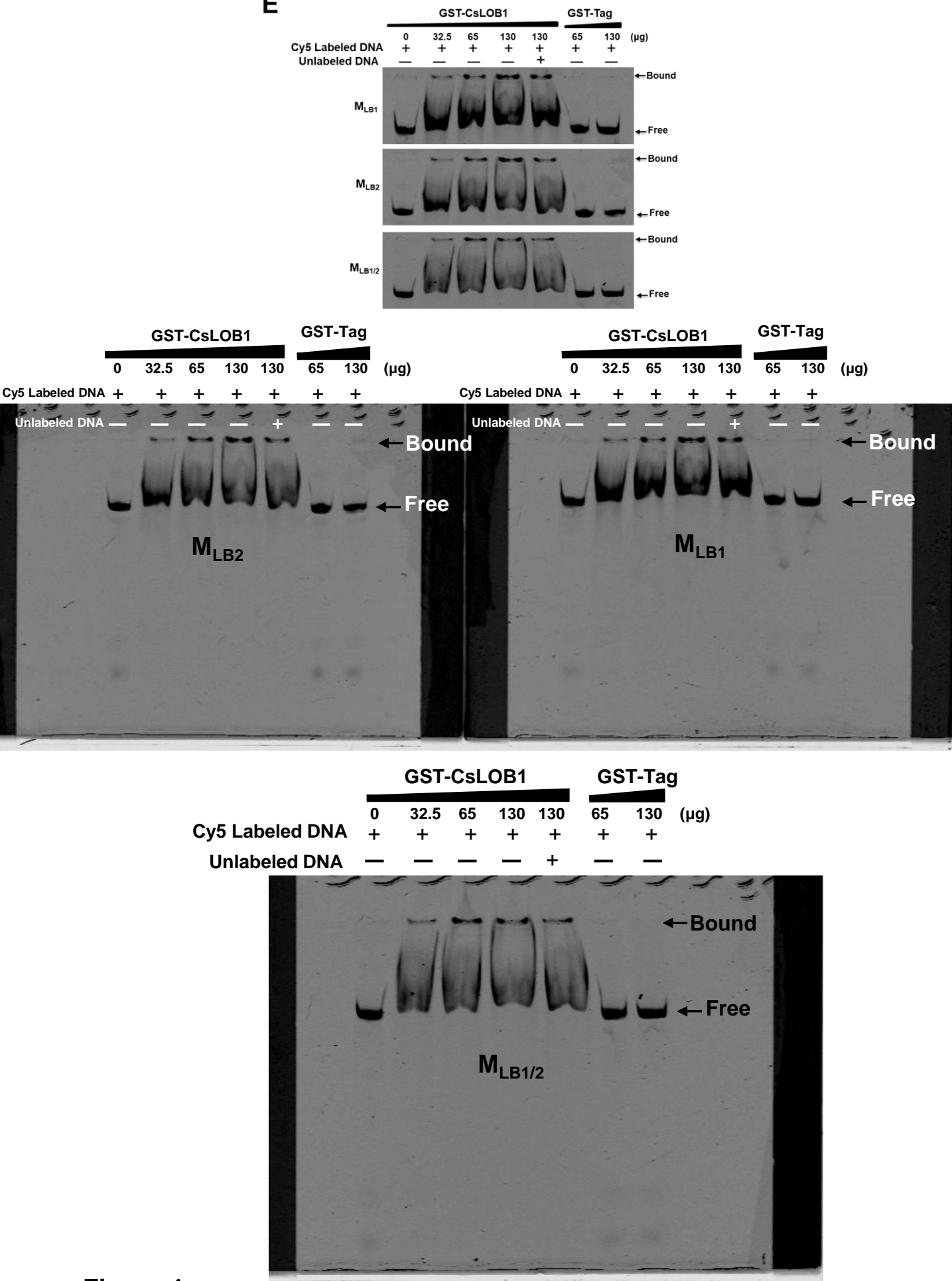

**Figure 4**

Supplement: Figure 4—source data 13. [file elife-91684-fig4-data13.pdf]

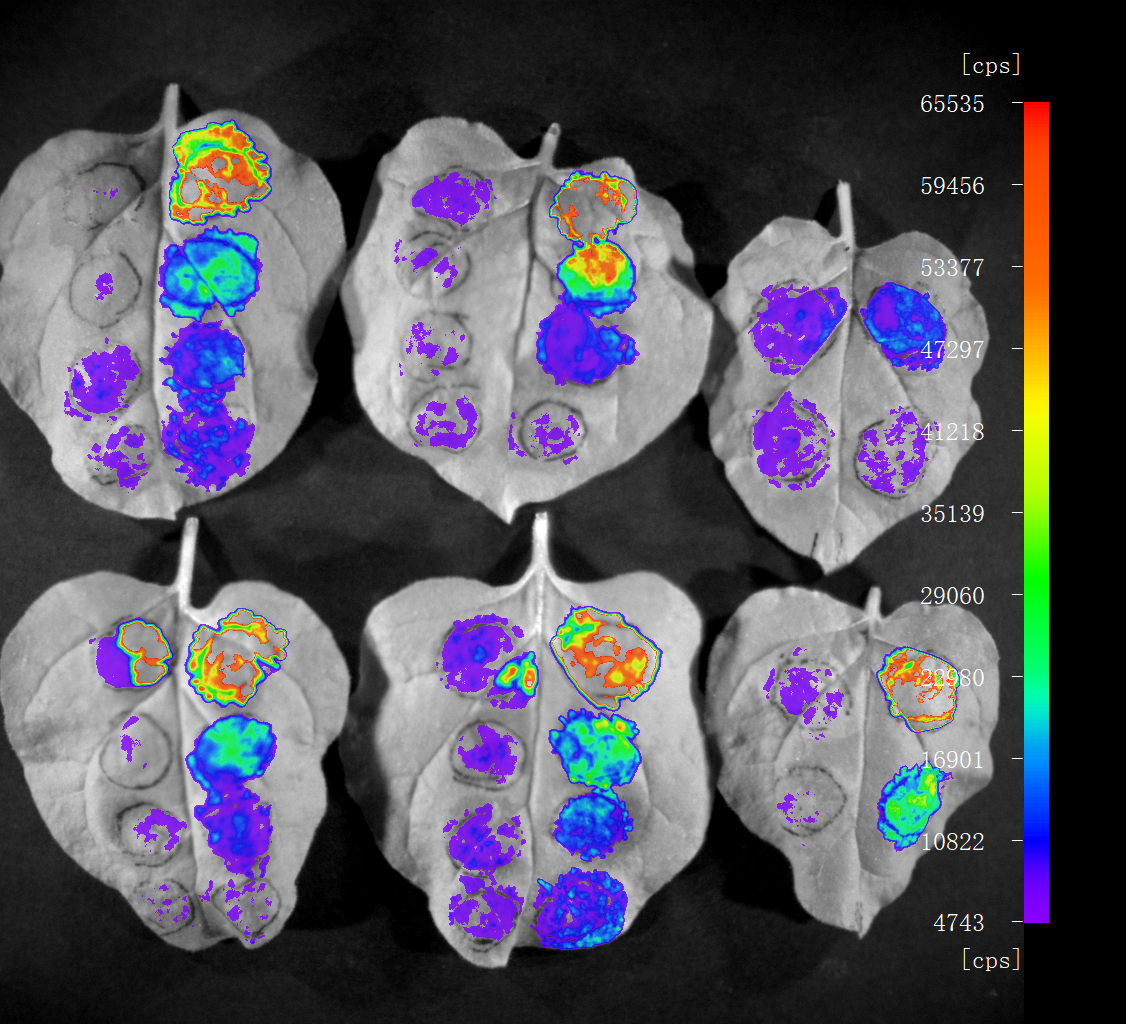

Supplement: Figure 4—source data 14. [file elife-91684-fig4-data14.zip › Figure 4- source data 14/Figure 4- source data 14.jpg]

**F**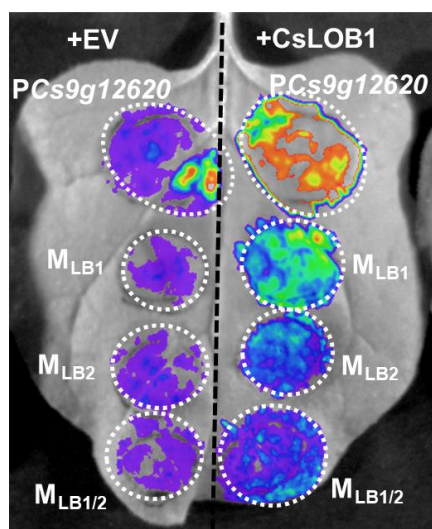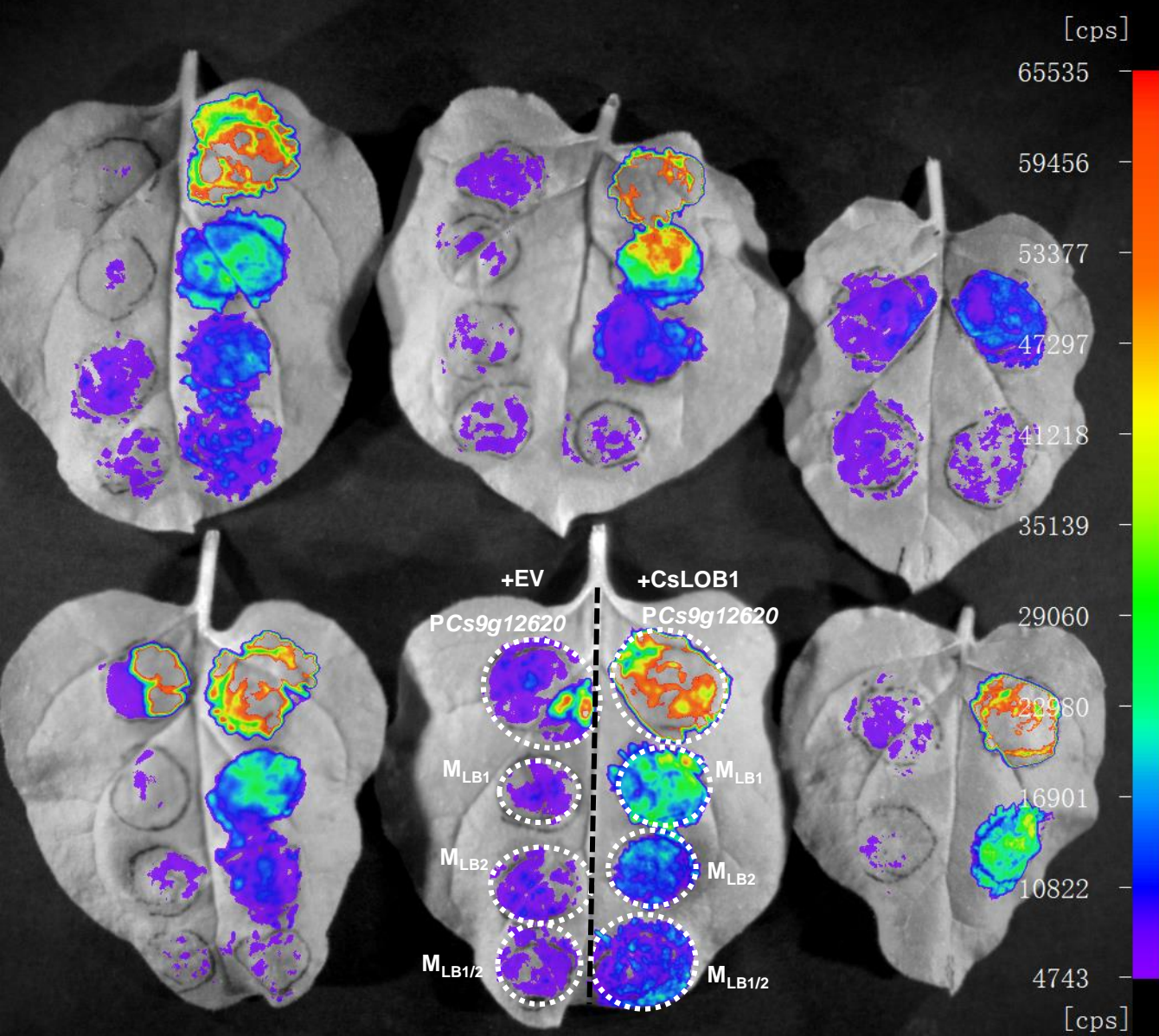

**Figure 4**

Supplement: Figure 4—source data 15. [file elife-91684-fig4-data15.pdf]

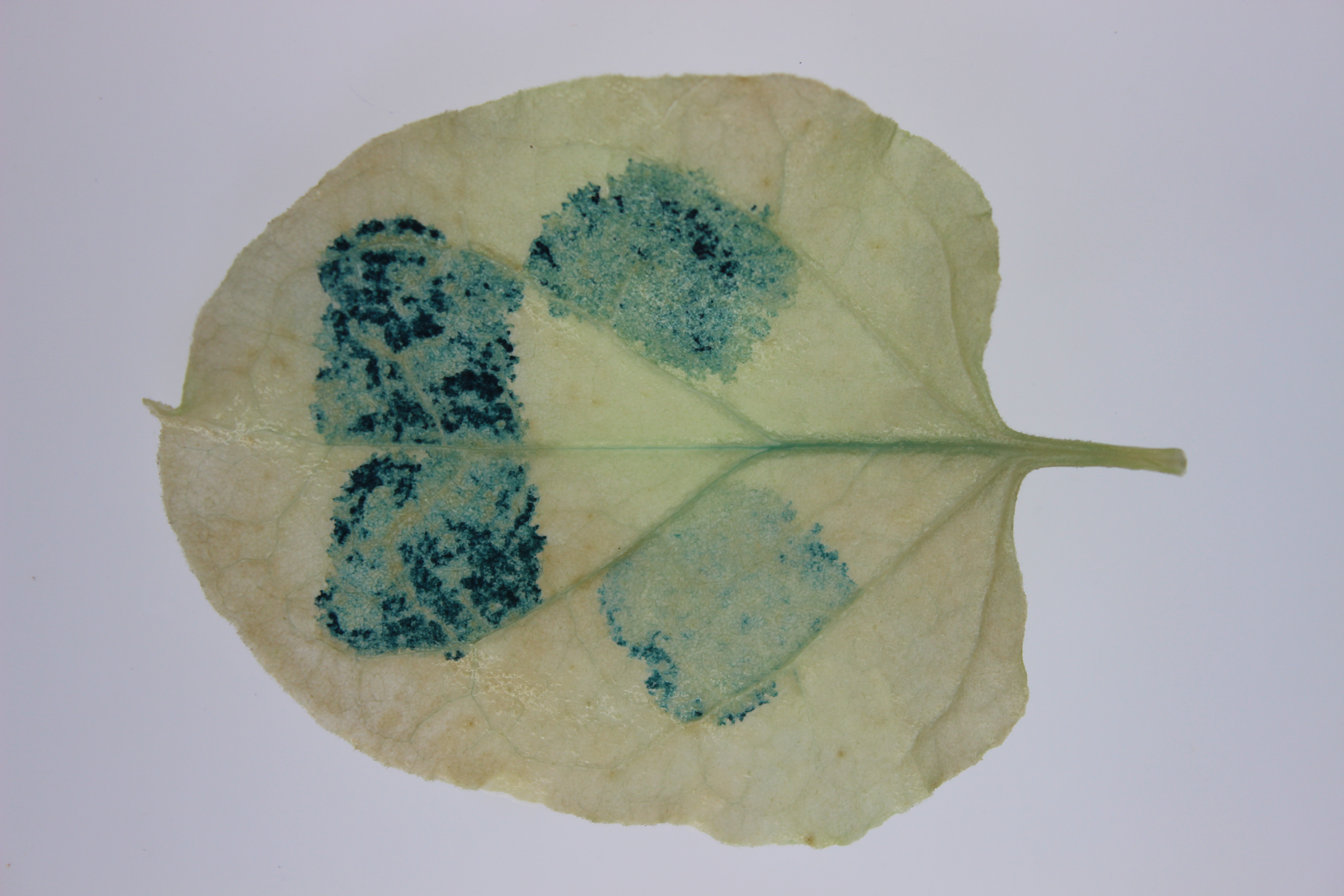

Supplement: Figure 5—source data 2. [file elife-91684-fig5-data2.zip › Figure 5- source data 2/Figure 5- source data 2.JPG]

**A****Histochemical staining**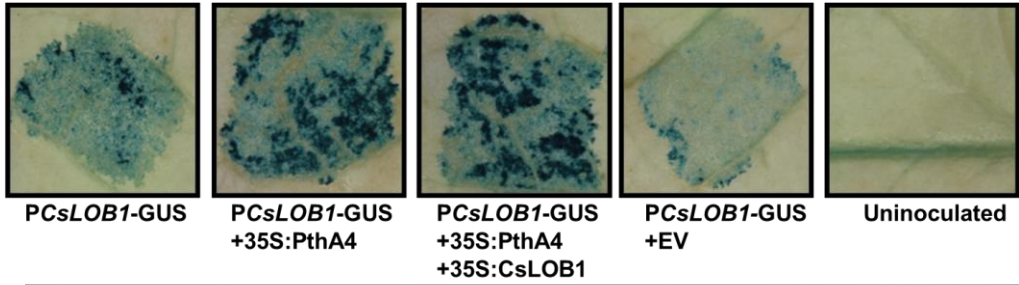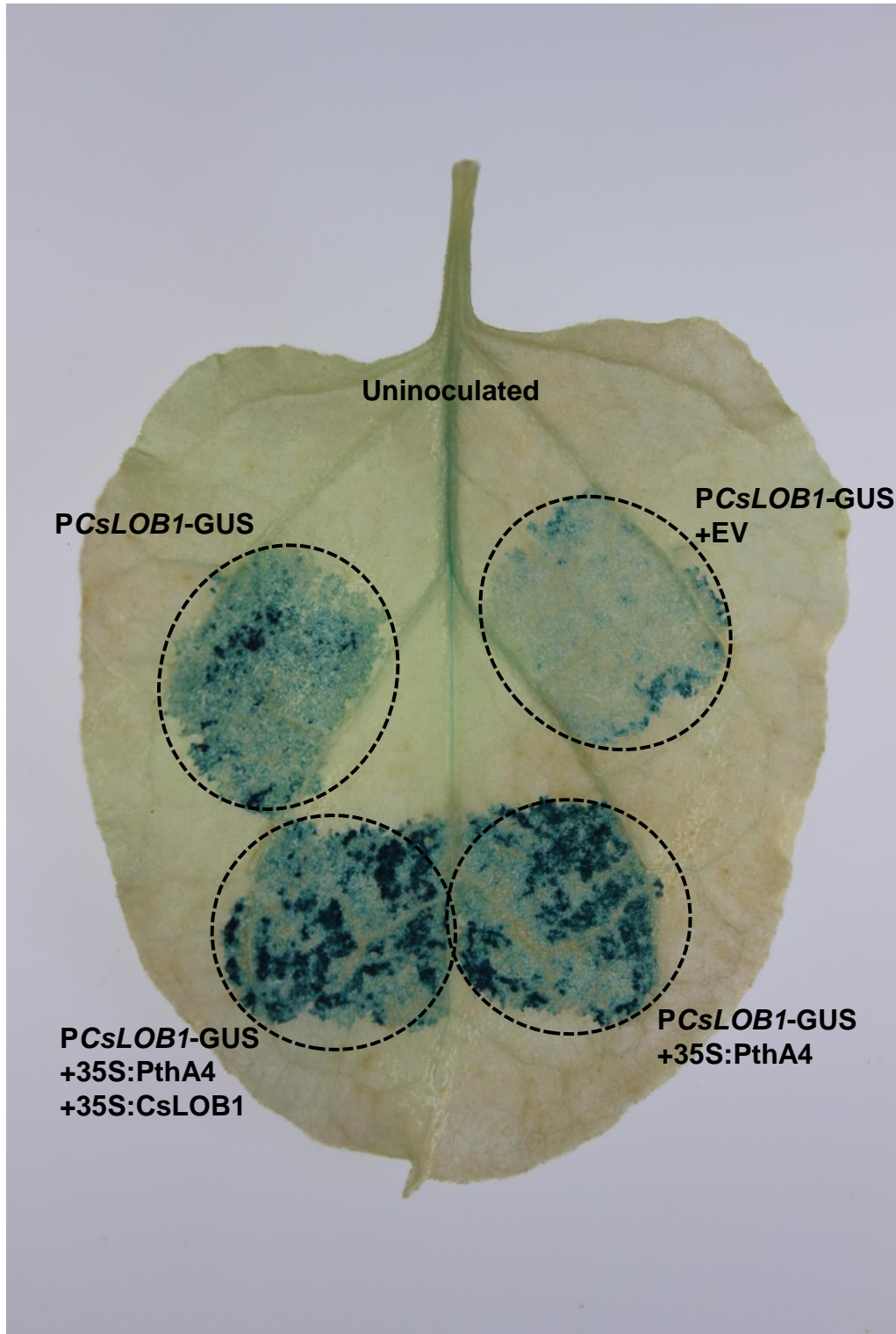**Figure 5**

Supplement: Figure 5—source data 3. [file elife-91684-fig5-data3.pdf]

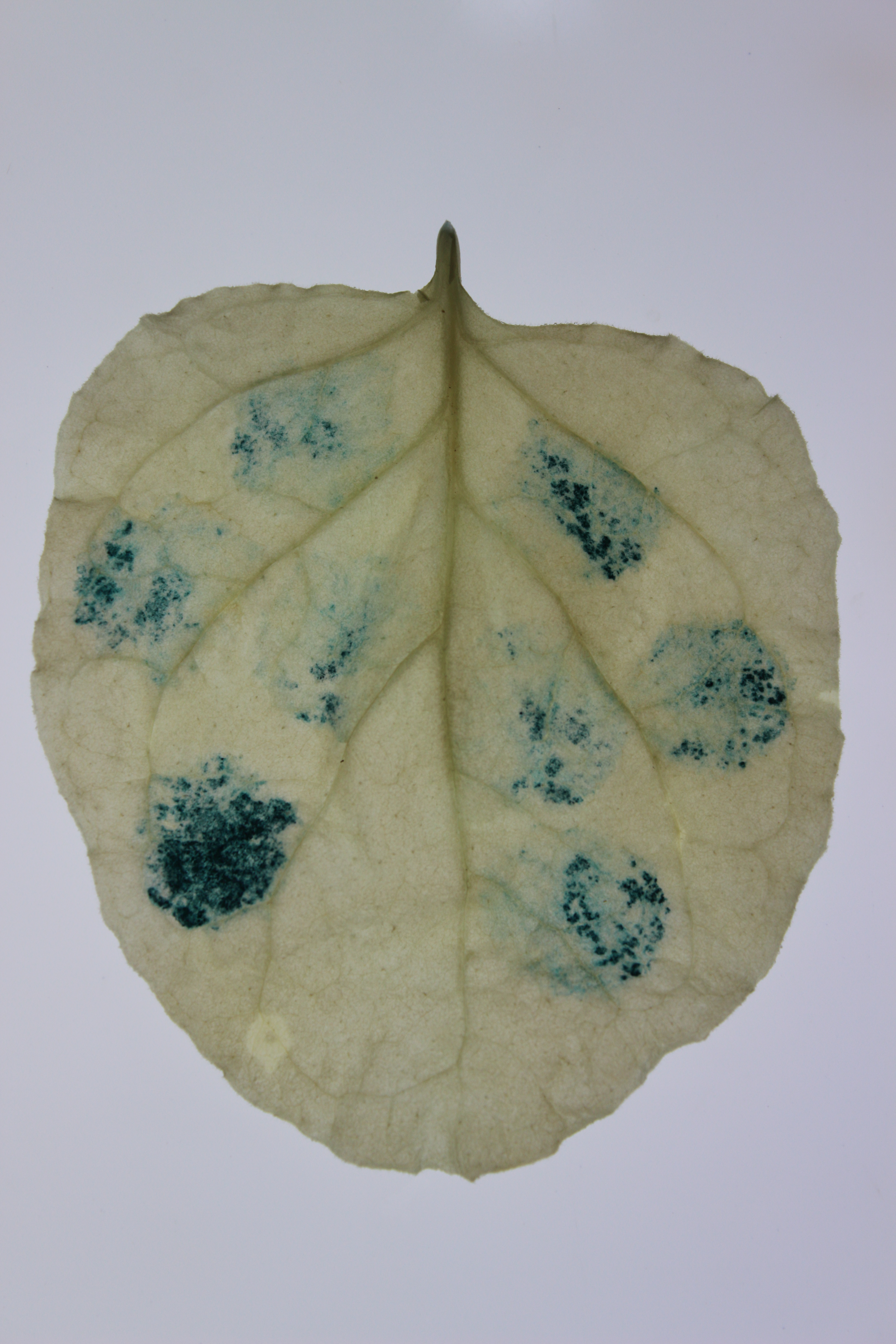

Supplement: Figure 5—source data 4. [file elife-91684-fig5-data4.zip › Figure 5- source data 4/Figure 5- source data 4.JPG]

**B****Histochemical staining**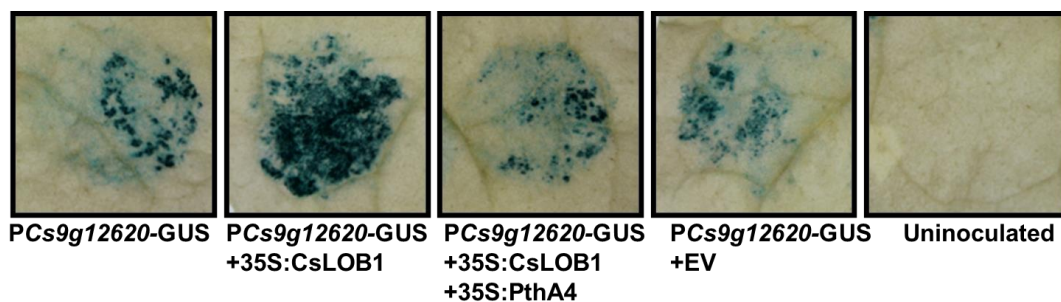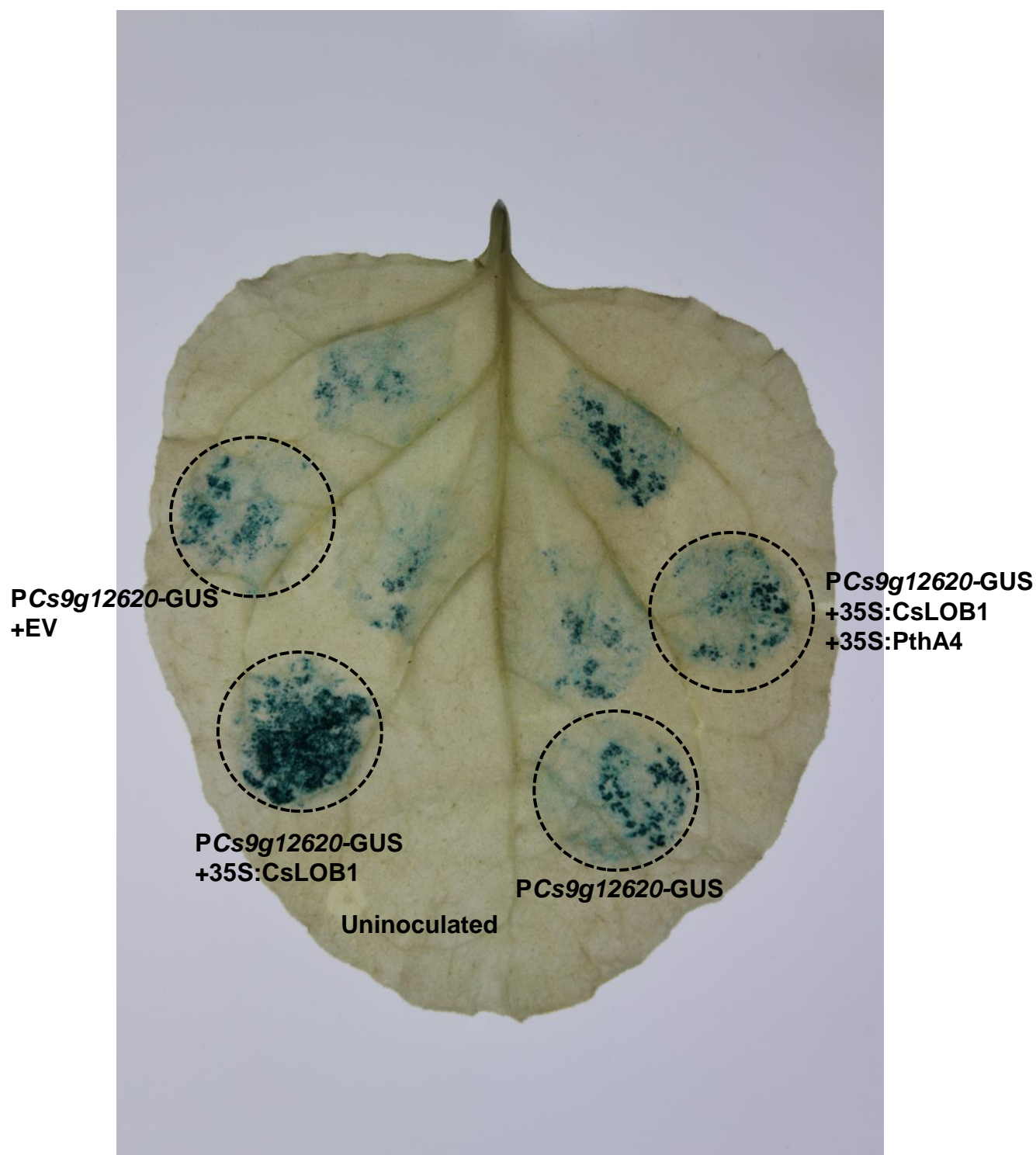**Figure 5**

Supplement: Figure 5—source data 5. [file elife-91684-fig5-data5.pdf]

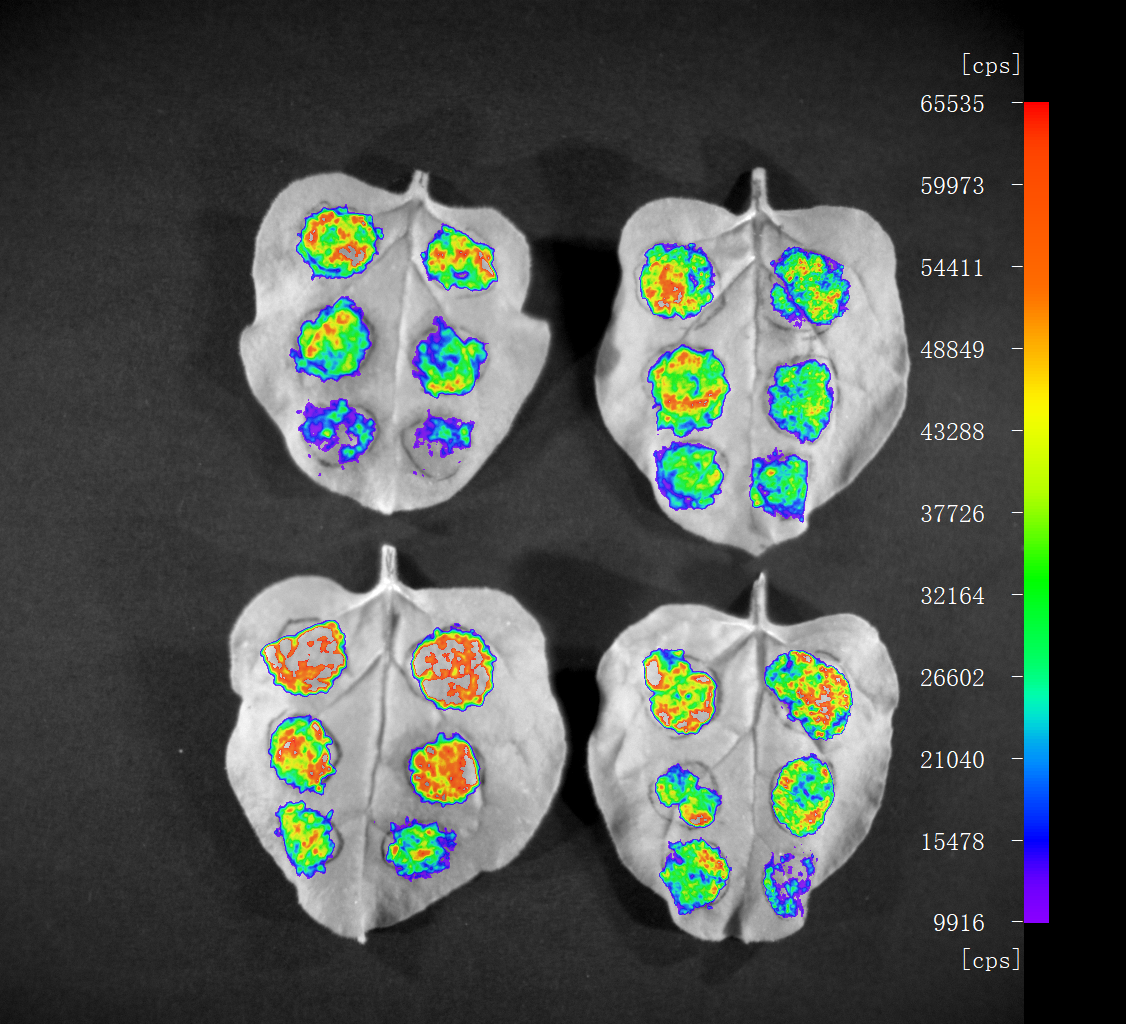

Supplement: Figure 5—source data 6. [file elife-91684-fig5-data6.zip › Figure 5- source data 6/Figure 5- source data 6.jpg]

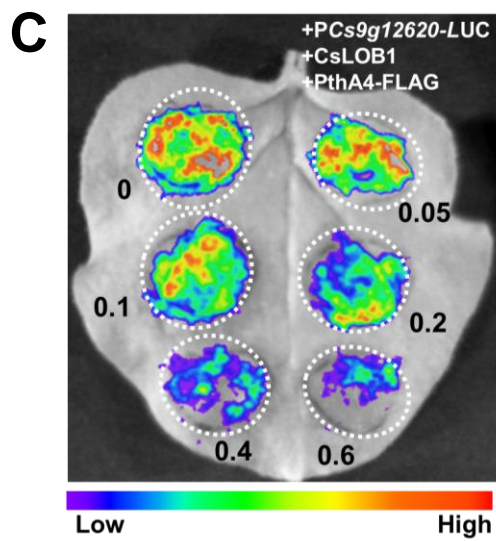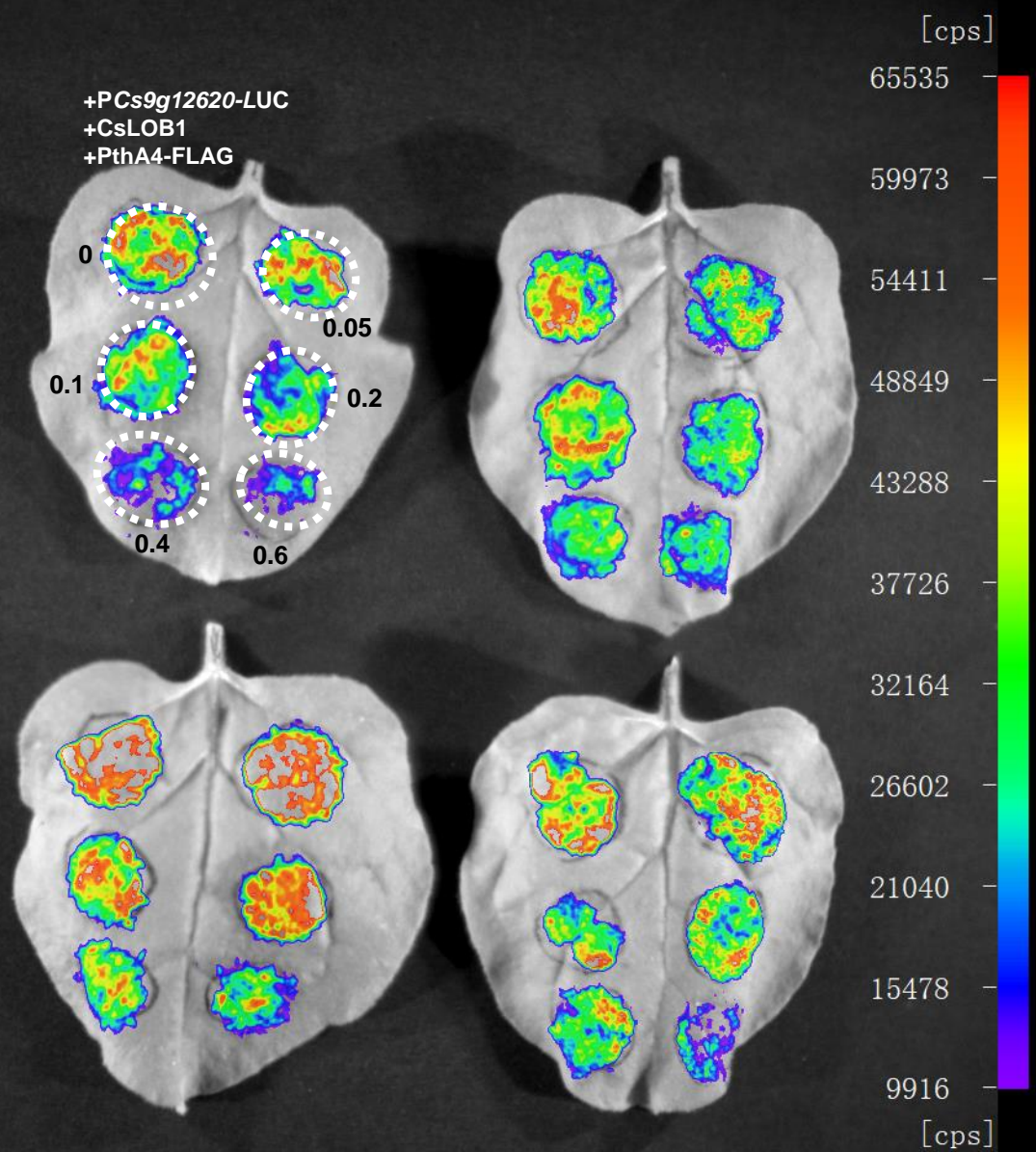

**Figure 5**

Supplement: Figure 5—source data 7. [file elife-91684-fig5-data7.pdf]

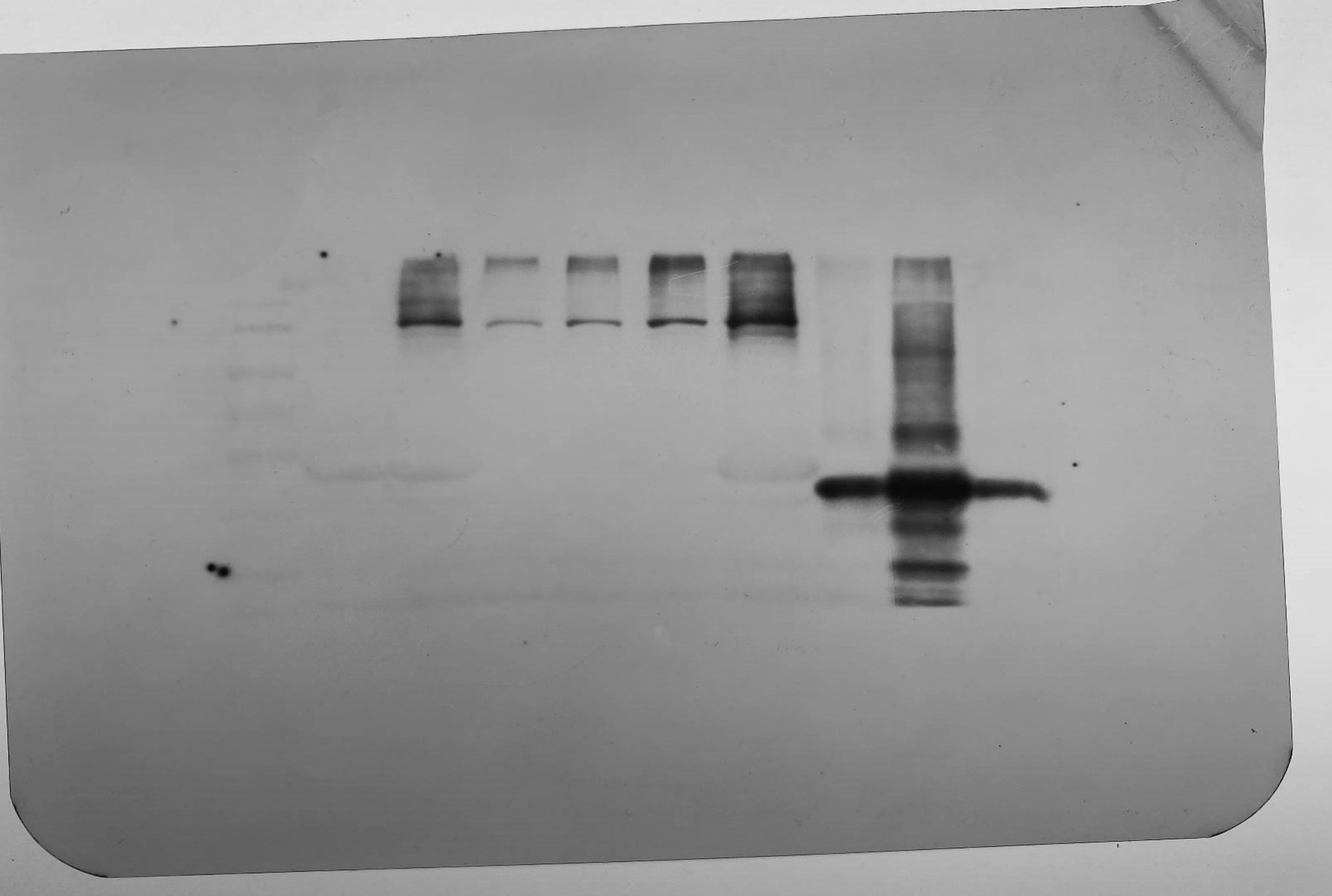

Supplement: Figure 5—source data 8. [file elife-91684-fig5-data8.zip › Figure 5- source data 8/Figure 5- source data 8.jpg]

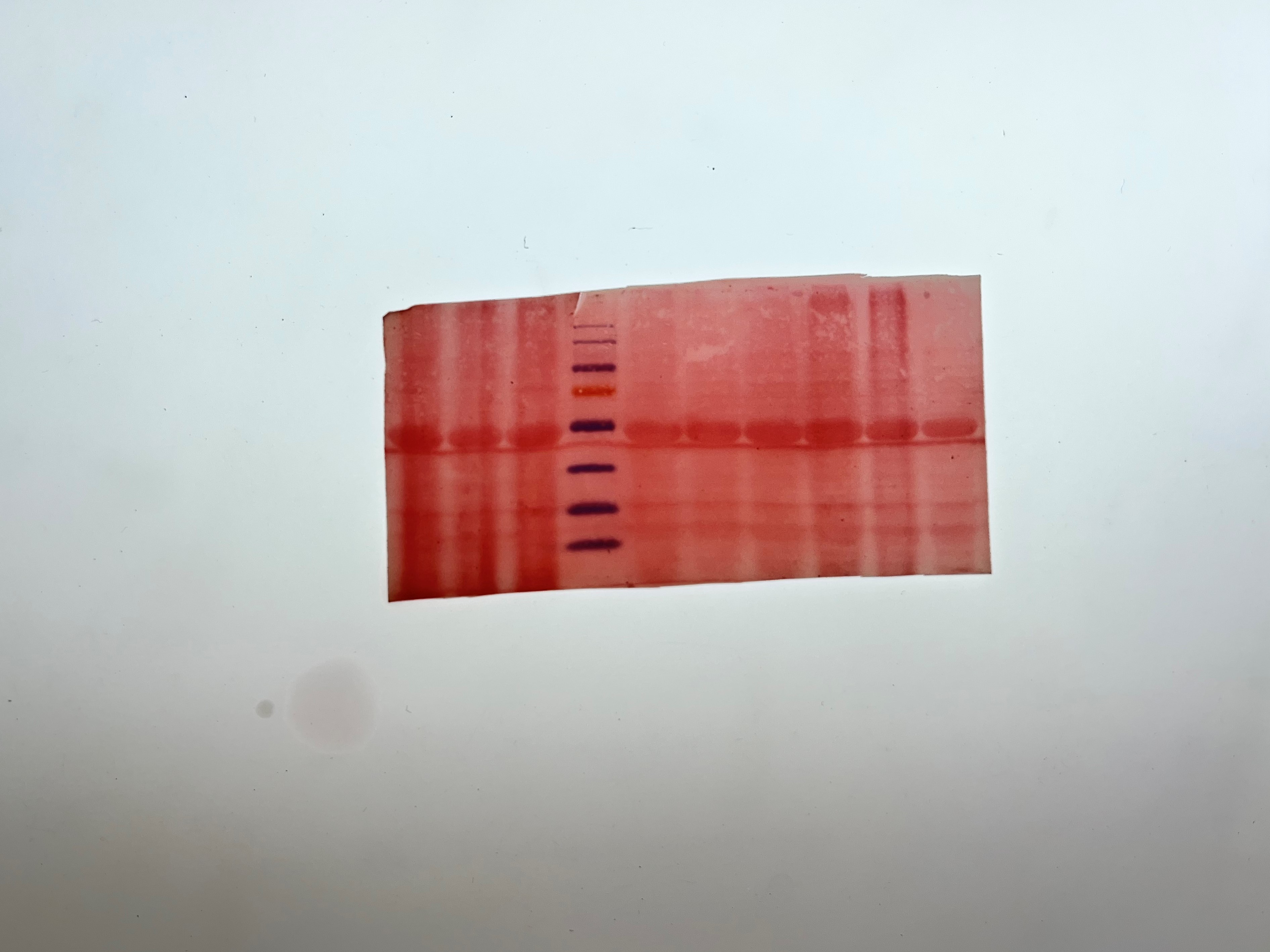

Supplement: Figure 5—source data 9. [file elife-91684-fig5-data9.zip › Figure 5- source data 9/Figure 5- source data 9.JPG]

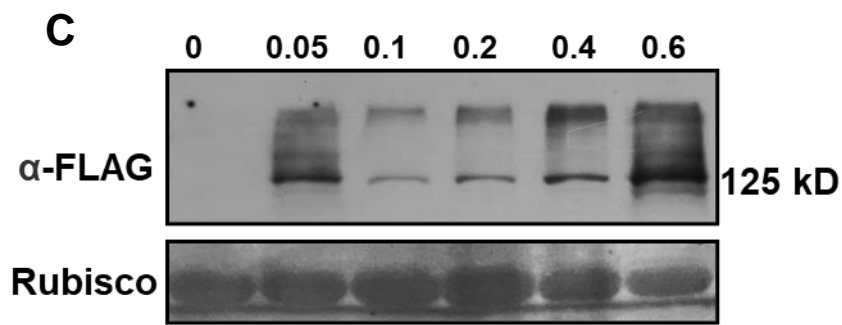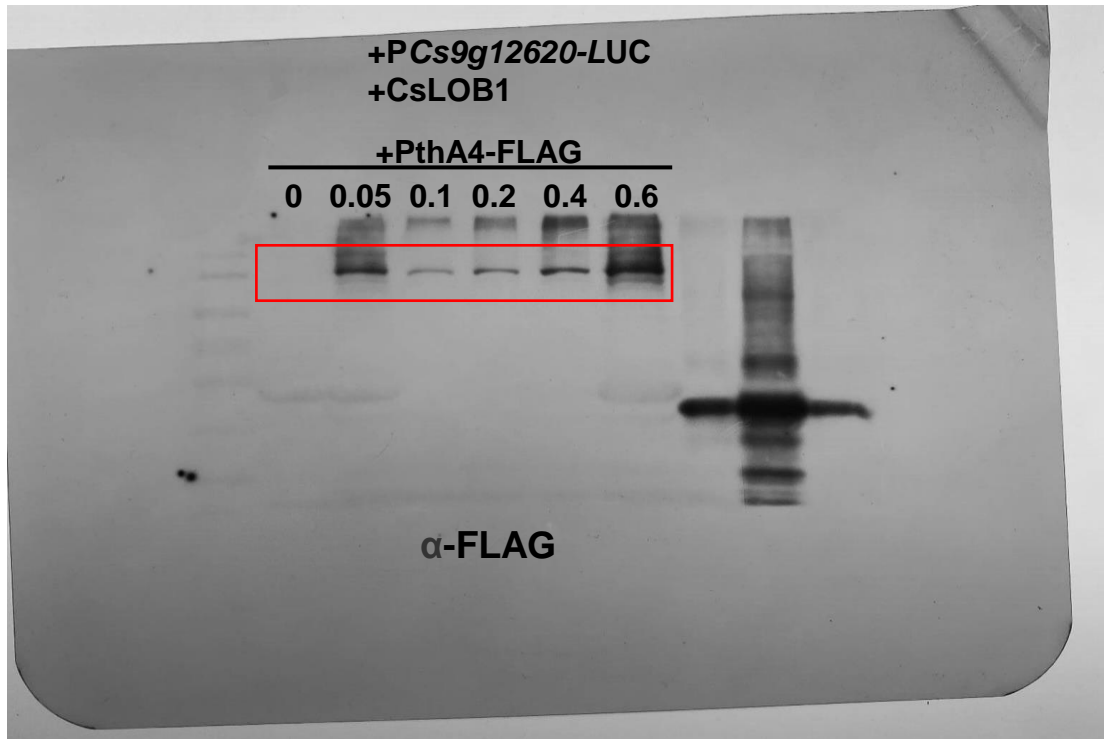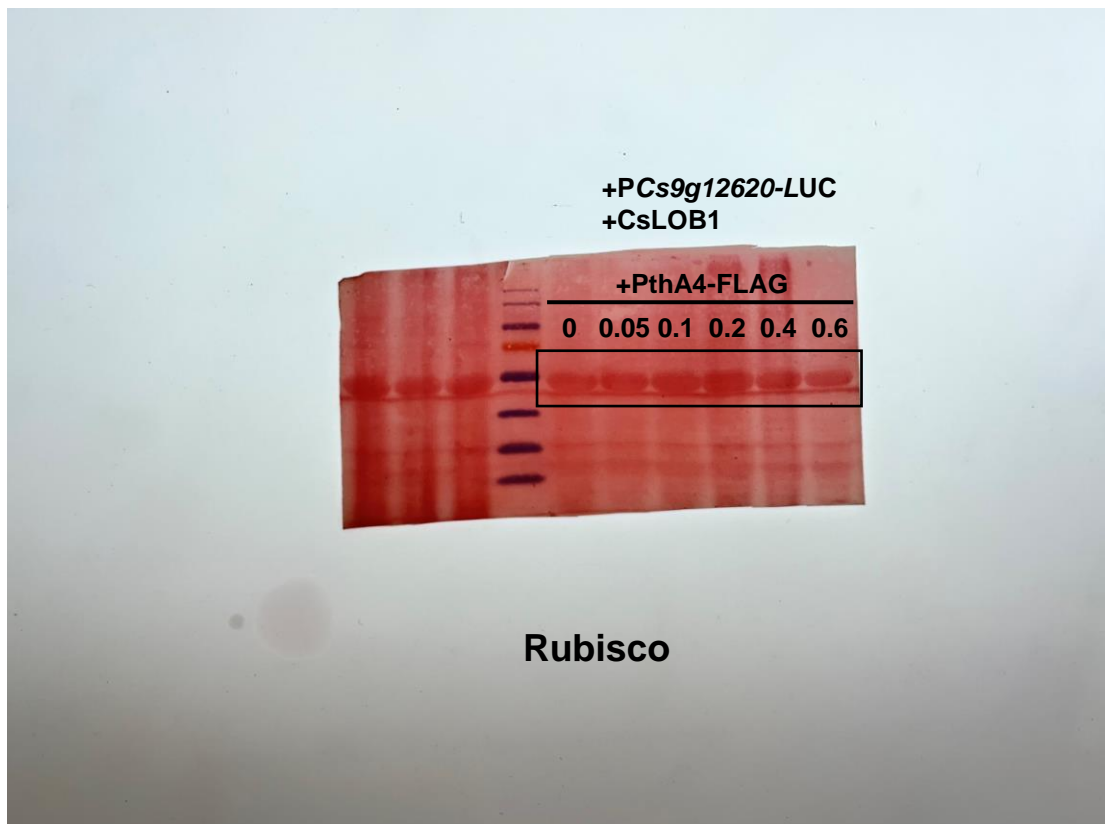

**Figure 5**

Supplement: Figure 5—source data 10. [file elife-91684-fig5-data10.pdf]

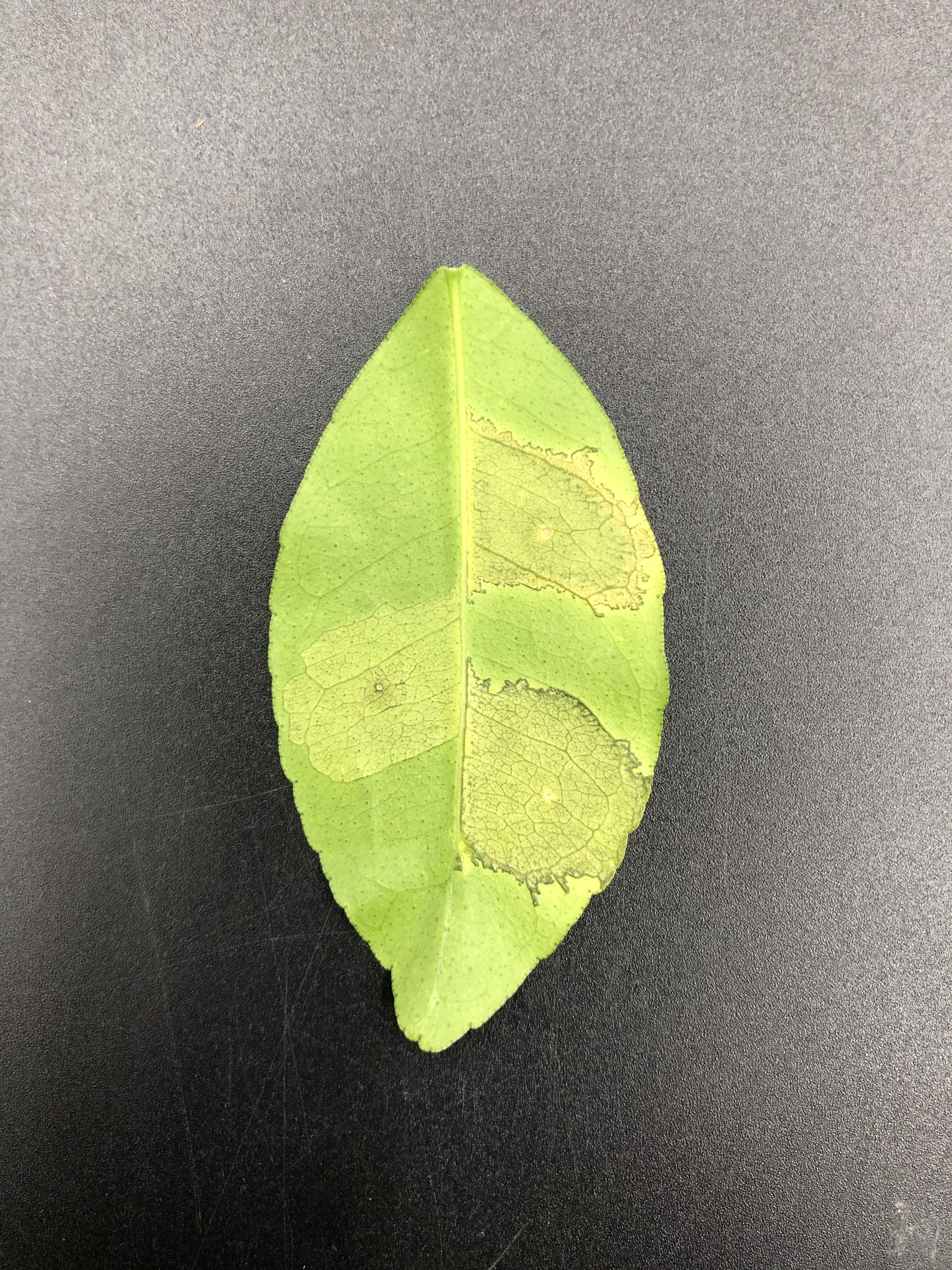

Supplement: Figure 5—source data 11. [file elife-91684-fig5-data11.zip › Figure 5- source data 11/Figure 5- source data 11.jpg]

**E**

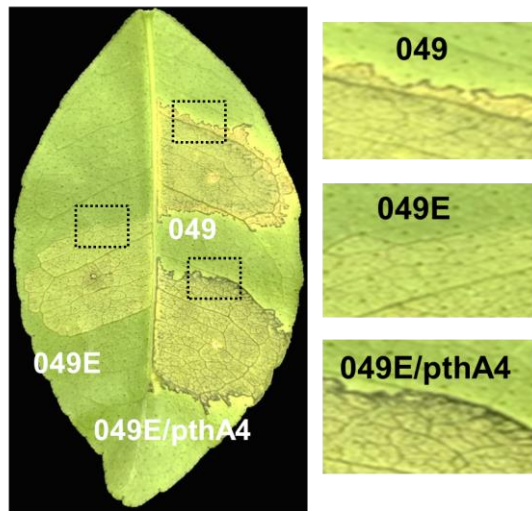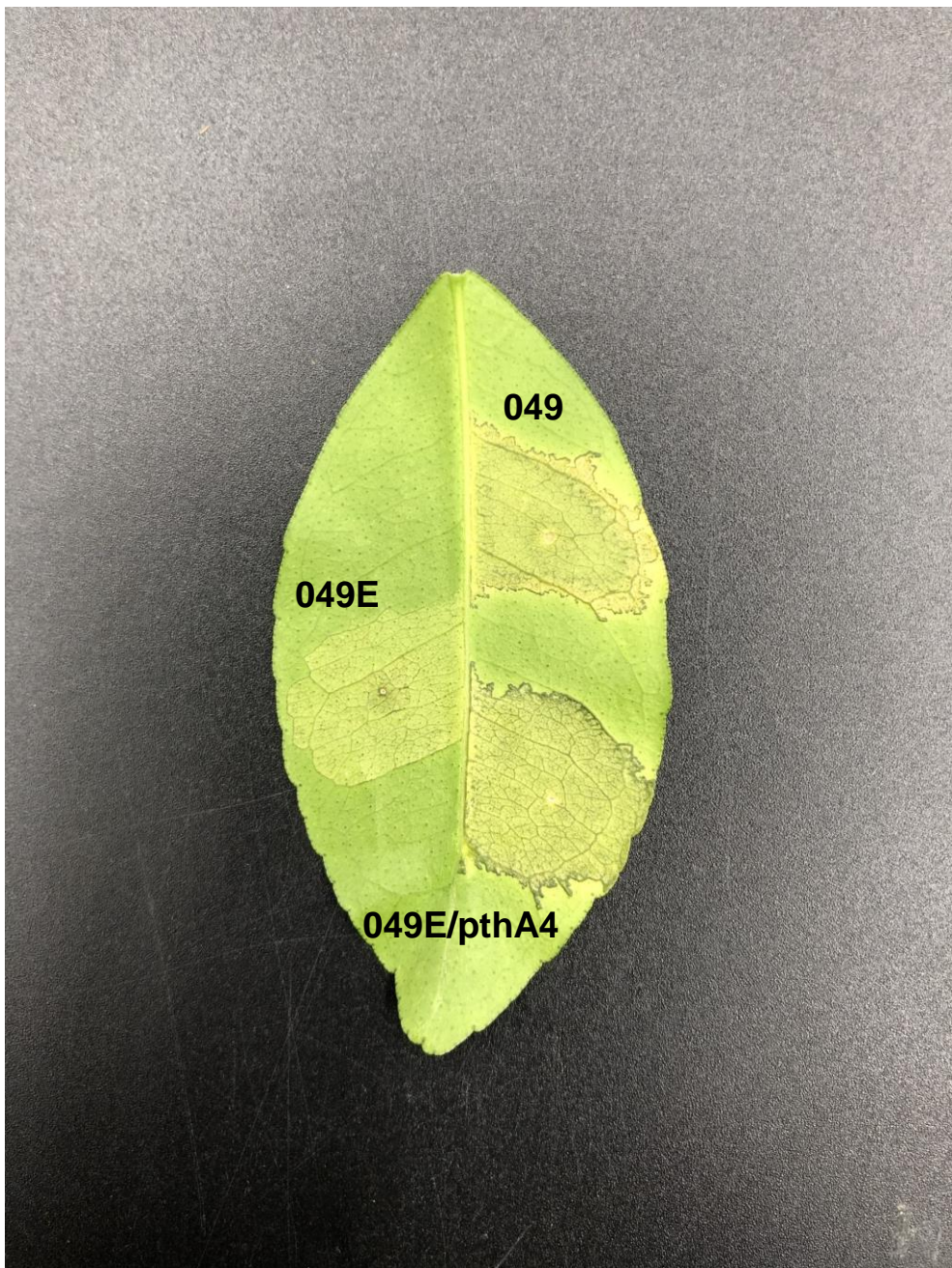

**Figure 5**

Supplement: Figure 5—source data 12. [file elife-91684-fig5-data12.pdf]

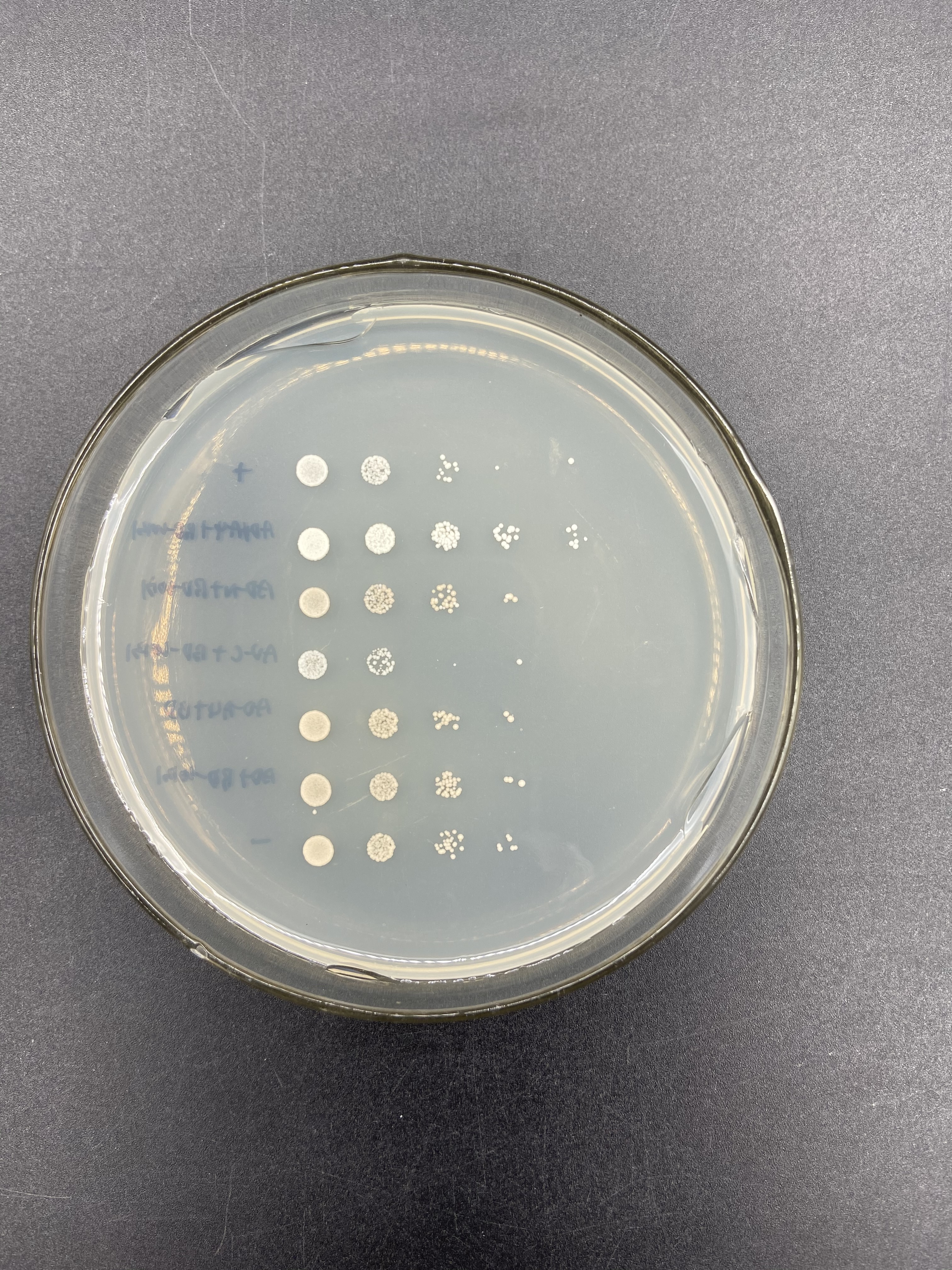

Supplement: Figure 6—source data 1. [file elife-91684-fig6-data1.zip › Figure 6- source data 1/Figure 6- source data 1.JPG]

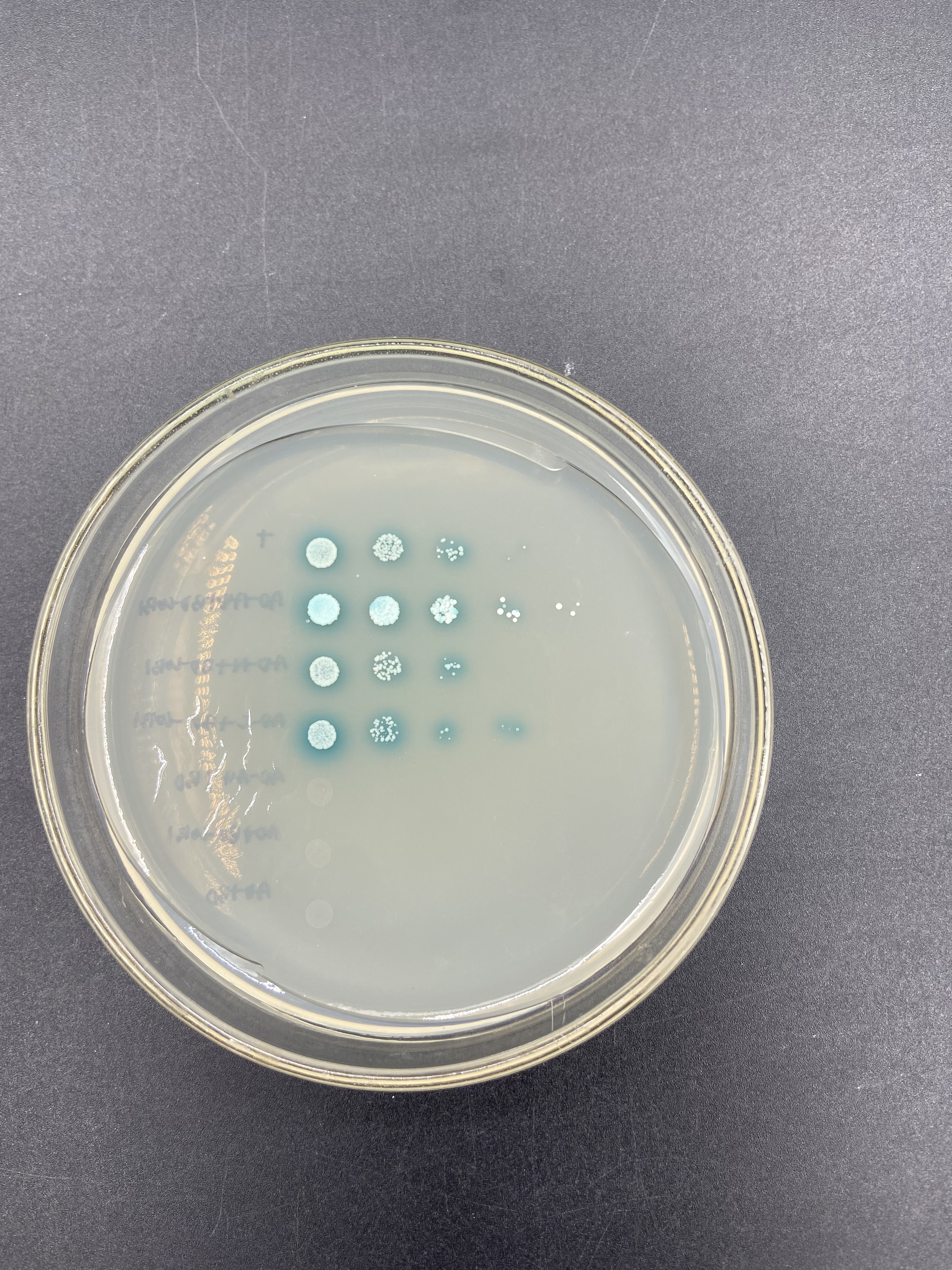

Supplement: Figure 6—source data 2. [file elife-91684-fig6-data2.zip › Figure 6- source data 2/Figure 6- source data 2.JPG]

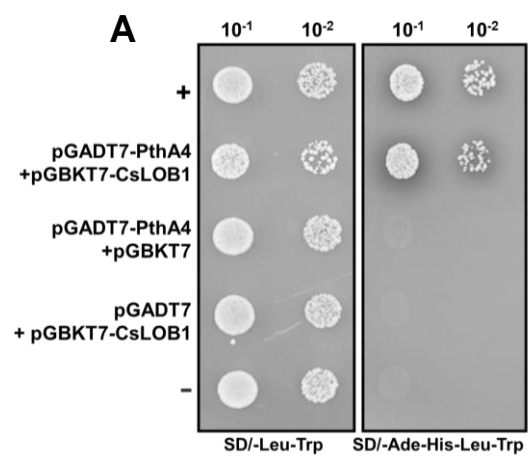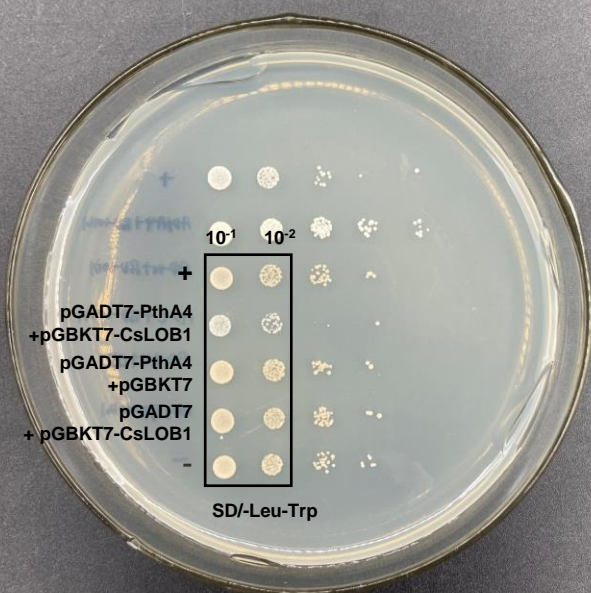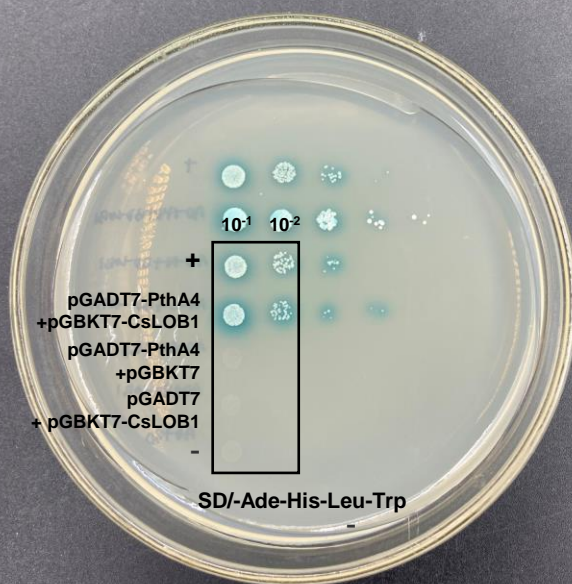

**Figure 6**

Supplement: Figure 6—source data 3. [file elife-91684-fig6-data3.pdf]

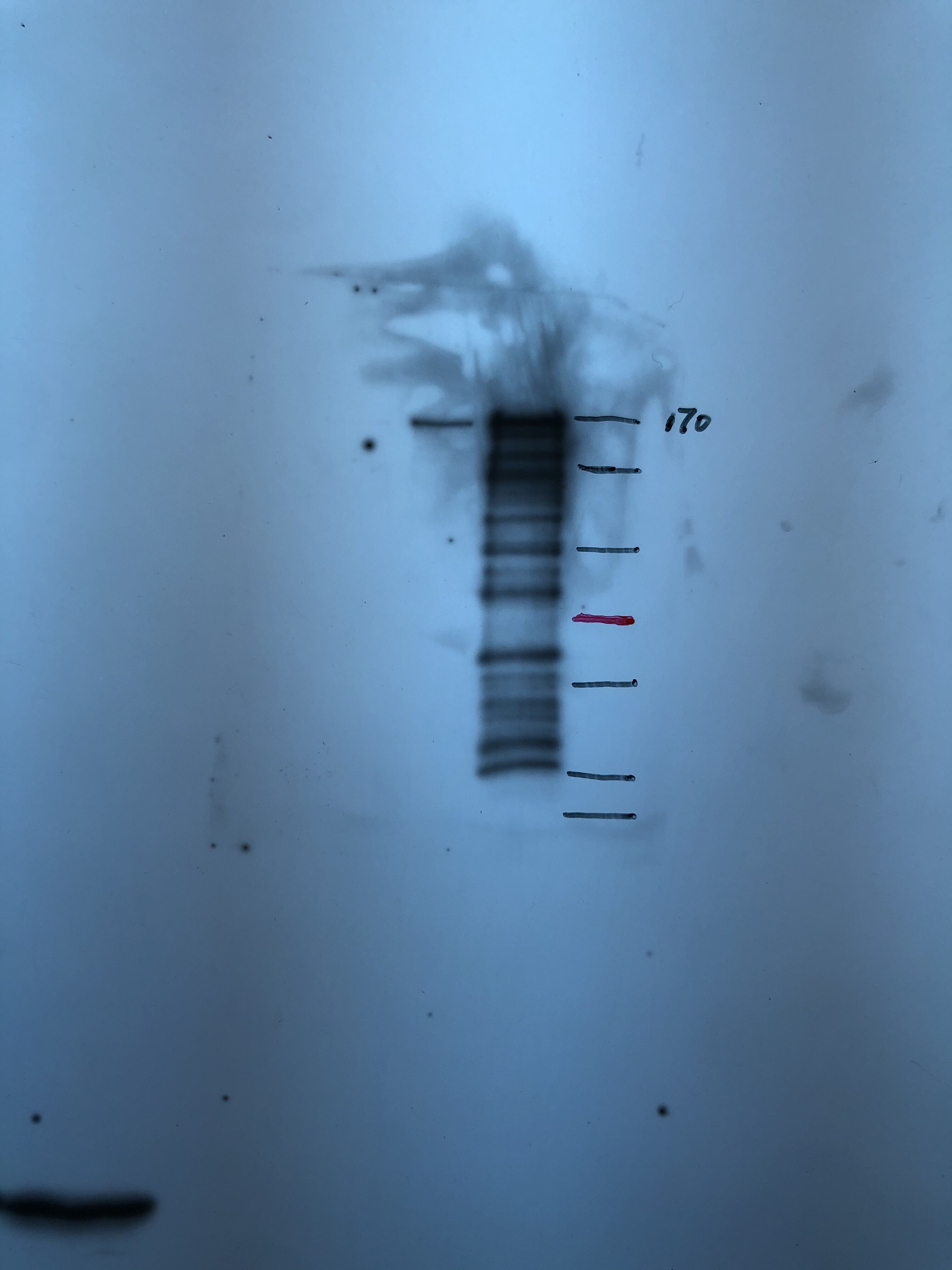

Supplement: Figure 6—source data 4. [file elife-91684-fig6-data4.zip › Figure 6- source data 4/Figure 6- source data 4.jpg]

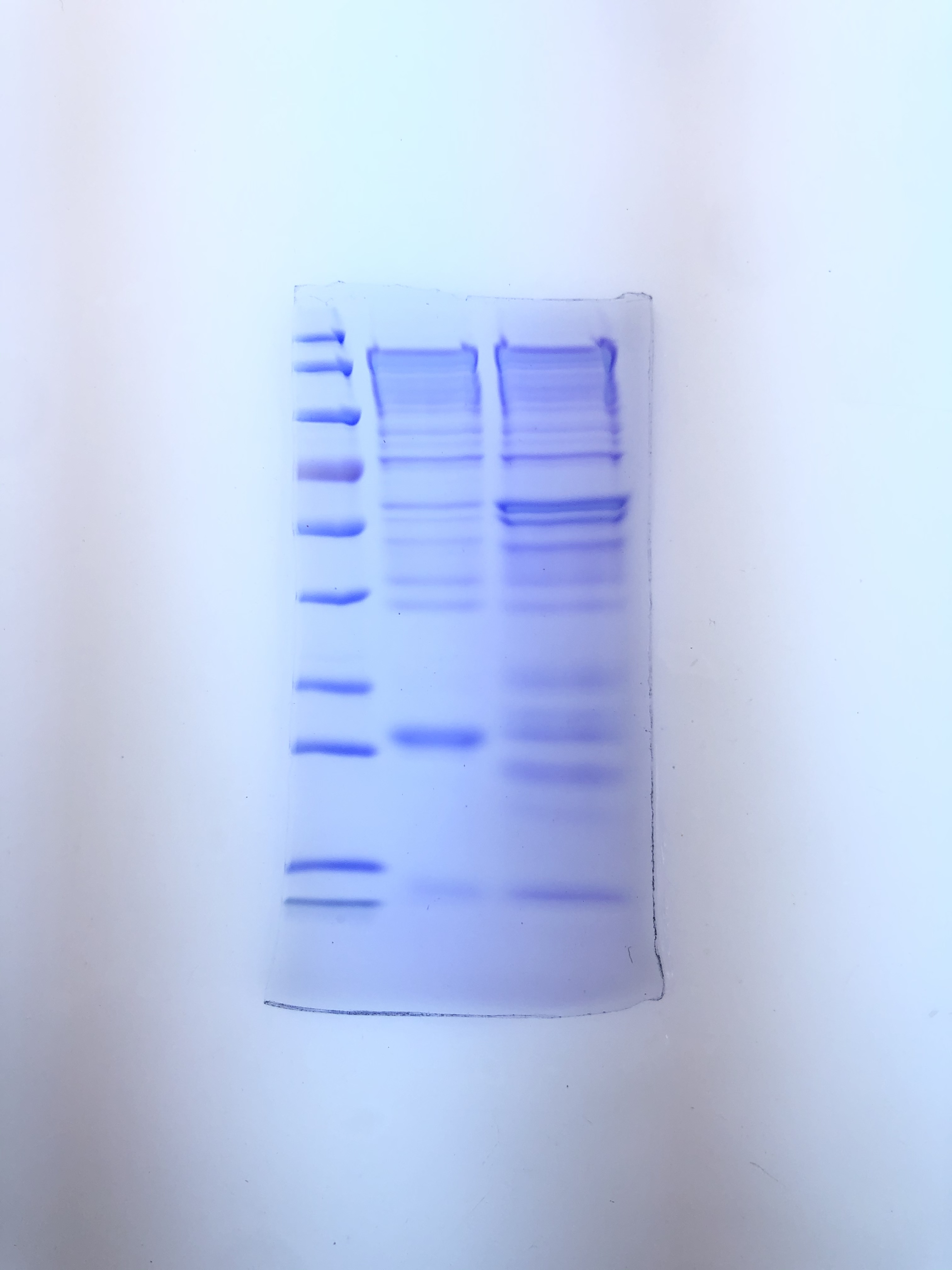

Supplement: Figure 6—source data 5. [file elife-91684-fig6-data5.zip › Figure 6- source data 5/Figure 6- source data 5.JPG]

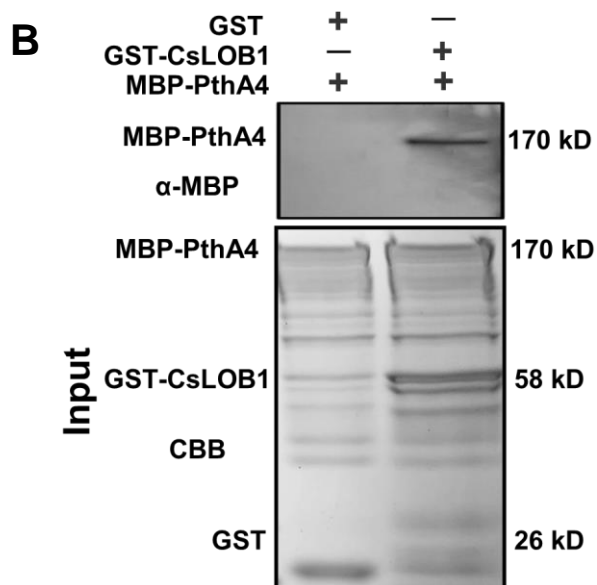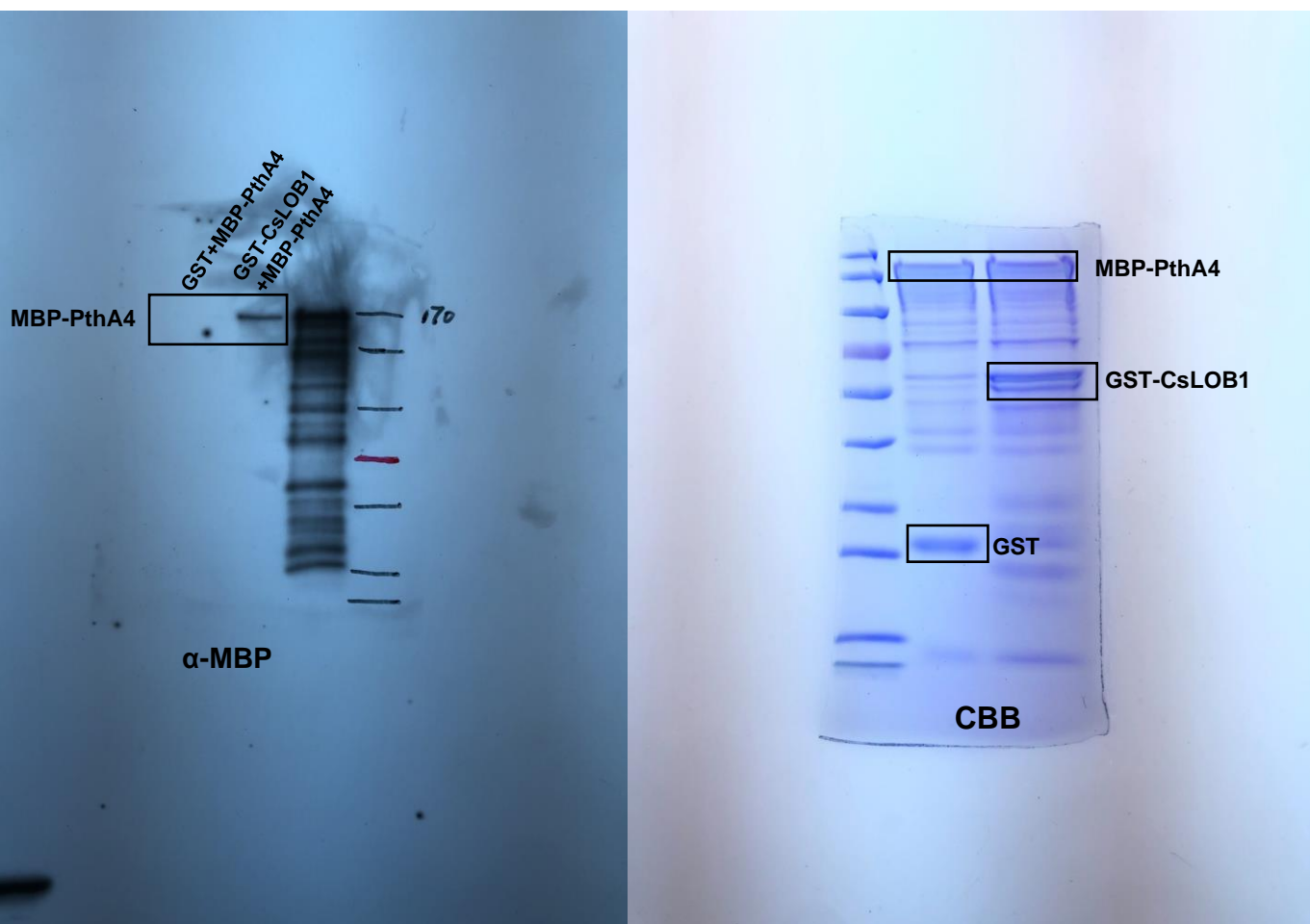

**Figure 6**

Supplement: Figure 6—source data 6. [file elife-91684-fig6-data6.pdf]

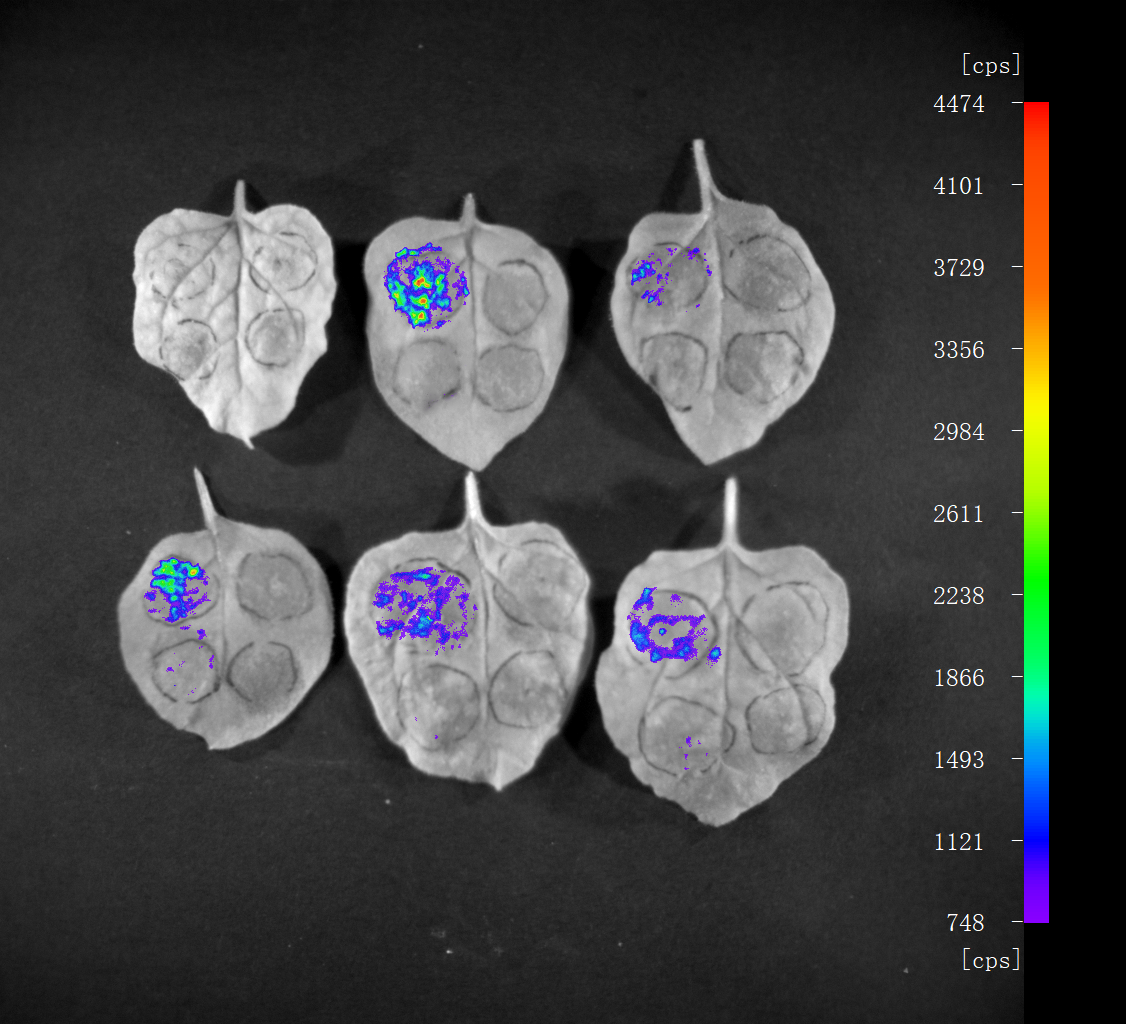

Supplement: Figure 6—source data 7. [file elife-91684-fig6-data7.zip › Figure 6- source data 7/Figure 6- source data 7.jpg]

**C**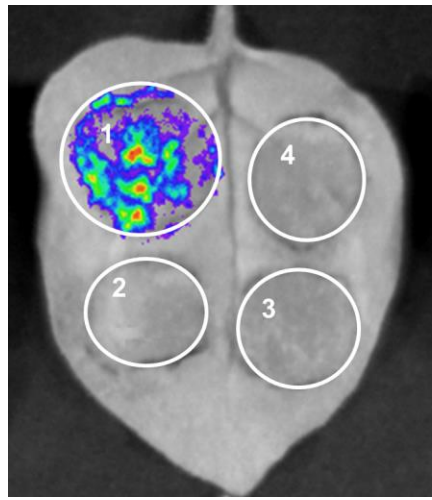

1. PthA4-nLUC+cLUC-CsLOB1
2. PthA4-nLUC+cLUC-GFP
3. GFP-nLUC+cLUC-CsLOB1
4. GFP-nLUC+cLUC-GFP

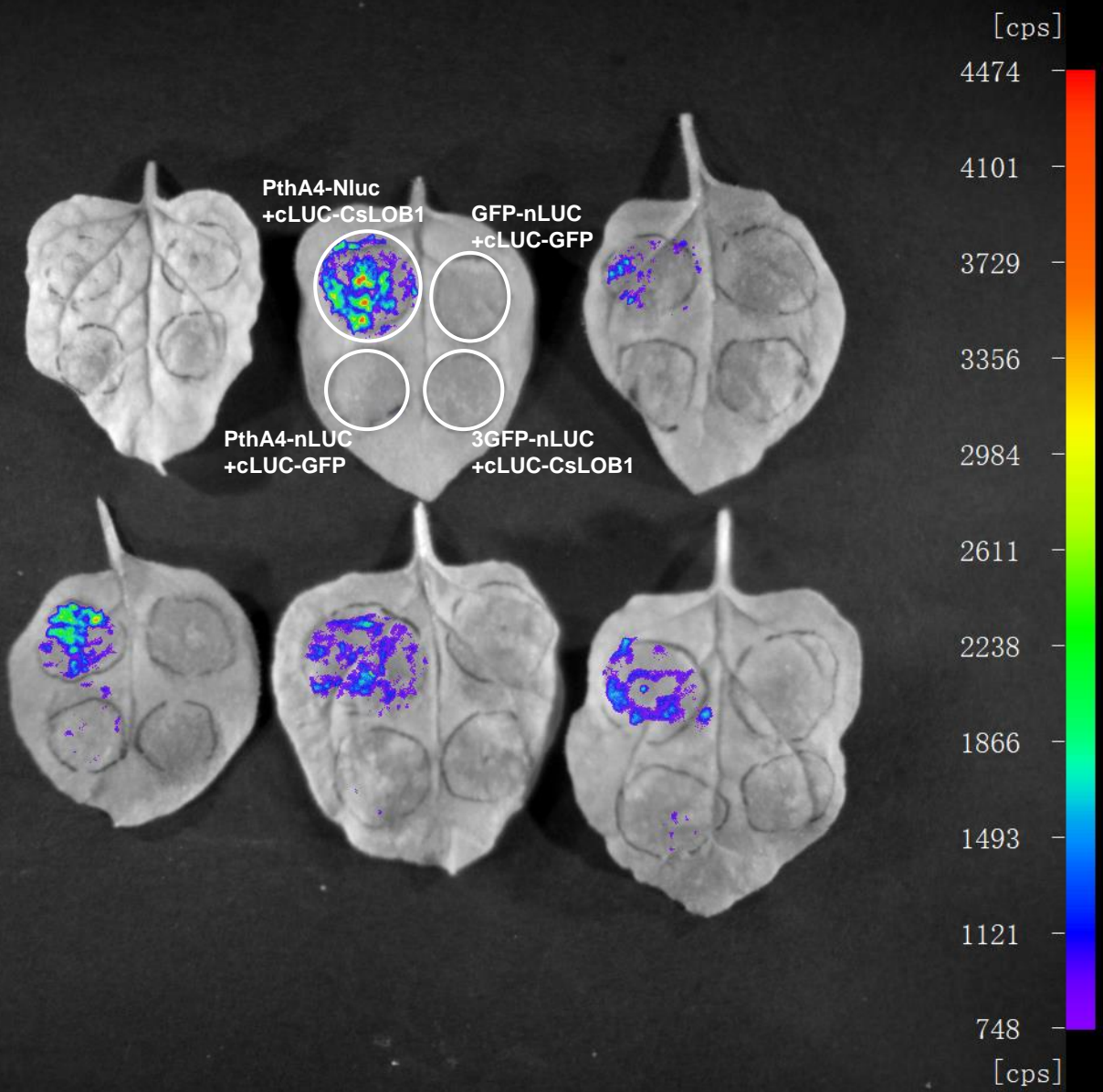**Figure 6**

Supplement: Figure 6—source data 8. [file elife-91684-fig6-data8.pdf]

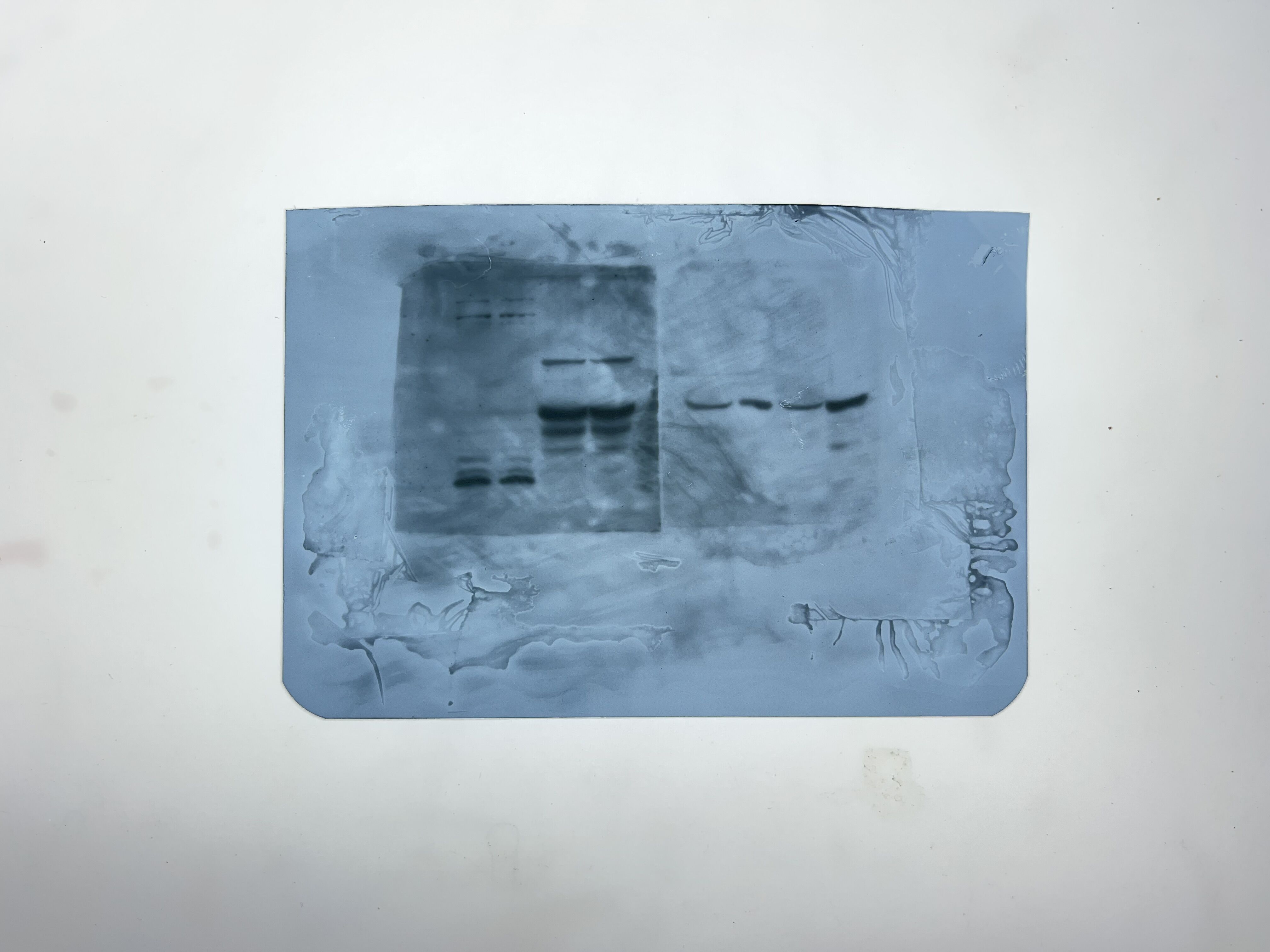

Supplement: Figure 6—source data 9. [file elife-91684-fig6-data9.zip › Figure 6- source data 9/Figure 6- source data 9.jpg]

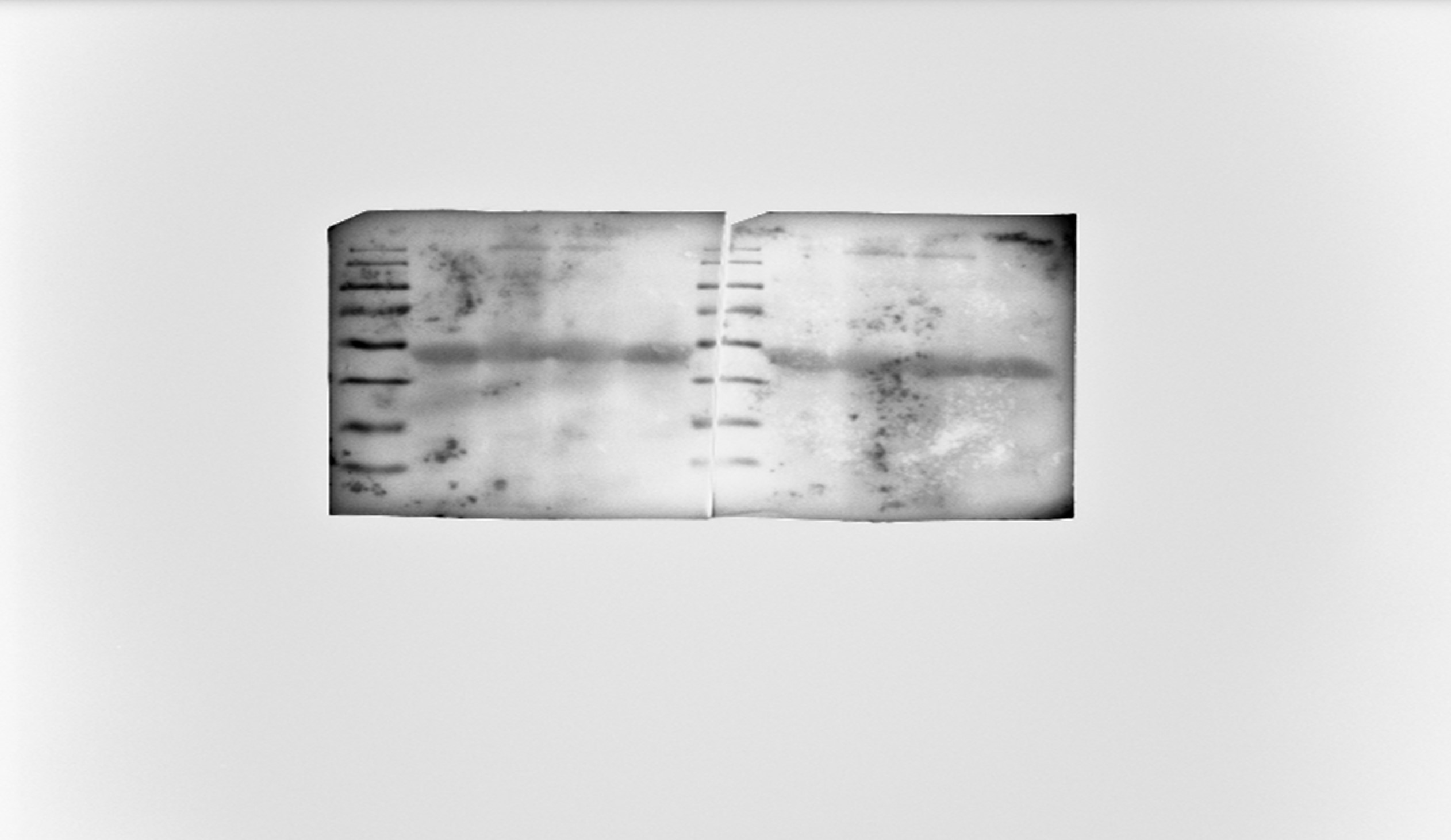

Supplement: Figure 6—source data 10. [file elife-91684-fig6-data10.zip › Figure 6- source data 10/Figure 6- source data 10.jpg]

**C**

1. PthA4-nLUC+cLUC-CsLOB1

2. PthA4-nLUC+cLUC-GFP

3. GFP-nLUC+cLUC-CsLOB1

4. GFP-nLUC+cLUC-GFP

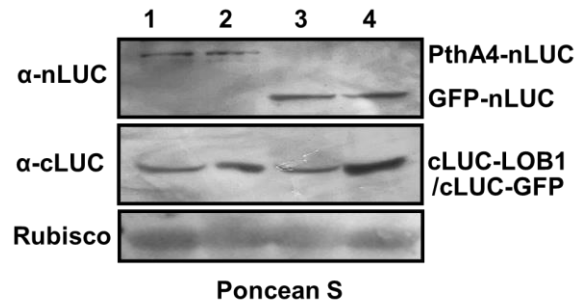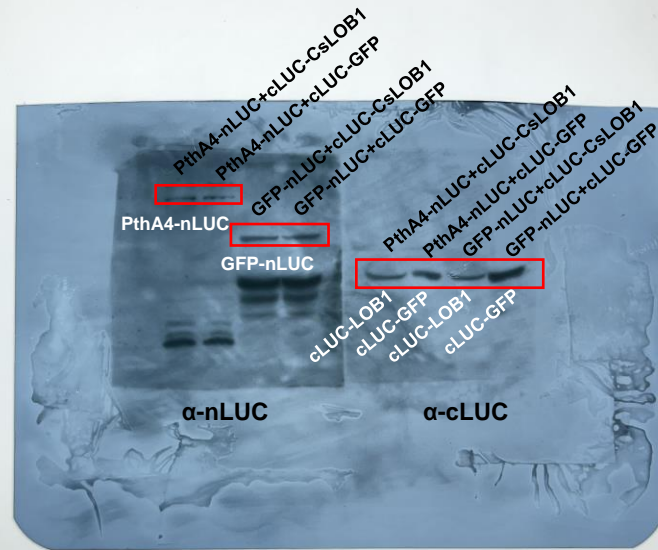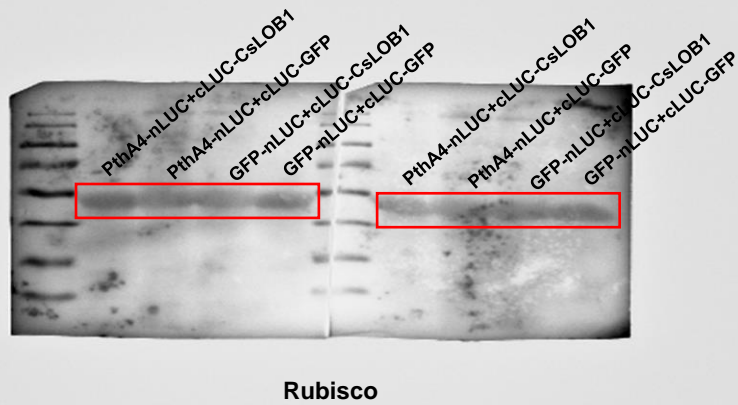

**Figure 6**

Supplement: Figure 6—source data 11. [file elife-91684-fig6-data11.pdf]

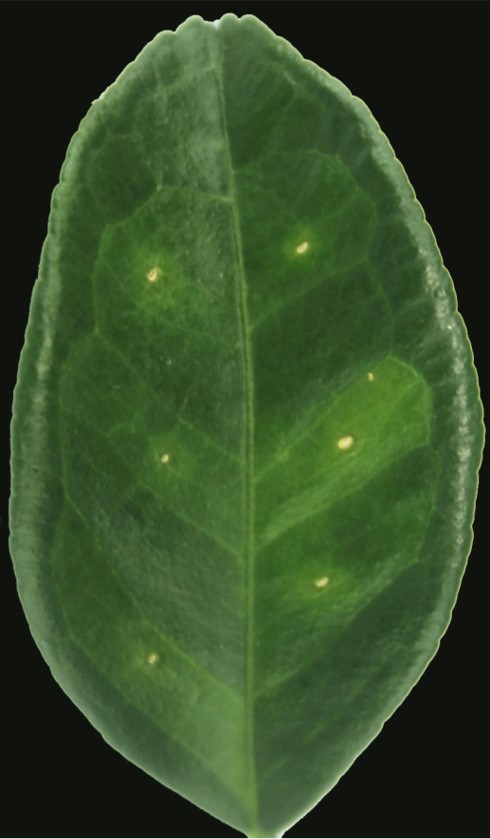

Supplement: Figure 7—source data 2. [file elife-91684-fig7-data2.zip › Figure 7- source data 2/Figure 7- source data 2.jpg]

**A**

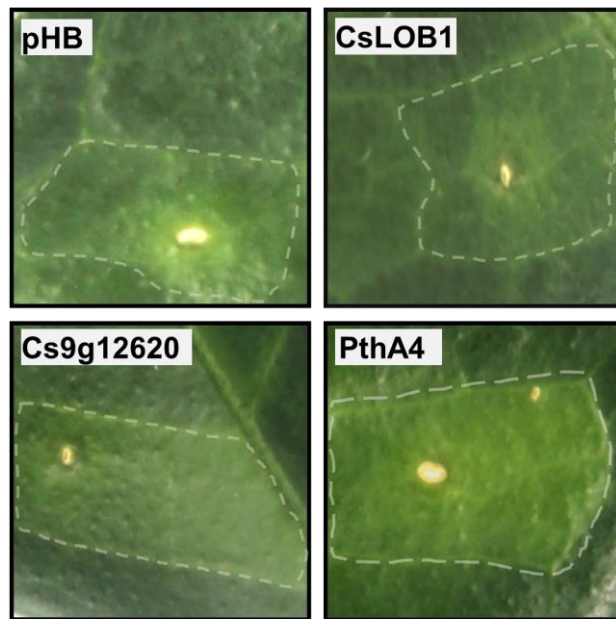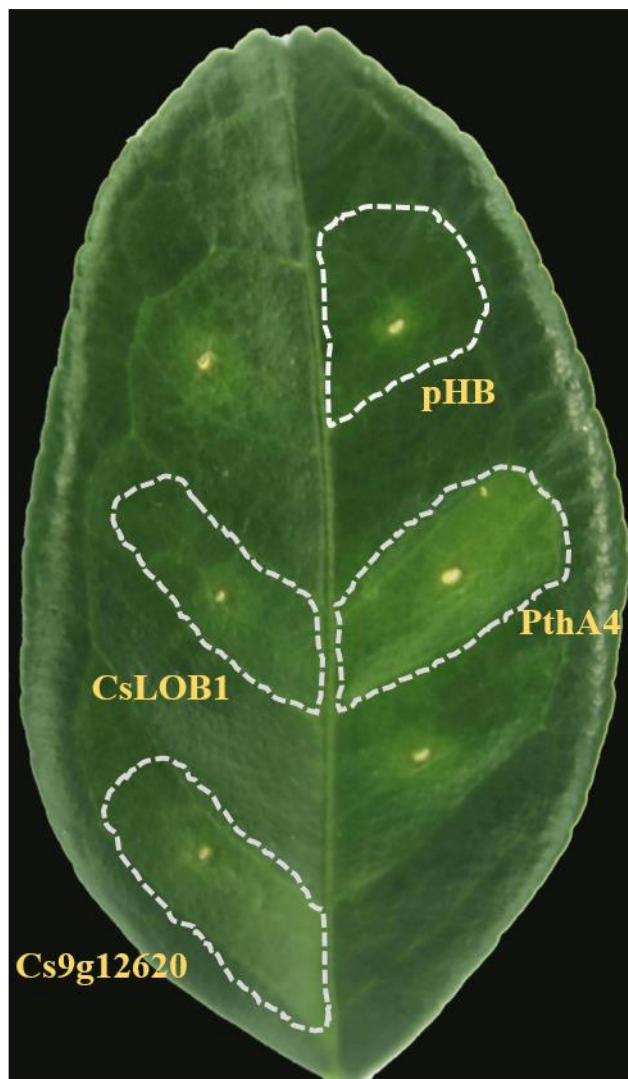

**Figure 7**

Supplement: Figure 7—source data 3. [file elife-91684-fig7-data3.pdf]

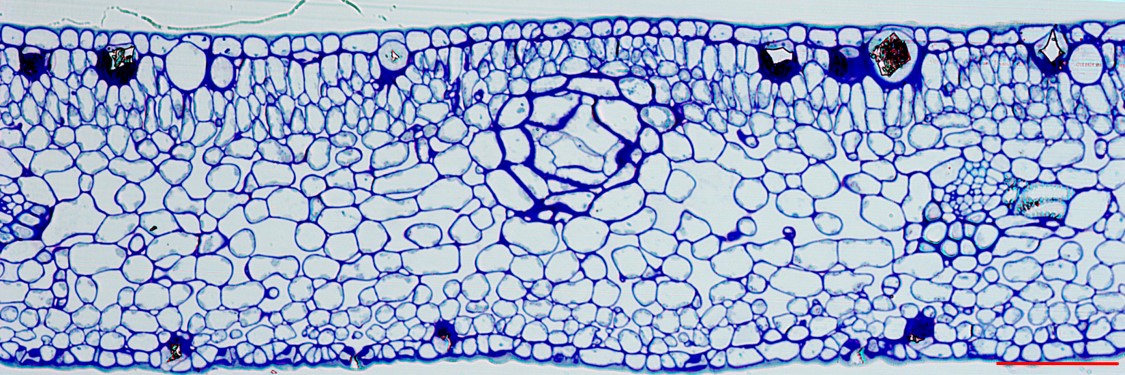

Supplement: Figure 7—source data 4. [file elife-91684-fig7-data4.zip › Figure 7- source data 4/Figure 7- source data 4.jpg]

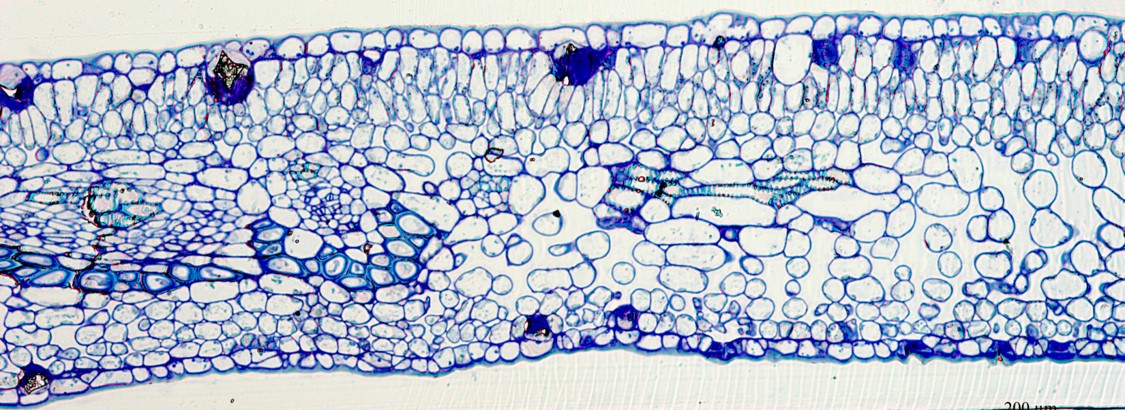

Supplement: Figure 7—source data 5. [file elife-91684-fig7-data5.zip › Figure 7- source data 5/Figure 7- source data 5.jpg]

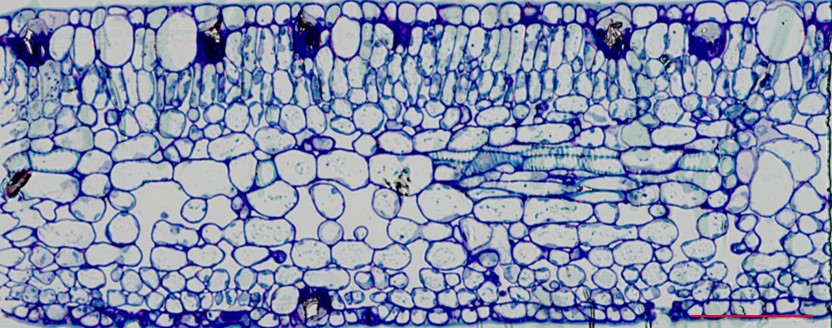

Supplement: Figure 7—source data 6. [file elife-91684-fig7-data6.zip › Figure 7- source data 6/Figure 7- source data 6.jpg]

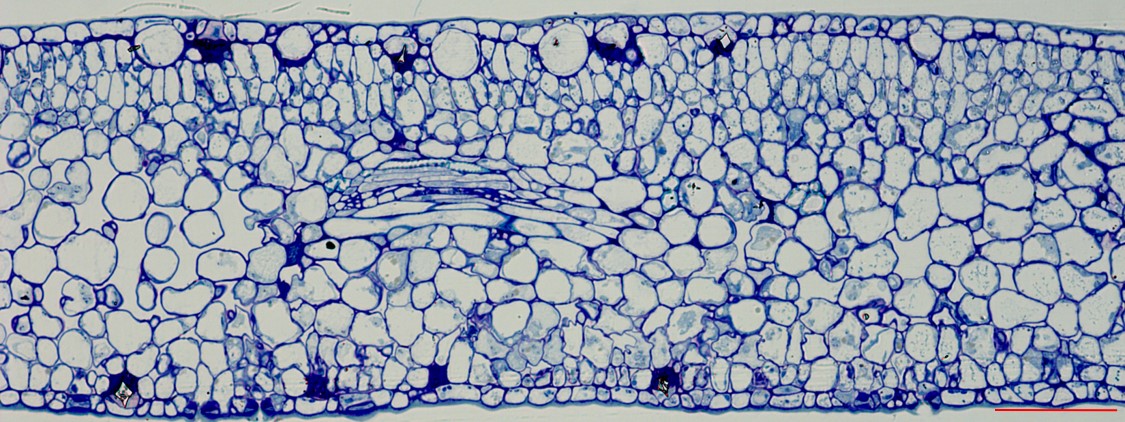

Supplement: Figure 7—source data 7. [file elife-91684-fig7-data7.zip › Figure 7- source data 7/Figure 7- source data 7.jpg]

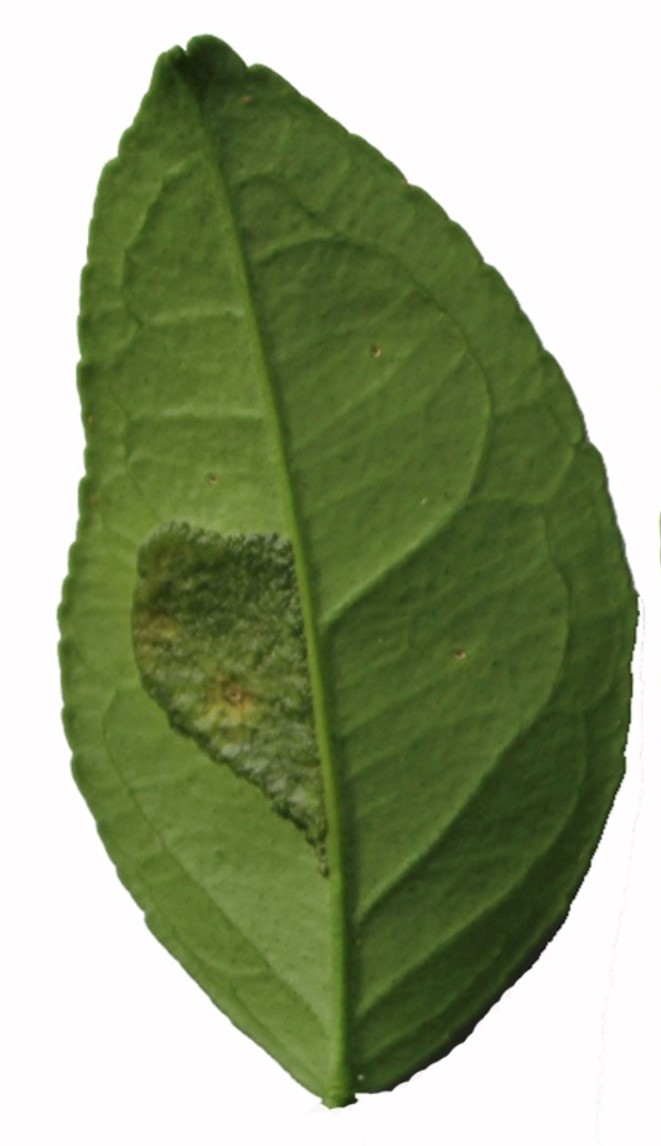

Supplement: Figure 7—source data 8. [file elife-91684-fig7-data8.zip › Figure 7- source data 8/Figure 7- source data 8.jpg]

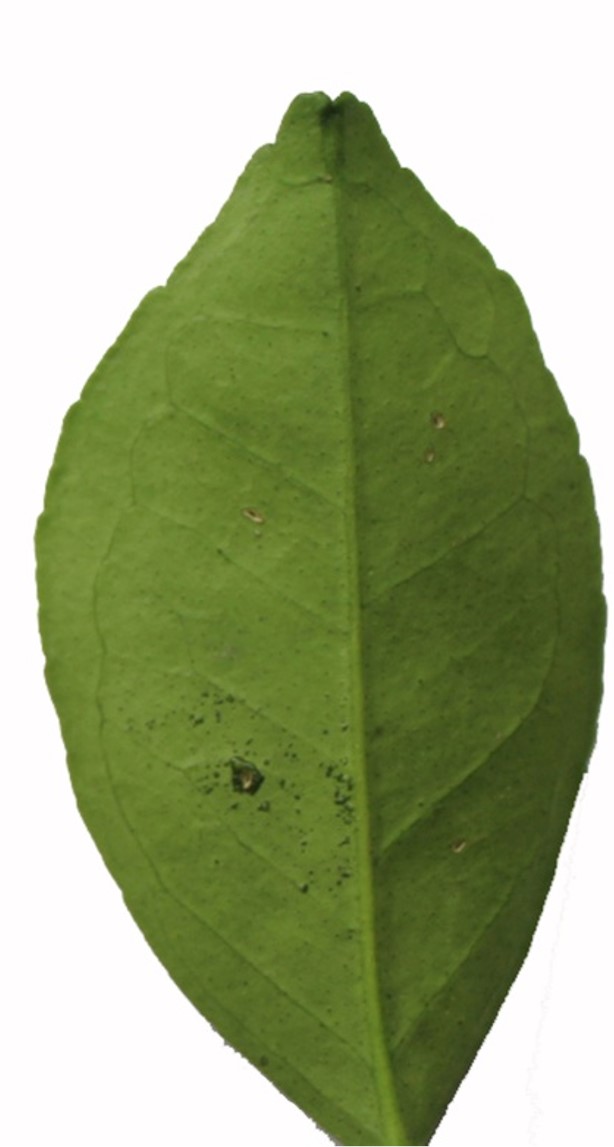

Supplement: Figure 7—source data 9. [file elife-91684-fig7-data9.zip › Figure 7- source data 9/Figure 7- source data 9.jpg]

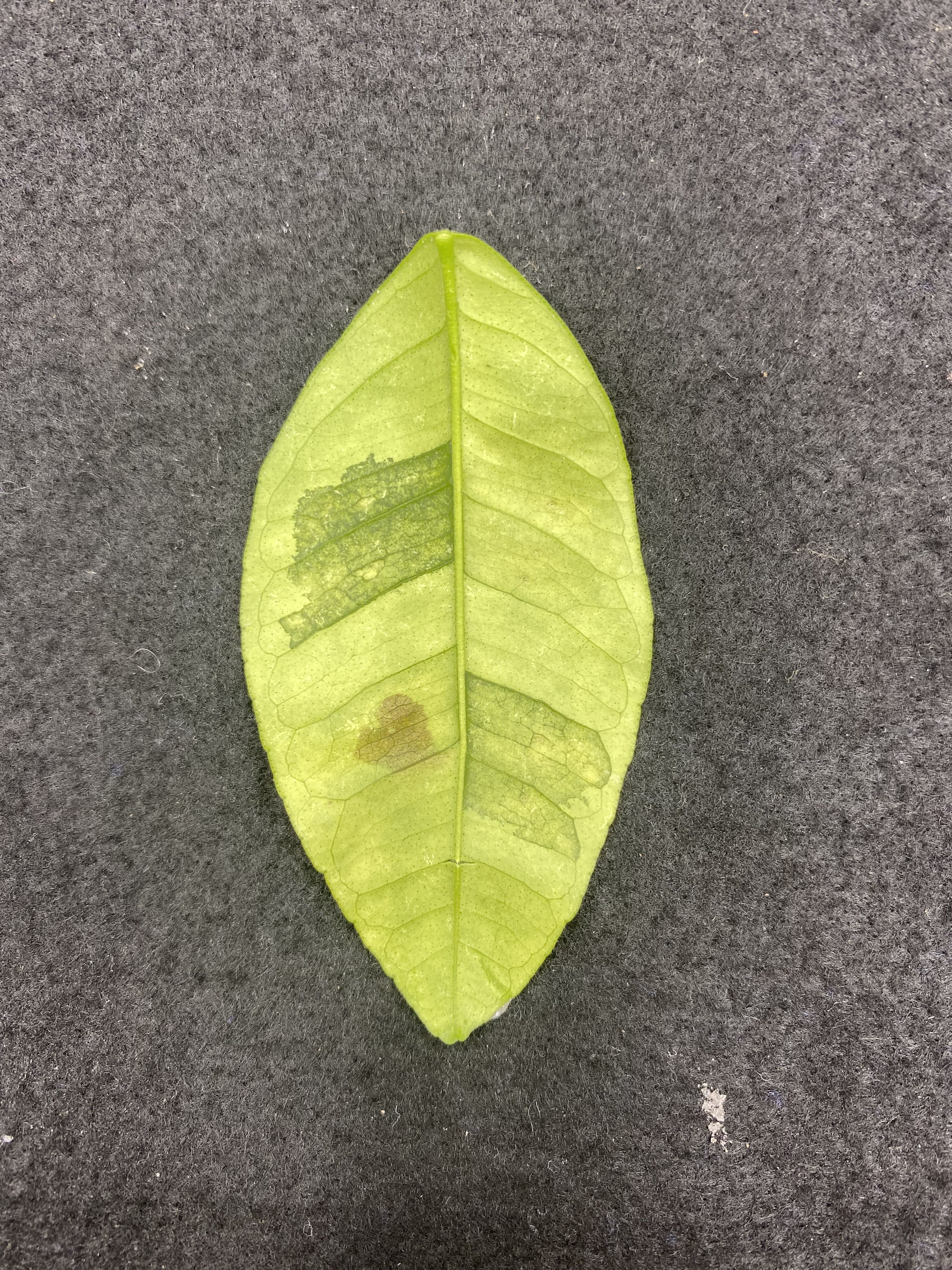

Supplement: Figure 7—source data 10. [file elife-91684-fig7-data10.zip › Figure 7- source data 10/Figure 7- source data 10.jpg]

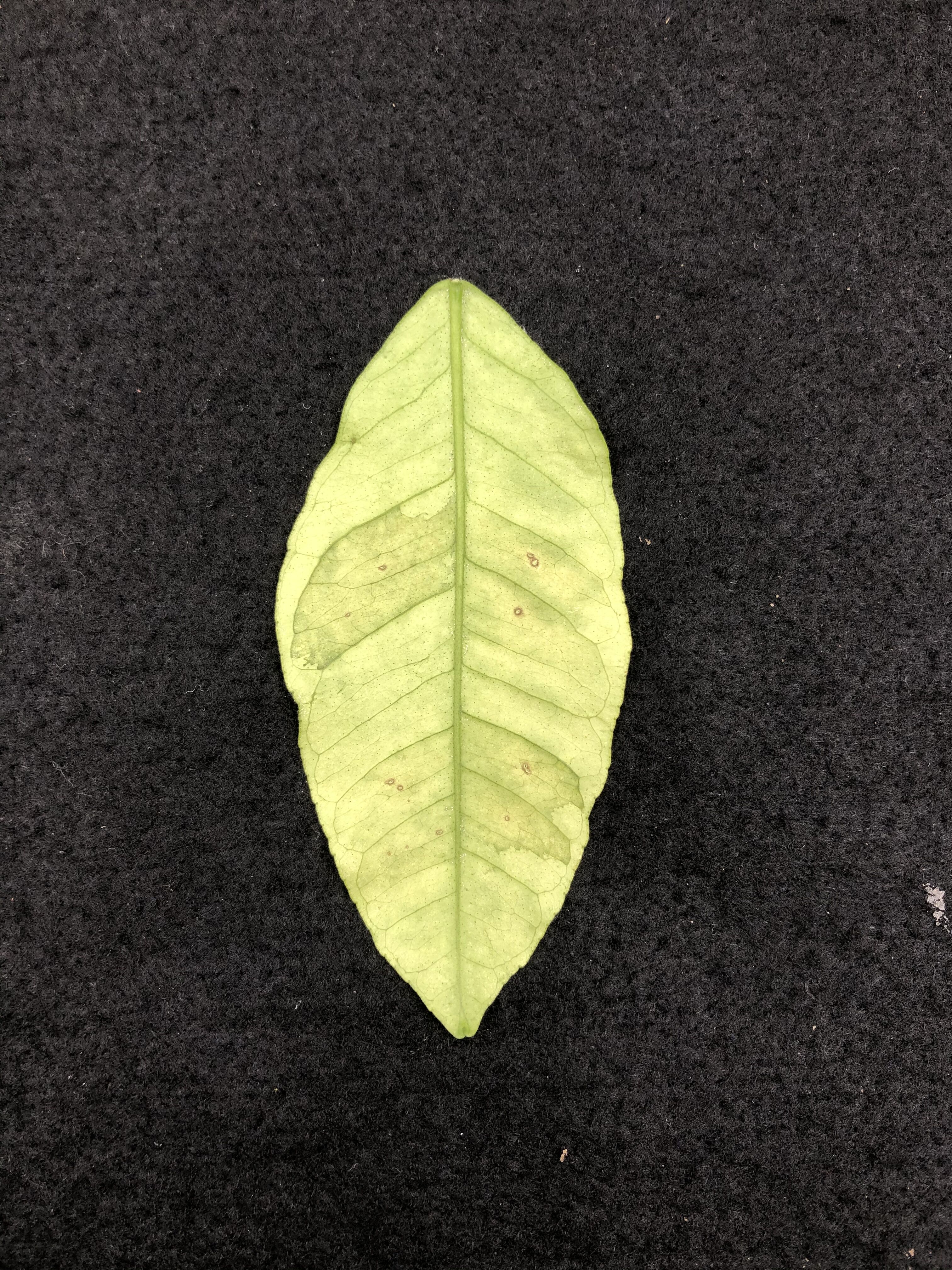

Supplement: Figure 7—source data 11. [file elife-91684-fig7-data11.zip › Figure 7- source data 11/Figure 7- source data 11.jpg]

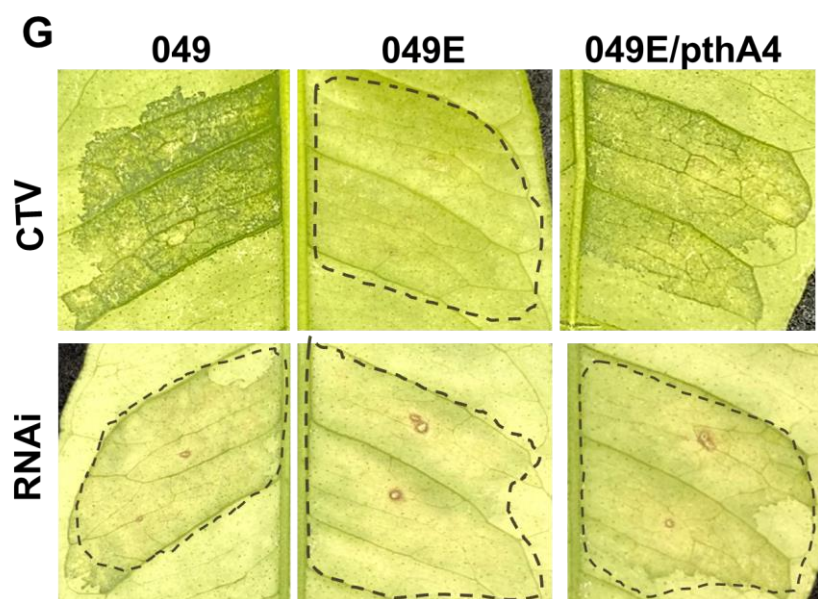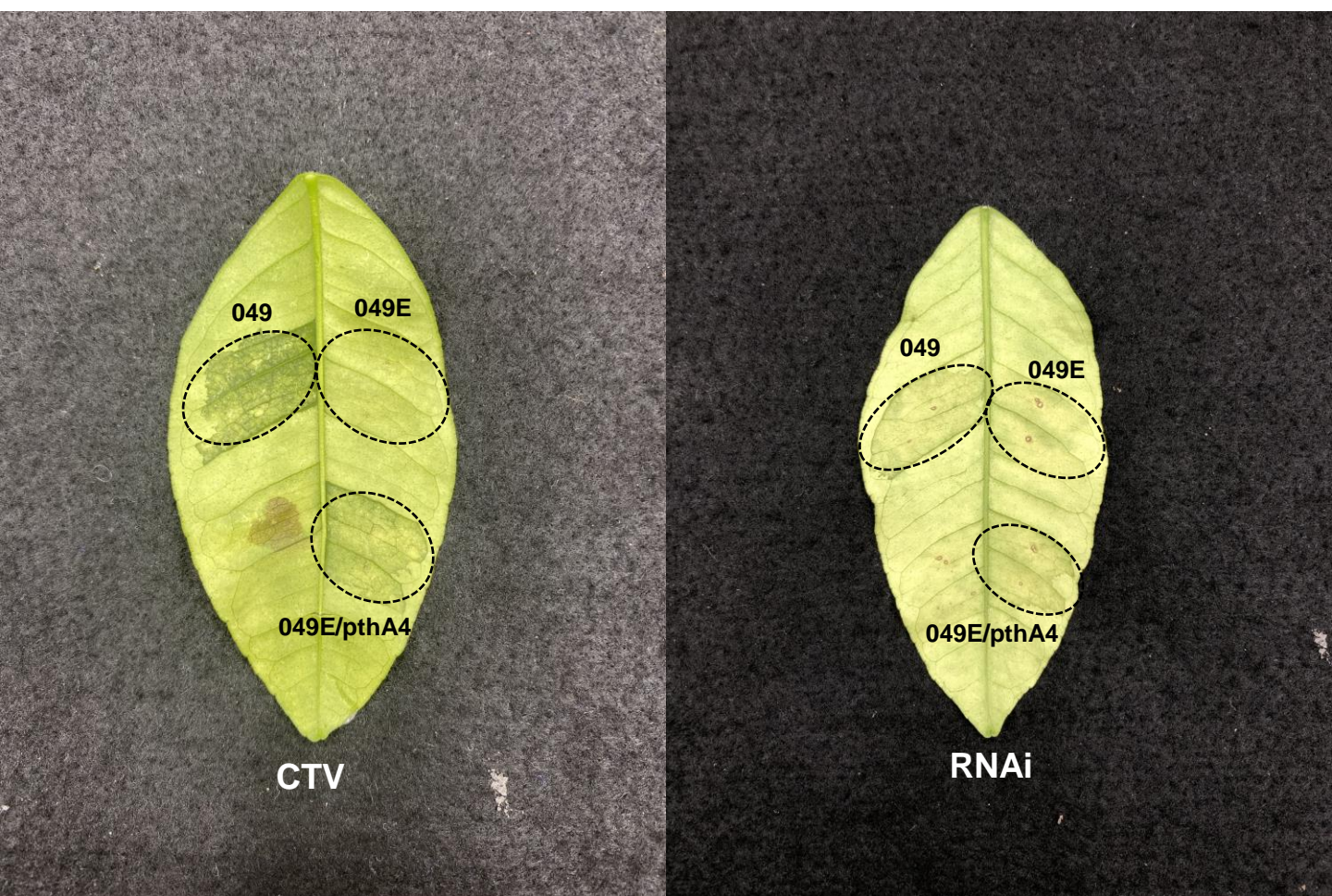

**Figure 7**

Supplement: Figure 7—source data 12. [file elife-91684-fig7-data12.pdf]
