# Supplementary material for: Xanthomonas citri subsp. citri type III effector PthA4 directs the dynamical expression of a putative citrus carbohydrate-binding protein gene for canker formation
Source: eLife. 2024 Aug 13;13:RP91684. doi: 10.7554/eLife.91684 (PMC11321762; doi:10.7554/eLife.91684)
Supplement: Figure 2—source data 9. [file elife-91684-fig2-data9.pdf]

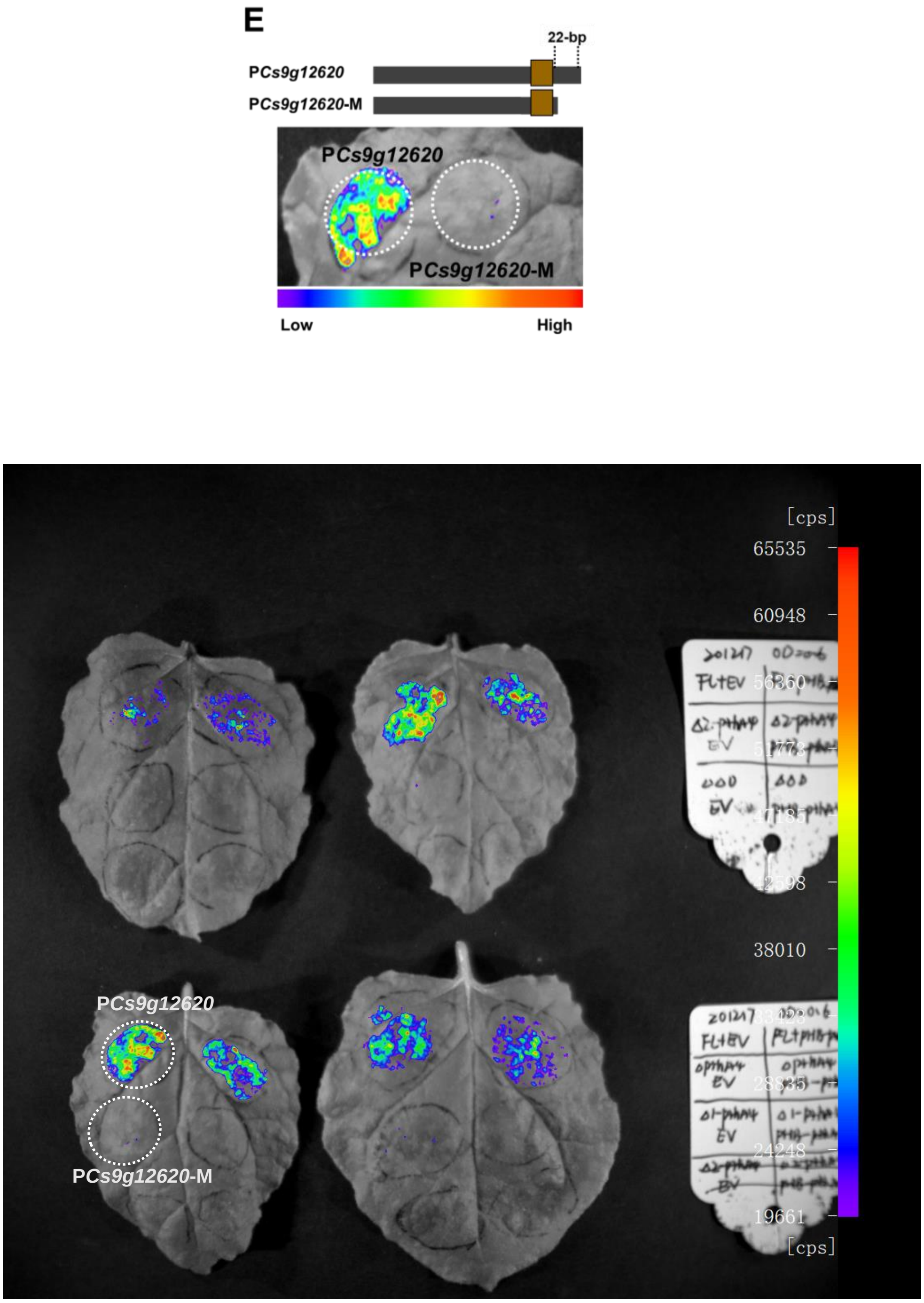
E
22-bp
PCs9g12620
PCs9g12620-M
PCs9g12620
PCs9g12620-M
Low
High
[cps]
65535
60948
38010
24248
19661
[cps]
PCs9g12620
PCs9g12620-M

Figure 2
