# Supplementary material for: Xanthomonas citri subsp. citri type III effector PthA4 directs the dynamical expression of a putative citrus carbohydrate-binding protein gene for canker formation
Source: eLife. 2024 Aug 13;13:RP91684. doi: 10.7554/eLife.91684 (PMC11321762; doi:10.7554/eLife.91684)
Supplement: Figure 4—source data 10. [file elife-91684-fig4-data10.pdf]

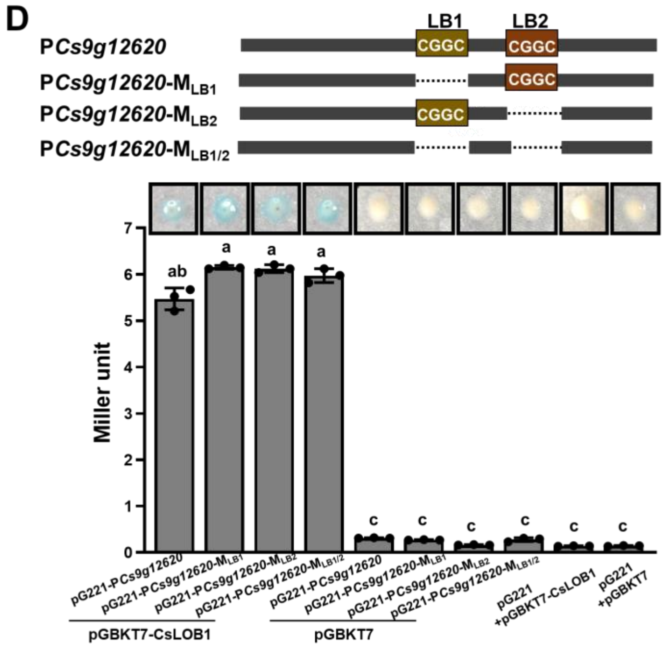
D
LB1
LB2
PCs9g12620
PCs9g12620-M$_{LB1}$
PCs9g12620-M$_{LB2}$
PCs9g12620-M$_{LB1/2}$
CGGC
Miller unit
ab
a
a
a
c
c
c
c
c
c
pG221-PCs9g12620
pG221-PCs9g12620-M$_{LB1}$
pG221-PCs9g12620-M$_{LB2}$
pG221-PCs9g12620-M$_{LB1/2}$
pG221-PCs9g12620
pG221-PCs9g12620-M$_{LB1}$
pG221-PCs9g12620-M$_{LB2}$
pG221-PCs9g12620-M$_{LB1/2}$
pG221 +pGBKT7-CsLOB1
pG221 +pGBKT7
pGBKT7-CsLOB1
pGBKT7

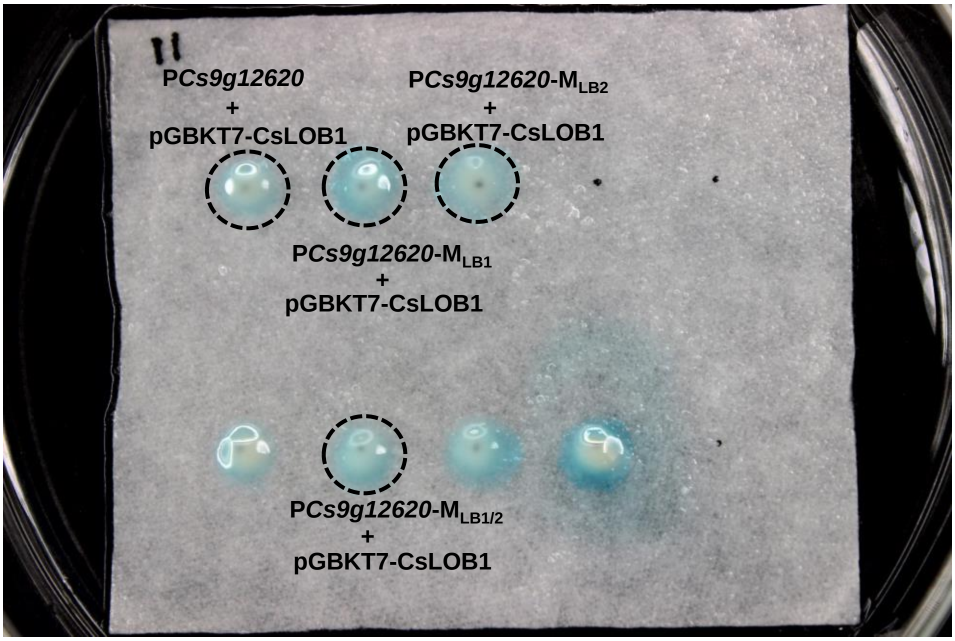
PCs9g12620 + pGBKT7-CsLOB1
PCs9g12620-M$_{LB2}$ + pGBKT7-CsLOB1
PCs9g12620-M$_{LB1}$ + pGBKT7-CsLOB1
PCs9g12620-M$_{LB1/2}$ + pGBKT7-CsLOB1

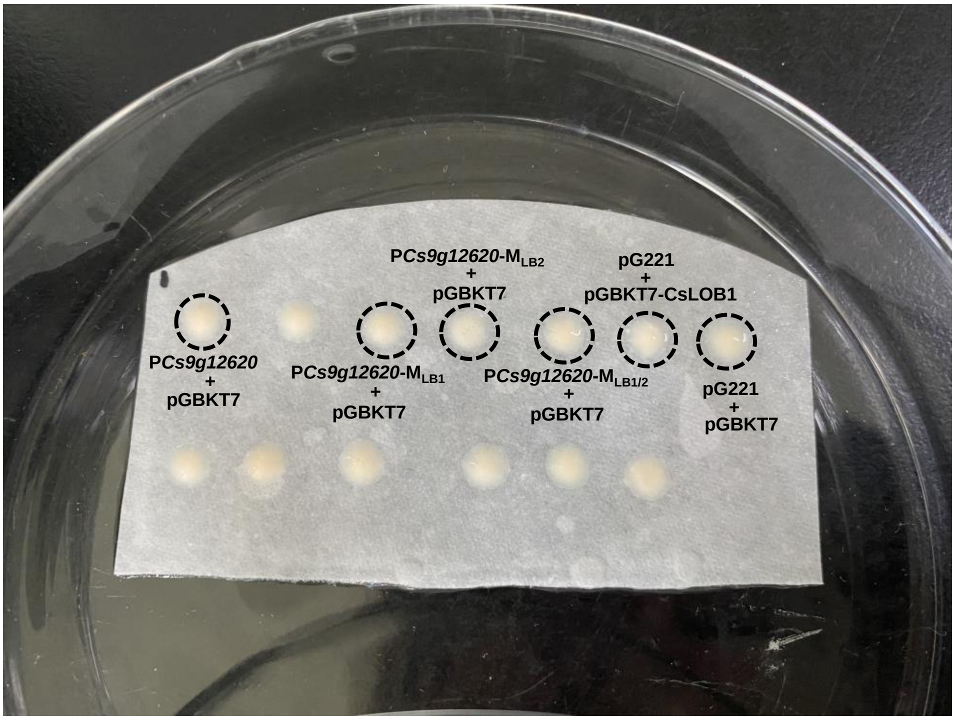
PCs9g12620-M$_{LB2}$ + pGBKT7
pG221 + pGBKT7-CsLOB1
PCs9g12620 + pGBKT7
PCs9g12620-M$_{LB1}$ + pGBKT7
PCs9g12620-M$_{LB1/2}$ + pGBKT7
pG221 + pGBKT7

Figure 4
